# Synthesis and Characterization of Yttrium Methanediide Silanide Complexes

**DOI:** 10.1021/acs.inorgchem.2c03053

**Published:** 2022-12-20

**Authors:** Benjamin L. L. Réant, Ashley J. Wooles, Stephen T. Liddle, David P. Mills

**Affiliations:** Department of Chemistry, The University of Manchester, Oxford Road, Manchester M13 9PL, U.K.

## Abstract

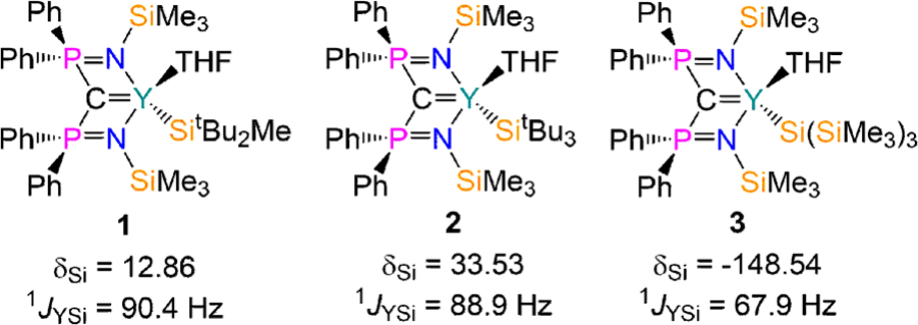

The salt metathesis reactions of the yttrium methanediide iodide complex [Y(BIPM)(I)(THF)_2_] (BIPM = {C(PPh_2_NSiMe_3_)_2_}) with the group 1 silanide ligand-transfer reagents MSiR_3_ (M = Na, R_3_ = ^*t*^Bu_2_Me or ^*t*^Bu_3_; M = K, R_3_ = (SiMe_3_)_3_) gave the yttrium methanediide silanide complexes [Y(BIPM)(Si^*t*^Bu_2_Me)(THF)] (**1**), [Y(BIPM)(Si^*t*^Bu_3_)(THF)] (**2**), and [Y(BIPM){Si(SiMe_3_)_3_}(THF)] (**3**). Complexes **1–3** provide rare examples of structurally authenticated rare earth metal–silicon bonds and were characterized by single-crystal X-ray diffraction, multinuclear NMR and ATR-IR spectroscopies, and elemental analysis. Density functional theory calculations were performed on **1–3** to probe their electronic structures further, revealing predominantly ionic Y–Si bonding. The computed Y–Si bonds show lower covalency than Y=C bonds, which are in turn best represented by Y^+^–C^–^ dipolar forms due to the strong σ-donor properties of the silanide ligands investigated; these observations are in accord with experimentally obtained ^13^C{^1^H} and ^29^Si{^1^H} NMR data for **1–3** and related Y(III) BIPM alkyl complexes in the literature. Preliminary reactivity studies were performed, with complex **1** treated separately with benzophenone, azobenzene, and *N*,*N*′-dicyclohexyl-carbodiimide. ^29^Si{^1^H} and ^31^P{^1^H} NMR spectra of these reaction mixtures indicated that 1,2-migratory insertion of the unsaturated substrate into the Y–Si bond is favored, while for the latter substrate, a [2 + 2]-cycloaddition reaction also occurs at the Y=C bond to afford [Y{C(PPh_2_NSiMe_3_)_2_[C(NCy)_2_]-κ^4^*C*,*N*,*N*′,*N*′}{C(NCy)_2_(Si^*t*^Bu_2_Me)-κ^2^*N*,*N*′}] (**4**); these reactivity profiles complement and contrast with those of Y(III) BIPM alkyl complexes.

## Abstract 2

We report the synthesis and characterization of three yttrium methanediide silanide complexes, which contain rare examples of structurally authenticated highly polarized-covalent yttrium−silicon bonds.

## Introduction

Transition-metal (TM) silicides have been widely used in ceramics, microelectronics, and catalysis as they display high stability and good electrical conductivity.^1^ The chemistry of rare earth (RE) silicides is less developed, and their applications are currently limited to strengthening low-alloy steels.^2^ This is consistent with the current status of RE solution silicon chemistry compared to the extensive array of RE complexes supported by harder C-, N-, and O-donor ligands.^3^ Progress in this field has been relatively slow since the first RE silanide complex [Li(DME)_3_][Sm(Cp)_2_(SiMe_3_)_2_] (Cp = C_5_H_5_) was reported by Schumann in 1985,^4^ with <80 structurally characterized complexes containing RE–Si bonds reported to date.^5^ The majority of these examples are silanide ({R_3_Si}^−^) complexes; this scarcity contrasts with RE alkyl chemistry, which is very well developed.^6−10^ However, in recent years, an increasing number of RE silicon complexes have been prepared, and more comprehensive characterization is being performed in order to better understand their electronic structures and to inform future applications.^11,12^

For yttrium silicon chemistry, structurally authenticated complexes containing Y–Si bonds that have been reported to date include [Y(Cp*)_2_{SiH(SiMe_3_)_2_}] (Cp* = C_5_Me_5_),^13^ [Y{Si(SiMe_3_)_2_R}(I)_2_(THF)_3_] (R = Et or SiMe_3_),^14^ [Y{Si(SiMe_2_H)_3_}_2_(OEt_2_)(μ_2_-Cl)_2_(μ_3_-Cl)K_2_(OEt_2_)_2_]_∞_,^15^ [K(2.2.2-crypt)][Y(C_5_H_4_Me)_3_(SiH_2_Ph)],^16^ [K(DME)_4_][Y(L) (A)_2_(DME)_*n*_] (L = {[Si(SiMe_3_)_2_SiMe_2_]_2_O}, A = Cp, *n* = 0, or A = Cl, *n* = 1;^17^ L = {[Si(SiMe_3_)_2_SiMe_2_]_2_}, A = Cp, *n* = 0, or A = Cl, *n* = 1),^18^ [Y(Cp)_3_{Si[{N(CH_2_^*t*^Bu)}_2_C_6_H_4_-1,2]}],^19^ and [Y{N(SiHMe_2_)_2_}_3_{Si[(N^*t*^Bu)_2_CPh][C_5_H_4_N(NMe-2)]-κ^2^*Si*,*N*}].^20^ Recently, we showed that a combination of ^29^Si{^1^H} NMR spectroscopy and density functional theory (DFT) calculations could be applied to quantify covalency in diamagnetic Yb(II)–Si bonds, allowing comparisons with Mg(II), Ca(II), and *in silico*-calculated No(II) homologs.^21^ To potentially extend this methodology to the predominant +3 oxidation state for RE ions, we are currently limited to diamagnetic closed shell Sc(III), Y(III), La(III), and Lu(III) examples;^3^ recently, solid-state ^29^Si{^1^H} NMR spectroscopy has been used to study a series of La(III) silanide complexes, and coupling to 99.95% abundant *I* = 7/2 ^139^La nuclei was resolved.^22^ We identified that the yttrium methanediide iodide complex, [Y(BIPM)(I)(THF)_2_] (bis(iminophosphorano)methanediide, BIPM = {C(PPh_2_NSiMe_3_)_2_}),^23^ could be a useful precursor to the synthesis of complexes containing Y(III)–Si bonds by salt metathesis protocols as it has already proven to be effective in the stabilization of yttrium–metal/metalloid bonds;^24^^89^Y (100% abundant, *I* = 1/2) is a particularly advantageous isotope to have present in NMR spectroscopic studies. Conversely, the yttrium methanediide alkyl complexes, [Y(BIPM)(CH_2_SiMe_3_)(THF)]^25^ and [Y(BIPM)(CH_2_Ph)(THF)],^26^ have been shown to effect sequential C–C and C–O bond formation reactions with ketones,^27^ providing additional impetus for the synthesis of Y(III) BIPM silanide derivatives to allow direct comparisons with these alkyls.

Here, we report the synthesis of three heteroleptic yttrium methanediide silanide complexes supported by the BIPM scaffold by salt metathesis protocols. All complexes were characterized by multinuclear NMR and ATR-IR spectroscopy, single-crystal X-ray diffraction, and elemental analysis. Density functional theory calculations were performed to probe the electronic structures of these complexes, and a preliminary reactivity study of one of the yttrium methanediide silanide complexes with a selection of unsaturated organic substrates was undertaken. The combination of analytical data obtained shows predominantly electrostatic Y–Si bonds in all three complexes, with relatively minor differences in the electronic structures of the Y(III) centers upon variation of alkyl and silyl substituents of the hypersilanide ligands.

## Results and Discussion

### Synthesis and Spectroscopic Characterization

**Scheme 1:**
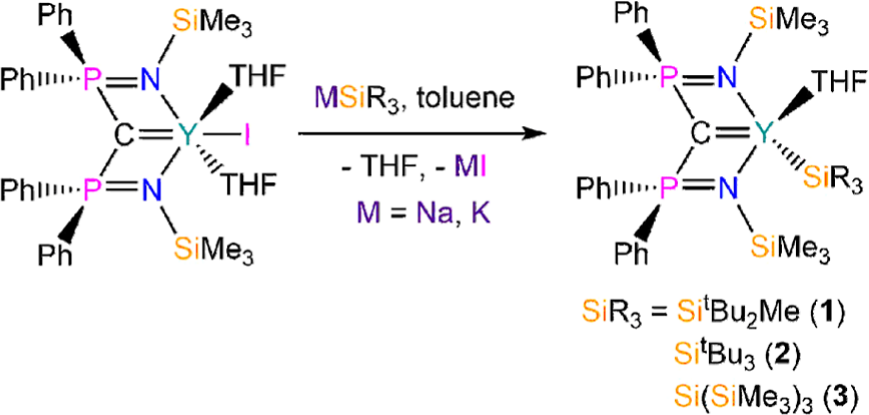
Synthesis of Complexes **1–3**

The separate salt metathesis reactions of [Y(BIPM)(I)(THF)_2_]^23^ with the three group 1 silanide ligand-transfer agents MSiR_3_ (M = Na, R_3_ = ^*t*^Bu_2_Me or ^*t*^Bu_3_; M = K, R_3_ = (SiMe_3_)_3_)^21,28−30^ in toluene gave the colorless yttrium methanediide silanide complexes [Y(BIPM)(Si^*t*^Bu_2_Me)(THF)] (**1**), [Y(BIPM)(Si^*t*^Bu_3_)(THF)] (**2**), and [Y(BIPM){Si(SiMe_3_)_3_}(THF)] (**3**), respectively (Scheme 1). Following work-up and recrystallization from toluene (**1** and **2**) or pentane (**3**), these complexes were obtained in respective yields of 59, 36, and 56%; the lower yield of **2** is attributed to it having a higher solubility in toluene than complex **1** or issues associated with the steric demands of the {Si^*t*^Bu_3_} ligand. The multinuclear NMR spectra of **1–3** showed few impurities; thus, we are confident of the solid-state structures (see below) being representative of their bulk formulations. Carbon values obtained for **1** and **2** in elemental microanalyses were lower than expected values on multiple occasions, which we attribute to the formation of silicon carbides that do not fully combust;^31^ we have observed this phenomenon consistently for early metal silanide complexes of the ligands used here.^21,32^ ATR-IR spectroscopy was also performed on **1–3**, with a number of overlapping absorptions observed showing that some similar vibrational modes are present in all three complexes (see Supporting Information Figures S1–S25 for spectroscopic data of **1–3**).

The ^1^H NMR spectra of **1–3** were fully assigned, with integrals confirming the retention of a single molecule of bound THF in each case. Residual toluene from the crystal lattice (see below) was observed in the ^1^H and ^13^C{^1^H} NMR spectra of **1** but not for **2**, and a trace amount of trapped pentane lattice solvent was seen in the corresponding spectra of **3**. A complex set of multiplets was observed in the aromatic regions of both ^1^H and ^13^C{^1^H} NMR spectra of **1–3** for the P(V) phenyl groups; the relatively large number of signals indicates that a local C_1_ symmetry is adopted in solution, which is typical for Y(III) BIPM complexes.^23−27,33,34^ The most distinctive feature of the ^13^C{^1^H} NMR spectra of **1–3** is the doublet of triplet resonances for the bound methanediide resonances that arise from coupling to *I* = 1/2 ^31^P and ^89^Y nuclei (**1**: δ_C_ = 71.02 ppm, ^1^*J*_PC_ = 128.5 Hz, ^1^*J*_YC_ = 6.6 Hz; **2**: δ_C_ = 72.81 ppm, ^1^*J*_PC_ = 120.1 Hz, ^1^*J*_YC_ = 7.4 Hz; **3**: δ_C_ = 72.45 ppm, 1*J*_PC_ = 120.4 Hz, ^1^*J*_YC_ = 7.5 Hz). These resonances are deshielded, the ^1^*J*_PC_ coupling constants are relatively small, and ^1^*J*_YC_ values are large, compared to related Y(III) BIPM alkyl complexes ([Y(BIPM) (CH_2_SiMe_3_) (THF)]:^25^ δ_C_ = 60.08 ppm, ^1^*J*_PC_ = 131.9 Hz, ^1^*J*_YC_ = 4.9 Hz; [Y(BIPM)(CH_2_Ph)(THF)]:^26^ δ_C_ = 61.8 ppm, ^1^*J*_PC_ = 207.3 Hz, ^1^*J*_YC_ = 5.0 Hz). These data suggest that the formal Y=C bond is retained in solution.

The doublet resonances observed in the ^31^P{^1^H} NMR spectra of **1–3** are relatively deshielded, and the ^2^*J*_YP_ coupling constants are large compared to the literature alkyl complexes (**1**: δ_P_ = 8.35 ppm, ^2^*J*_YP_ = 13.2 Hz; **2**: δ_P_ = 9.41 ppm, ^2^*J*_YP_ = 13.8 Hz; **3**: δ_P_ = 9.25 ppm, ^2^*J*_YP_ = 13.7 Hz; cf. [Y(BIPM)(CH_2_SiMe_3_)(THF)]:^25^ δ_P_ = 7.30 ppm, ^2^*J*_YP_ = 11.5 Hz; [Y(BIPM)(CH_2_Ph)(THF)]:^26^ δ_P_ = 4.80 ppm, ^2^*J*_YP_ = 13.1 Hz). The ^29^Si{^1^H} NMR spectra of **1–3** each exhibit signals for both the BIPM SiMe_3_ groups and the metal-bound silanide atoms, with **3** showing an additional resonance for the silanide SiMe_3_ substituents. The silanide signals are doublets from coupling to ^89^Y (**1**: δ_Si_ = 12.86 ppm, ^1^*J*_YSi_ = 90.4 Hz; **2**: δ_Si_ = 33.53 ppm, ^1^*J*_YSi_ = 88.9 Hz; **3**: δ_Si_ = −148.54 ppm, ^1^*J*_YSi_ = 67.9 Hz), with the coupling constants comparable to those previously reported for yttrium silanide complexes, for example, [Y(Cp*)_2_{SiH(SiMe_3_)_2_}] (δ_Si_ = −120.00 ppm, ^1^*J*_YSi_ = 92 Hz)^13^ and [Y{Si(SiMe_3_)_2_R}(I)_2_(THF)_3_] (R = Et, δ_Si_ = −73.50 ppm, ^1^*J*_YSi_ = 71.0 Hz; SiMe_3_, δ_Si_ = −134.68 ppm, ^1^*J*_YSi_ = 63.4 Hz),^14^ and greater than those seen for the yttrium silylenes [Y(Cp)_3_{Si[{N(CH_2_^*t*^Bu)}_2_C_6_H_4_-1,2]}] (δ_Si_ = 119.5 ppm, ^1^*J*_YSi_ = 59 Hz)^19^ and [Y{N(SiHMe_2_)_2_}_3_{Si[(N^*t*^Bu)_2_CPh][C_5_H_4_N(NMe-2)]-κ^2^*Si*,*N*}] (δ_Si_ = 9.4 ppm, ^1^*J*_YSi_ = 55 Hz).^20^ The positive δ_Si_ values for alkyl-substituted silanides and negative values for the silyl-substituted silanides, together with larger metal–silicon coupling constants for the former ligand sets, arise from alkyl substituents being more electron-donating than their silyl counterparts, which additionally exhibit negative hyperconjugation.^12,35^ These data are in accord with previous observations on related Yb(II) silanide complexes containing *I* = 1/2 ^171^Yb nuclei (e.g., [Yb(Si^*t*^Bu_2_Me)_2_(THF)_3_]:^21^ δ_Si_ = 29.24 ppm, ^1^*J*_YbSi_ = 921 Hz; [Yb(Si^*t*^Bu_3_)_2_(THF)_2_]:^21^ δ_Si_ = 54.19 ppm, ^1^*J*_YbSi_ = 976 Hz; [Yb{Si(SiMe_3_)_3_}_2_(THF)_3_]:^35^ δ_Si_ = −144.8 ppm, ^1^*J*_YbSi_ = 723 Hz). We attempted to collect an ^89^Y NMR spectrum of complex **1**, but we were unable to observe a signal. ^89^Y NMR spectra of organometallic complexes have been reported previously, but these measurements are often hindered in the solution phase by low receptivity and long relaxation times; solid-state ^89^Y NMR spectroscopy has proved advantageous.^36^ Rich solid-state ^29^Si{^1^H} NMR spectra showing coupling to ^139^La nuclei have recently been disclosed for a series of La silanide complexes.^22^

### Structural Characterization

**Figure 1:**
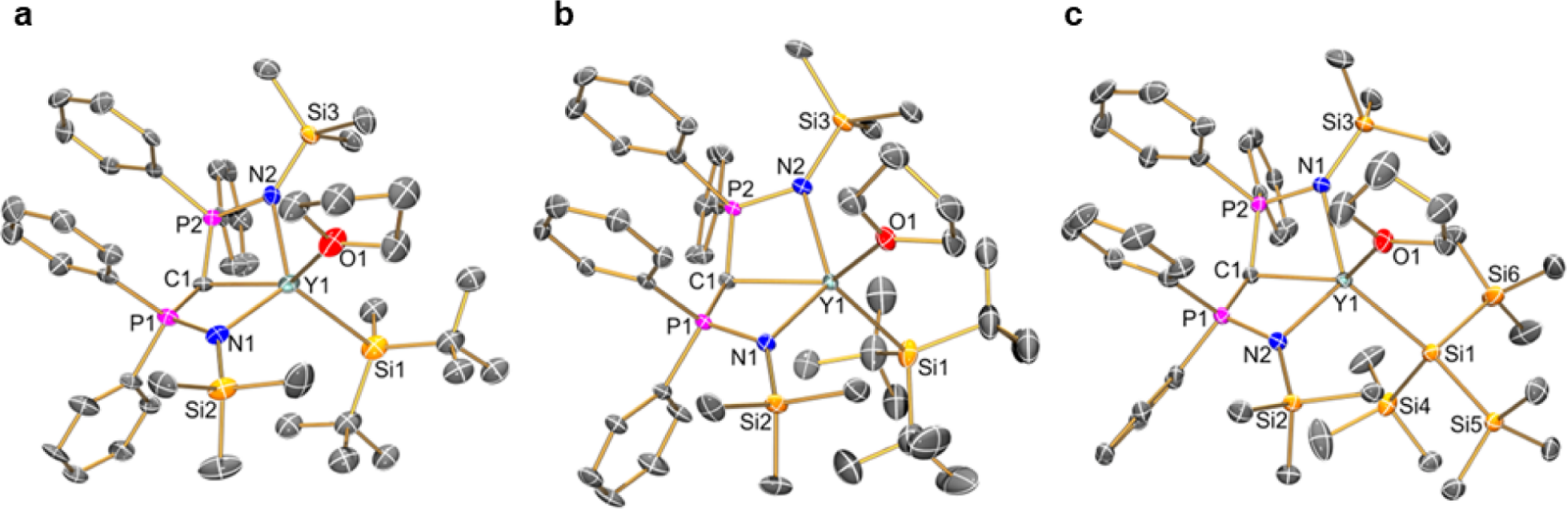
Molecular structures of (a) **1·toluene**, (b) **2·toluene**, and (c) **3·0.5pentane**, with selective atom labeling and displacement ellipsoids set at the 30% probability level (**1·toluene**, **2·toluene**) or 50% probability level (**3·0.5pentane**); hydrogen atoms, lattice solvent, and disorder components have been omitted for clarity.

The solid-state structures of **1·toluene**, **2·toluene**, and **3·0.5pentane** were confirmed by XRD studies of single crystals grown from saturated toluene or pentane solutions (Figure 1 and Table 1; crystallographic parameters are compiled in the Supporting Information Table S1). All three complexes exhibit similar structures, with BIPM in a standard tridentate binding mode and the Y(III) coordination spheres completed by one silanide and one bound THF molecule to give a near-C_*s*_-local symmetry; the data set obtained for **1·toluene** is poor due to weak diffraction; thus, we do not comment on metrical parameters, but as the connectivity is clear-cut, we include these data for completeness as part of a structurally analogous series of complexes. The BIPM scaffolds adopt typical “open-book” conformations upon coordination to yttrium,^33,34^ and the statistically identical Y=C methanediide distances of **1–3** (range: 2.338(11)–2.361(2) Å) are similar to those previously found for related alkyl complexes (e.g., [Y(BIPM)(CH_2_SiMe_3_)(THF)]: 2.406(3) Å;^25^ [Y(BIPM)(CH_2_Ph)(THF)]: 2.357(3) Å).^26^ The Y–Si bond distances of **1–3** (range: 3.0126(7)–3.062(2) Å) are comparable to those reported previously for literature examples of yttrium silanide complexes, for example, [Y{Si(SiMe_3_)_2_R}(I)_2_(THF)_3_] (R = Et, 2.961(2) Å; R = SiMe_3_, 2.979(3) Å),^14^ [Y{Si(SiMe_2_H)_3_}_2_(OEt_2_)(μ_2_-Cl)_2_(μ_3_-Cl)K_2_(OEt_2_)_2_]_∞_ (3.035(1) Å),^15^ and [K(2.2.2-crypt)][Y(C_5_H_4_Me)_3_(SiH_2_Ph)] (2.9531(7) Å);^16^ it is noteworthy that the Y–Si distance of **2** (3.062(2) Å) is longer than that of **3** (3.0126(7) Å), which we attribute to a combination of the Si–Si bonds in the silanide of **3** being longer than the silanide Si–C bonds present in **2** to provide a reduced steric effect, together with differences in crystal packing effects. All other intra-ligand bond distances and angles of BIPM frameworks in **1–3** are consistent with those of previously reported Y(III) BIPM complexes.^33,34^

**Table 1:** Selected Bond Distances (Å) and Angles (°) for **1–3**

	1·toluene	2·toluene	3·0.5pentane
Y–Si	3.017(10)(mean)	3.062(2)	3.0126(7)
Y=C	2.338(11)	2.357(6)	2.361(2)
Y–N_mean_	2.314(11)	2.365(5)	2.325(3)
Si–Y=C	111.8(6)(mean)	111.8(2)	116.30(6)
N–Y–N	122.7(3)	124.2(2)	123.43(6)
P–C–P	135.4(7)	139.3(4)	135.22(14)

### Computational Studies

Restricted DFT calculations were performed on **1–3** to study both the Y–Si and Y=C bonds in greater detail. The geometry-optimized structures effectively reproduced the experimentally observed solid-state structures (Table 1, see Supporting Information Tables S3–S5 for optimized geometry coordinates for **1–3**); this gives confidence that the computational models provide internally consistent representations of the electronic structures of **1–3** and that qualitative comparisons can be reliably made. The calculated Y–Si bond distances (range: 3.055–3.103 Å) are consistently slightly longer than those observed in the corresponding single-crystal X-ray diffraction (XRD) data, with the model of **3** exhibiting the shortest Y–Si bond of the three complexes due to the lower steric requirements of SiMe_3_ versus ^*t*^Bu groups (see above). As expected, the Nalewajski–Mrozek bond indices of the Y–Si bonds (range: 0.62–0.63) are smaller than those of the Y=C bonds (range: 0.73–0.77). Although the multipole derived charges (MDC-*q*) on Y and C do not show much variance between **1–3** and while *q*_Si_ is similar for **1** (0.35) and **2** (0.31) due to the fact that both contain electron-donating alkyl substituents, negative hyperconjugation from silyl substituents leads to the value for **3** being more negative (*q*_Si_ = −0.40).^37,38^

**Figure 2:**
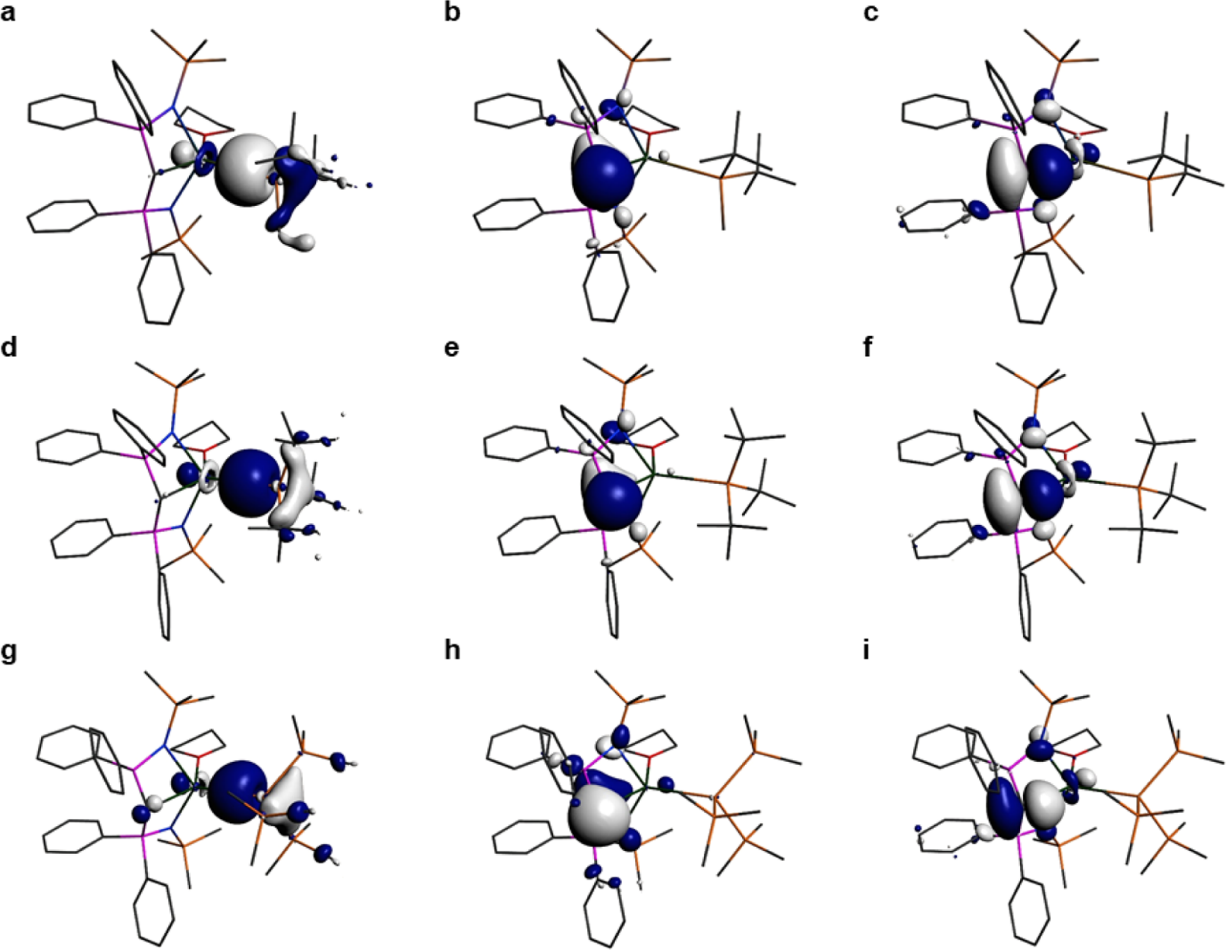
Selected Kohn–Sham molecular orbitals for **1** (a): HOMO (−3.824 eV), (b) HOMO – 1 (−4.559 eV), and (c) HOMO – 2 (230, −4.892 eV); **2** (d) HOMO (−3.746 eV), (e) HOMO – 1 (−4.563 eV), and (f) HOMO – 2 (−4.962 eV); **3** (g) HOMO (−4.248 eV), (h) HOMO – 1 (−4.644 eV), and (i) HOMO – 4 (−5.048 eV). Hydrogen atoms are omitted for clarity.

Natural bond orbital (NBO) analysis shows that the bonding is predominantly electrostatic, as is typical for interactions between rare earth ions and ligand donor atoms in complexes, including those containing RE–Si bonds,^3,11,12,22^ yet there is surprisingly little variation between **1–3** (see Figure 2 for Kohn–Sham orbital representations of selected frontier orbitals of **1–3** and Supporting Information Figures S26–S28 for NBO depictions). The pyramidalization of the methanediide carbon atoms in **1–3** leads to the orthogonal π-components of the Y=C bonds being all essentially localized s/p-hybrid orbitals at C; thus, unlike [Y(BIPM)(I)(THF)_2_] for which a Y=C double-bond interaction description is reasonable,^23^ a formal Y^+^–C^–^ dipolar bond description is appropriate, in common with [Y(BIPM)(CH_2_SiMe_3_)(THF)].^25^ The near pure 2p-orbital at C is oriented toward Y to provide the σ-component of the Y=C bonds; each of these latter interactions contains 6% contributions from an almost pure 4d-orbital at Y. The relatively highly localized electron density at the methanediide centers in **1–3** compared to the majority of Y(III) BIPM complexes in the literature^33,34^ can be attributed to the strongly donating silanide substituents. All Y–Si σ-bonds consist of s/p-hybridized lone pairs that are mainly localized at the silanide center, with the largest Y contribution of 17% seen for the sterically least demanding silanide in **1**; this is essentially an s/d hybrid orbital, and although the Y contribution is below the NBO program 5% cutoff for **2** and **3**, the highest occupied molecular orbitals (HOMOs) look similar in Kohn–Sham representations of **1–3** (Figure 2). The HOMOs of **1–3** are the Y–Si bonds (**1**: −3.824 eV; **2**: −3.746 eV; **3**: −4.248 eV); analogously, the Y–C bond of [Y(BIPM)(CH_2_Ph)(THF)] is the HOMO (−4.248 eV), although for the silyl-substituted alkyl complex [Y(BIPM)(CH_2_SiMe_3_)(THF)], the HOMO is the π-component of the Y=C bond localized at C.^25^

Quantum theory of atoms in molecules (QTAIM) analyses show highly polarized-covalent bonds in **1–3**, with more covalent Y=C than Y–Si bonds for each complex (Table 2). The positive Laplacian values, ∇^2^ρ(*r*), are meaningless for heavy-atom complexes,^39,40^ while the electronic energy densities, *H*(*r*), are negative as expected. The bond ellipticities, ε(*r*), show cylindrical Y–Si single σ-bonds (range: 0.02–0.03), whereas the Y=C bonds show a moderate deviation toward asymmetric double bonds (cf. Y=C range: 0.13–0.16) but are around half the value required before double-bond character can be credibly invoked. This is consistent with the bond indices, a visual inspection of the MOs and NBOs, and the NBO data.

**Table 2:** Selected Computed DFT, NBO, and QTAIM Data for the Y–Si and Y=C Bonds in **1–3**

	bond length and index^b^^,^^c^			charges			NBO σ-component^g^				NBO π-component^g^^,^^h^	QTAIM^i^			
entry^a^	bond	bond length	bond Index	*q*_Y_^d^	*q*_Si_^e^	*q*_C_^f^	Y [%]	Si/C[%]	Y 5s/5p/4d [%]	C/Si *n*s/*n*p [%]	C 2s/2p [%]	ρ(r)	∇^2^ρ(r)	*H*(r)	ε(r)
1	Y–Si	3.060	0.62	1.31	0.35	–1.49	17	83	56/1/43	32/68	26/74	0.04	0.02	–0.01	0.03
	Y=C	2.372	0.73				6	94	0/2/98	1/99		0.07	0.13	–0.02	0.13
2	Y–Si	3.103	0.62	1.28	0.31	–1.45	0	100		48/52	28/72	0.04	0.01	–0.01	0.02
	Y=C	2.361	0.77				6	94	0/2/98	1/99		0.07	0.13	–0.02	0.14
3	Y–Si	3.055	0.63	1.37	–0.40	–1.42	0	100		48/52	28/72	0.03	0.02	–0.08	0.02
	Y=C	2.377	0.77				6	94	0/2/98	1/99		0.07	0.13	–0.02	0.16

a All molecules geometry-optimized without symmetry constraints at the LDA VWN BP86 TZP/ZORA level.

b Calculated bond of interest.

c Nalewajski–Mrozek bond indices.

d Multipole derived charges (MDC-*q*) on Y.

e MDC-*q* charge on Si.

f MDC-*q* charge on C.

g Natural bond orbital (NBO) analyses.

h Y = 0% and C = 100% contributions to Y=C π-bond in **1–3**.

i Quantum theory of atoms in molecules (QTAIM) topological electron density [ρ(*r*)], Laplacian [∇^2^ρ(*r*)], electronic energy density [*H*(*r*)], and ellipticity [ε(*r*)] 3, −1 bond critical point data.

### Reactivity Studies

To probe the Y–Si bond further, we performed a preliminary reactivity study on complex **1** as a representative example. We selected three unsaturated substrates that were previously shown to affect 1,2-migratory insertions at the Y–C alkyl bond of [Y(BIPM)(CH_2_Ph)(THF)]: benzophenone, azobenzene, and *N*,*N*′-dicyclohexyl-carbodiimide.^26,41^ In all cases, equimolar reactions were performed in *d*_6_-benzene at room temperature and ^1^H, ^13^C{^1^H}, ^29^Si{^1^H}, and ^31^P{^1^H} NMR spectra were recorded after 8 h; a second equivalent of substrate was then added, and multinuclear NMR spectra were recorded a second time after 8 h. All spectra are compiled in Supporting Information Figures S29–S52, and observations for each substrate are described below.

#### Ph_2_CO

The 1:1 reaction of **1** with benzophenone gave an intense red reaction mixture upon addition of a solvent, indicating that ligand to metal charge transfer occurs upon coordination of benzophenone to the Y center, as observed previously for related Y(III) BIPM complexes;^26,27,41^ within ca. 1 h, the color changed to pale yellow, showing that a reaction had occurred. Both the ^29^Si{^1^H} and ^31^P{^1^H} NMR spectra of this reaction mixture contain a relatively large number of signals, indicating the formation of a mixture of products. Yttrium-containing complexes could not be confidently assigned, although several doublets in the ^31^P{^1^H} NMR spectrum had coupling constants that are consistent with ^2^*J*_YP_ for Y(III) BIPM complexes,^33,34^ and the lack of a doublet in the ^29^Si{^1^H} NMR spectrum indicated that the Y–Si bond had been ruptured (singlets at δ_Si_ = 13.07, 11.37, 10.73, 9.66, 9.32, 5.04, −9.02, −9.78 ppm; a signal at −21.82 ppm was attributed to trace silicon grease). A diagnostic triplet of doublets resonance for a methanediide or methanide group could not be assigned in the ^13^C{^1^H} NMR spectrum. This divergent reactivity profile is in contrast with related Y(III) BIPM alkyl complexes, which react 1:1 with benzophenone almost exclusively at the Y–C_alkyl_ bond to give aryloxides as 1,2-migratory insertion products;^26,27,41^ similarly, benzophenone was previously shown to undergo a 1,2-migratory insertion into the Sm–Si bond of [Sm(Cp*)_2_(SiH_3_)(L)] (L = Lewis base).^42^ The Y=C bonds of Y(III) BIPM aryloxide and iodide complexes have previously been shown to promote regioselective C–H activation and C–C bond formation reactions with benzophenone to form substituted *iso*-benzofurans and their isomers.^27^ The addition of a further 1 equiv of benzophenone to the reaction mixture herein led to the growth of a broad signal at δ_P_ = 18.71 ppm in the ^31^P{^1^H} NMR spectrum; this is characteristic of conversion to a dinuclear complex with a bridging methanediide and has previously been shown to be promoted by the addition of benzophenone to a Y(III) BIPM aryloxide.^26^ Given the large number of products formed, we did not scale up the reaction of **1** with 2 equiv of benzophenone.

#### PhN=NPh

A dark-green reaction mixture was obtained immediately upon addition of a solvent to an equimolar mixture of **1** and azobenzene; this color change was previously observed in the reaction of [Y(BIPM)(CH_2_Ph)(THF)] with the same substrate.^26^ Two doublets of similar intensity were observed in the ^31^P{^1^H} NMR spectrum at 6.78 and 1.53 ppm, with coupling constants of 13.0 and 11.3 Hz, respectively; these are tentatively assigned as ^2^*J*_YP_ couplings due to their similarity to other known Y BIPM complexes.^33,34^ The presence of two signals in the ^31^P{^1^H} NMR spectrum indicates that two different products have formed, which is surprising as the 1:1 reaction of azobenzene with [Y(BIPM)(CH_2_Ph)(THF)] was previously shown to afford [Y(BIPM){N(Ph)N(Ph)(CH_2_Ph)-κ^2^*N*,*N*′}(THF)] (δ_P_: 6.30 ppm; ^2^*J*_YP_ = 13.0 Hz) as the only isolable product via a 1,2-migratory insertion.^26^ The ^29^Si{^1^H} NMR spectrum of the reaction mixture showed singlets at 13.43, 12.32, 9.50, −8.15, and −10.01 ppm as well as a signal for trace silicon grease. Two ^29^Si signals are expected for a complex showing approximate C_2_ local symmetry containing both BIPM and a {Si^*t*^Bu_2_Me} moiety; thus, the additional signals are in accord with the ^31^P{^1^H} NMR spectrum being diagnostic for at least two reaction products. Signals with positive δ_Si_ values should be associated with {Si^*t*^Bu_2_Me} groups, and the two upfield δ_Si_ values should correspond to the SiMe_3_ groups of BIPM by comparison with **1**; the lack of doublet resonances indicates rupture of the Y–Si bond. No doublet of triplet resonance was observed in the ^13^C{^1^H} NMR spectrum that could be assigned to a methanediide or methanide center. From these NMR data, we propose that a 1,2-migratory insertion of azobenzene into the Y–Si bond of **1** has occurred to give a product analogous to [Y(BIPM){N(Ph)N(Ph)(CH_2_Ph)-κ^2^*N*,*N*′}(THF)]^26^ but also that a second product formed, which could not be assigned. A second equivalent of azobenzene was added to the reaction mixture, leading to the formation of a single major product with δ_P_ = 1.53 ppm (d, *J* = 11.3 Hz) and δ_Si_ = 9.51 and −10.01 ppm. The reaction of **1** with 2 equiv of azobenzene was scaled up, but we were unable to isolate any products.

#### CyN=C=NCy

No color change was observed in the 1:1 reaction of **1** with *N*,*N*′-dicyclohexyl-carbodiimide. Three doublets were observed in the ^31^P{^1^H} NMR spectrum of approximately equal intensity (δ_P_: 16.79 ppm, *J* = 3.2 Hz; 6.76 ppm, *J* = 13.0 Hz; 2.61 ppm, *J* = 11.3 Hz). The high-field resonances are consistent with retention of the Y=C bond, and the low-field resonance is consistent with a methanide;^33,34^ we attribute one of the high-field signals to a complex formed by 1,2-migratory insertion into the Y–Si bond, and the low-field signal is associated with a complex formed from a [2 + 2]-cycloaddition of the heteroallene at the Y=C bond to form a methanide. The Y(III) BIPM alkyl complex [Y(BIPM)(CH_2_Ph)(THF)] has been shown to react with either 1 or 2 equiv of *N*,*N*′-dicyclohexyl-carbodiimide to solely give [Y{C(PPh_2_NSiMe_3_)_2_[C(NCy)_2_]-κ^4^*C*,*N*,*N*′,*N*′}{C(NCy)_2_(CH_2_Ph)-κ^2^*N*,*N*′}] from concomitant 1,2-migratory insertion and [2 + 2]-cycloaddition reactions,^41^ and the 1,2-migratory insertion of carbodiimides into Y–Si bonds has also previously been observed. The ^29^Si{^1^H} NMR spectrum has singlets at 13.47, 12.33, 7.08, 5.39, −5.95, −8.15, and −11.23 ppm. As with the other reactivity studies, it was not possible to confidently assign methanediide or methanide resonances in the ^13^C{^1^H} NMR spectrum of the reaction mixture. The addition of a second equivalent of *N*,*N*′-dicyclohexyl-carbodiimide lead to the reaction going to completion to form the expected 2:1 product, with one major signal in the ^31^P{^1^H} NMR spectrum (δ_P_: 18.36 ppm, ^2^*J*_YP_ = 4.9 Hz) and two signals in the ^29^Si{^1^H} NMR spectrum at δ_Si_ = 7.07 (*Si*^*t*^Bu_2_Me) and −5.95 (*Si*Me_3_) ppm; a ^3^*J*_YSi_ coupling constant was previously seen for the related Y(III) complex [Y{C(NCy)_2_[Si(SiMe_3_)_2_(Et)]-κ^2^*N*,*N*′}(I)_2_(THF)_2_] (δ_Si_: −54.95 ppm, ^3^*J*_Ysi_ ≈ 5 Hz).^14^

**Figure 3:**
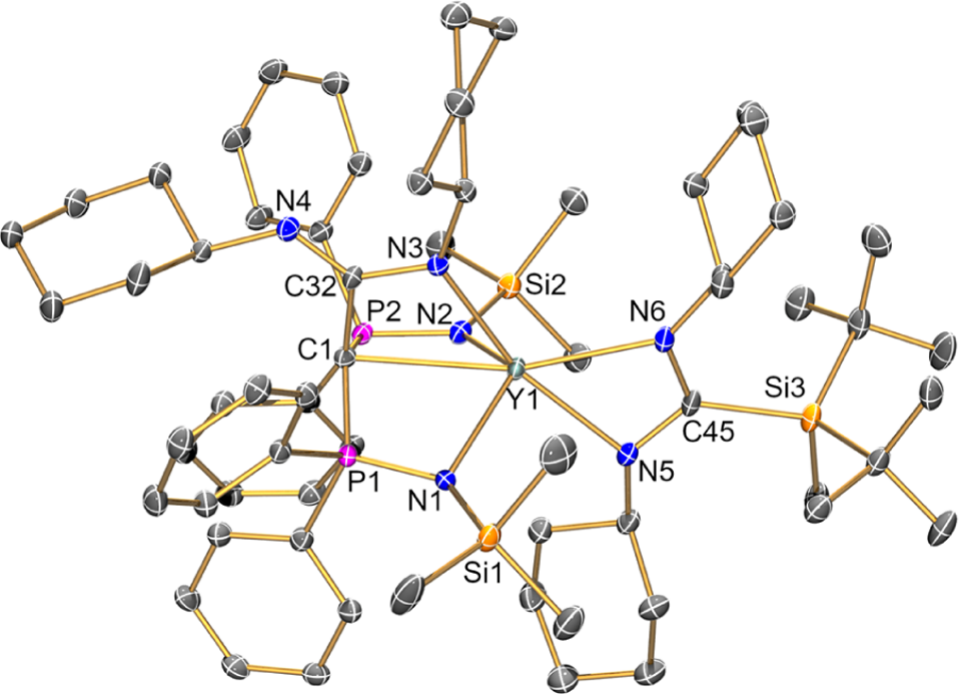
Molecular structure of **4·1.5*d***_**6**_**-benzene**, with selective atom labeling and displacement ellipsoids set at the 30% probability level; hydrogen atoms, lattice solvents, and disorder components have been omitted for clarity. Selected bond distances (Å) and angles (deg): Y(1)–C(1): 2.702(2); Y(1)–N(1) 2.339(2); Y(1)–N(2) 2.367(2); Y(1)–N(3) 2.342(2); Y(1)–N(5): 2.388(2); Y(1)–N(6): 2.386(2); Si(3)–C(45): 2.000(3); C(1)–P(1): 1.747(3); C(1)–P(2): 1.755(2); P(1)–N(1): 1.615(2); P(2)–N(2): 1.619(2); C(1)–C(32): 1.553(4); C(32)–N(3): 1.378(3); C(32)–N(4): 1.296(3); C(45)–N(5): 1.345(4); C(45)–N(6): 1.358(4); N(1)–Y(1)–N(2): 115.87(8); N(5)–Y(1)–N(6): 55.59(8); C(1)–Y(1)–N(3): 56.36(7); P(1)–C(1)–P(2): 129.9(2); C(1)–C(32)–N(3): 110.0(2); N(5)–C(45)–N(6): 110.9(2).

Upon addition of the second equivalent of *N*,*N*′-dicyclohexyl-carbodiimide to **1**, crystals rapidly formed; these were identified as [Y{C(PPh_2_NSiMe_3_)_2_[C(NCy)_2_]-κ^4^*C*,*N*,*N*′,*N*′}{C(NCy)_2_(Si^*t*^Bu_2_Me)-κ^2^*N*,*N*′}]·1.5C_6_D_6_ (**4·1.5*d***_**6**_**-benzene**) by single-crystal XRD (Figure 3). The Y(III) center of **4** is six-coordinate, with the tripodal methanide ligand coordinating with one C- and three N-donor atoms and the bidentate silyl amidinate derivative binding with both N atoms. The N_2_CSi and N_2_C_2_ moieties are both bound nearly orthogonally to the mean plane of the BIPM-derived P_2_N_2_ fragment; the methanide ligand contains localized C–N and C=N bonds, with the imine not bound to the Y(III) center. The Y(III) coordination sphere and the metrical parameters of the {C(PPh_2_NSiMe_3_)_2_[C(NCy)_2_]-κ^4^*C*,*N*,*N*′,*N*′} scaffold in **4** are analogous to those previously observed for [Y{C(PPh_2_NSiMe_3_)_2_[C(NCy)_2_]-κ^4^*C*,*N*,*N*′,*N*′}{C(NCy)_2_(CH_2_Ph)-κ^2^*N*,*N*′}];^41^ the {C(NCy)_2_(Si^*t*^Bu_2_Me)-κ^2^*N*,*N*′} ligand in **4** (mean Y–N: 2.387(3) Å) binds similarly to the amidinate ligand in the literature complex (mean Y–N: 2.410(4) Å), although as the trialkylsilyl group is more electron-donating than a benzyl substituent, the mean Y–N distances are shorter for **4**. The relatively long Si–C bond to the quaternary C atom in **4** (2.000(3) Å; cf. sum of single bond covalent radii for Si and C = 1.91 Å)^43^ is attributed to steric buttressing of bulky ^*t*^Bu and Cy groups.

**Scheme 2:**
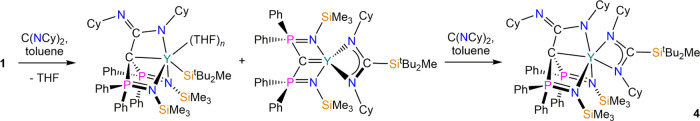
Proposed Stepwise Reaction of Complex **1** with 2 equiv of *N*,*N*′-Dicyclohexyl-Carbodiimide

We repeated the reaction of **1** with 2 equiv of *N*,*N*′-dicyclohexyl-carbodiimide on a larger scale in toluene, and upon work-up of the reaction mixture, we were able to isolate crystals of **4** in sufficient yield (34%) to perform elemental analysis and to collect pristine multinuclear NMR and ATR-IR spectroscopic data. We were able to confirm our previous assignment of the ^29^Si{^1^H} and ^31^P{^1^H} NMR spectra (see above) and assign the ^1^H and ^13^C{^1^H} NMR spectra. The ^13^C{^1^H} NMR spectrum contains characteristic resonances for the silicon-bound (δ_C_: 185.61 ppm) and methanide-bound (δ_C_: 152.53 ppm) quaternary carbon atoms of the C(NCy)_2_ fragments, but the methanide resonance was not observed; this is in contrast to the ^13^C{^1^H} NMR spectrum of [Y{C(PPh_2_NSiMe_3_)_2_[C(NCy)_2_]-κ^4^*C*,*N*,*N*′,*N*′}{C(NCy)_2_(CH_2_Ph)-κ^2^*N*,*N*′}], where the methanide could be assigned (δ_C_: 23.75 ppm, dt, ^1^*J*_YC_ = 3.1 Hz, ^1^*J*_PC_ = 106.6 Hz for the methanide; δ_C_: 153.30, and 175.98 ppm, d, ^2^*J*_YC_ = 2.0 Hz for the C(NCy)_2_ fragments).^41^ Given that the reaction of **1** with 1 equiv of *N*,*N*′-dicyclohexyl-carbodiimide gave resonances in the ^29^Si{^1^H} and ^31^P{^1^H} NMR spectra that were consistent with either 1,2-migratory insertion or [2 + 2]-cycloaddition reactions but not with the isolated product **4**, we propose that the 2:1 reaction proceeds by a divergent route where both reactions can occur to give two different intermediates; addition of a second equivalent of heteroallene leads to the formation of **4** (Scheme 2). This reactivity profile contrasts to the reaction of [Y(BIPM)(CH_2_Ph)(THF)] with *N*,*N*′-dicyclohexyl-carbodiimide, where only the 2:1 reaction product was observed even when only 1 equiv of heteroallene was added; this was attributed to the first reaction activating the intermediate to undergo a rapid second reaction.^41^ This divergence is attributed to differences in Y–C_alkyl_ and Y–Si bonding, which also affects Y=C bonding regimes (see above).

## Conclusions

We have found that bis(iminophosphorano)methanediides are effective supporting ligands for the stabilization of highly polarized-covalent Y–Si bonds by synthesizing three structurally authenticated yttrium silanide complexes using straightforward salt metathesis protocols. ^29^Si{^1^H} NMR spectroscopy showed that variation of the silanide substituents from alkyl to silyl groups modulates the electron density at the silanide center as expected. However, ^13^C{^1^H} NMR spectroscopy indicated that this only results in minor differences in Y=C bonding regimes; the δ_C_ values for the methanediide resonances are all relatively deshielded, and their *J*_YC_ coupling constants are large, compared with similar Y(III) BIPM alkyl complexes that have been reported previously.^25,26^ DFT calculations confirmed the presence of predominantly ionic Y–Si σ-bonds consisting mainly of s/p-hybridized Si orbitals localized at silanide centers, with minor contributions from Y s/d-hybrid orbitals; in all cases, these show lower covalency than the Y=C bonds in the three complexes studied here. The strong σ-donor properties of the silanide ligands was also evident in calculated parameters, where relatively electron-rich Y(III) centers furnish Y=C bonds that are best represented by Y^+^–C^–^ dipolar forms, in common with a Y(III) BIPM silylalkyl complex.^25^ A preliminary reactivity study of one of the Y(III) BIPM silanide complexes reported herein with a selection of unsaturated substrates has shown both complementary and contrasting behaviors to the previously reported reactivity of Y=C and Y–C_alkyl_ bonds in analogous Y(III) BIPM alkyl complexes with the same substrates;^26,27,41^ benzophenone, azobenzene, and *N*,*N*′-dicyclohexyl-carbodiimide are all proposed to undergo 1,2-migratory insertions into the Y–Si bond, and the latter substrate also reacts with the Y=C bond via a [2 + 2]-cycloaddition. Together, the data in this paper have provided a qualitative comparison of Y–Si and Y–C bonds in similar coordination environments.

## Experimental Section

### General Methods and Materials

All syntheses and manipulations were conducted under argon with rigorous exclusion of oxygen and water using Schlenk line and glovebox techniques. Toluene and pentane were sparged with argon and passed through columns containing Q-5 and molecular sieves; these were stored over potassium mirrors and were degassed before use. *d*_6_-Benzene was dried by refluxing over K and was vacuum-transferred and degassed by three freeze–pump–thaw cycles before use. [Y(BIPM)(I)(THF)_2_],^23^ NaSi^*t*^Bu_2_Me,^21,30^ NaSi^*t*^Bu_3_,^21,29^ and K{Si(SiMe_3_)_3_}^28^ were prepared according to literature methods. ^1^H (400.01 MHz), ^13^C{^1^H} (100.61 MHz), ^29^Si{^1^H} (79.48 MHz), and ^31^P{^1^H} (161.95 MHz) NMR spectra (see Supporting Information Figures S1–S21 and S29–S59) were obtained on a Bruker Avance III 400 MHz spectrometer at 298 K. These were referenced to the solvent used or to external SiMe_4_ (^1^H, ^13^C, ^29^Si) or H_3_PO_4_/D_2_O (^31^P). ATR-IR spectra were recorded as microcrystalline powders using a Bruker Tensor 27 spectrometer (see Supporting Information Figures S22–S25 and S60). Elemental analysis (C, H, N) was carried out by either Mr Martin Jennings and Mrs Anne Davies at the Microanalytical Service, Department of Chemistry, the University of Manchester, or the Elemental Analysis Services Team, Science Centre, London Metropolitan University.

### Crystallographic Methods

The crystal data for **1·toluene**, **2·toluene**, **3·0.5pentane**, and **4·1.5*d***_**6**_**-benzene** are compiled in Supporting Information Tables S1 and S2. Crystals of **1·toluene** and **2·toluene** were examined using an Oxford Diffraction Xcalibur diffractometer, equipped with an Agilent Atlas CCD detector with graphite-monochromated Mo Kα (λ = 0.71073 Å) radiation. Crystals of **3·0.5pentane** and **4·1.5*d***_**6**_**-benzene** were examined using a Rigaku FR-X diffractometer, equipped with a HyPix 6000HE photon counting pixel array detector with mirror-monochromated Mo Kα (λ = 0.71073 Å) radiation (**3·0.5pentane**) or Cu Kα (1.54184 Å) radiation (**4·1.5*d***_**6**_**-benzene**). Intensities were integrated from data recorded on 0.75° (**1·toluene**) or 1° (**2·toluene**, **3·0.5pentane**, and **4·1.5*d***_**6**_**-benzene**) frames by ω rotation. Cell parameters were refined from the observed positions of all strong reflections in each data set. A Gaussian grid face-indexed with a beam profile was applied for all structures.^44^ The structures were solved using SHELXT;^45^ the data sets were refined by full-matrix least squares on all unique *F*^2^ values,^45^ with anisotropic displacement parameters for all non-hydrogen atoms and with constrained riding hydrogen geometries; *U*_iso_(H) was set at 1.2 (1.5 for methyl groups) times *U*_eq_ of the parent atom. The largest features in final difference syntheses were close to heavy atoms and were of no chemical significance. CrysAlisPro^44^ was used for control and integration, and SHELX^45,46^ was employed through OLEX2^47^ for structure solution and refinement. ORTEP-3^48^ and POV-Ray^49^ were employed for molecular graphics.

### Computational Methods

Restricted calculations were performed using the Amsterdam Density Functional (ADF) suite version 2017 with standard convergence criteria.^50,51^ Geometry optimizations (see Supporting Information Tables S3–S5) were performed using coordinates derived from the respective crystal structures as the starting points. No constraints were placed on the structures during geometry optimization. The DFT geometry optimizations employed Slater-type orbital TZP polarization all-electron basis sets (from the Dirac and ZORA/TZP database of the ADF suite). Scalar relativistic approaches (spin–orbit neglected) were used within the ZORA Hamiltonian^52−54^ for the inclusion of relativistic effects, and the local density approximation (LDA) with the correlation potential due to Vosko et al. was used in all the calculations.^55^ Generalized gradient approximation corrections were performed using the functionals of Becke and Perdew.^56,57^ NBO analysis was carried out using NBO6.^58^ QTAIM analysis^59,60^ was performed within the ADF package; the MOs and NBOs were visualized using ADFView.^50,51^

### [Y(BIPM)(Si^*t*^Bu_2_Me)(THF)] (**1**)

A Schlenk flask was charged with [Y(BIPM)(I)(THF)_2_] (4.584 g, 5 mmol) and ^*t*^Bu_2_MeSiNa (0.902 g, 5 mmol) and was cooled to −78 °C. Toluene (50 mL) was added, and the resultant beige suspension was allowed to warm to room temperature and stirred for 18 h. The reaction mixture was filtered, and the filtrate was concentrated to 15 mL and stored at −25 °C to give colorless crystals of **1·toluene**, which were isolated and dried under vacuum (2.868 g, 2.97 mmol, 59%). Anal Calcd for C_44_H_67_N_2_OP_2_Si_3_Y: C, 60.39; H, 7.72; N, 3.20. Found: C, 57.98; H, 7.67; N, 3.08. ^1^H NMR (C_6_D_6_, 400 MHz): δ 8.27 (q, 4H, *J*_HH_ = 6.6 Hz, Ar-C*H*), 7.26 (t, 4H, *J*_HH_ = 7.5 Hz, Ar-C*H*), 7.02–7.13 (m, 6H, Ar-C*H*), 6.83 (t, 2H, *J*_HH_ = 7.5 Hz, Ar-C*H*), 6.67 (t, 4H, *J* = 7.5 Hz, Ar-C*H*), 4.00 (m, 4H, OC*H*_2_CH_2_), 1.46 (s, 18H, YSiC(C*H*_3_)_3_), 1.36 (m, 4H, OCH_2_C*H*_2_), 0.43 (s, 3H, YSiC*H*_3_), 0.10 (s, 18H, NSi(C*H*_3_)_3_). ^13^C{^1^H} NMR (C_6_D_6_, 101 MHz): δ 141.21 (t, *J*_PC_ = 56.3 Hz, *ipso*-Ar-*C*), 135.93 (t, *J*_PC_ = 42.9 Hz, *ipso*-Ar-*C*), 132.64 (t, *J*_PC_ = 6.2 Hz, Ar-*C*H), 131.07 (t, *J*_PC_ = 5.9 Hz, Ar-*C*H), 130.11 (Ar-*C*H), 128.64 (Ar-*C*H), 127.34 (t, *J*_PC_ = 5.9 Hz, Ar-*C*H), 71.02 (td, ^1^*J*_PC_ = 128.5 Hz, ^1^*J*_YC_ = 6.6 Hz, Y*C*P_2_), 70.01 (O*C*H_2_CH_2_), 32.60 (SiC(*C*H_3_)_3_), 24.91 (OCH_2_*C*H_2_), 22.48 (Si*C*(CH_3_)_3_), 4.42 (NSi(*C*H_3_)_3_), −0.83 (YSi*C*H_3_). ^29^Si{^1^H} NMR (C_6_D_6_, 79 MHz): δ 12.86 (d, ^1^*J*_YSi_ = 90.4 Hz, Y*Si*^*t*^Bu_2_Me), −8.15 (N*Si*Me_3_). ^31^P{^1^H} NMR (C_6_D_6_, 162 MHz): δ 8.35 (d, ^2^*J*_YP_ = 13.2 Hz, YC*P*_2_). ATR-IR (microcrystalline, cm^–1^) ν̃: 3054 (w), 2945 (m), 2926 (m), 2883 (m), 2856 (m), 2830 (m), 1467 (w), 1434 (m), 1243 (m), 1152 (w), 1099 (m), 1017 (s), 828 (s), 743 (s), 692 (s), 651 (s), 604 (s), 505 (s).

### [Y(BIPM)(Si^*t*^Bu_3_)(THF)] (**2**)

A Schlenk flask was charged with [Y(BIPM)(I)(THF)_2_] (0.917 g, 1 mmol) and ^*t*^Bu_3_SiNa (0.222 g, 1 mmol) and cooled to −78 °C. Toluene (15 mL) was added, and the resultant beige suspension was allowed to warm to room temperature and stirred for 18 h. The reaction mixture was filtered, and the filtrate was concentrated to 5 mL and stored at −25 °C to give colorless crystals of **2·toluene**, which were isolated and dried under vacuum (0.263 g, 0.29 mmol, 29%). Anal Calcd for C_47_H_73_N_2_OP_2_Si_3_Y: C, 61.55; H, 8.02; N, 3.05. Found: C, 59.86; H, 7.68; N, 3.05. ^1^H NMR (C_6_D_6_, 400 MHz): δ 8.29 (q, 4H, *J*_HH_ = 6.7 Hz, Ar-C*H*), 7.27 (t, 4H, *J*_HH_ = 7.4 Hz, Ar-C*H*), 7.18 (t, 2H, *J*_HH_ = 7.4 Hz, Ar-C*H*), 6.92 (q, 4H, *J*_HH_ = 5.9 Hz, Ar-C*H*), 6.79 (t, 2H, *J*_HH_ = 7.4 Hz, Ar-C*H*), 6.61 (t, 4H, *J*_HH_ = 7.4 Hz, Ar-C*H*), 4.13 (m, 4H, OC*H*_2_CH_2_), 1.53 (s, 27H, SiC(C*H*_3_)_3_), 1.40 (m, 4H, OCH_2_C*H*_2_), 0.12 (s, 18H, NSi(C*H*_3_)_3_). ^13^C{^1^H} NMR (C_6_D_6_, 101 MHz): δ 140.85 (t, *J*_PC_ = 57.4 Hz, *ipso*-Ar-*C*), 135.44 (t, *J*_PC_ = 41.3 Hz, *ipso*-Ar-*C*), 133.17 (t, *J*_PC_ = 6.1 Hz, Ar-*C*H), 131.38 (t, *J*_PC_ = 5.7 Hz, Ar-*C*H), 130.11 (Ar-*C*H), 128.72 (Ar-*C*H), 127.62 (t, *J*_PC_ = 6.1 Hz, Ar-*C*H), 127.24 (t, *J*_PC_ = 5.7 Hz, Ar-*C*H), 72.81 (td, ^1^*J*_PC_ = 120.1 Hz, ^1^*J*_YC_ = 7.4 Hz, Y*C*P_2_), 70.66 (O*C*H_2_CH_2_), 34.95 (SiC(*C*H_3_)_3_), 25.30 (OCH_2_*C*H_2_), 25.08 (Si*C*(CH_3_)_3_), 4.96 (NSi(*C*H_3_)_3_). ^29^Si{^1^H} NMR (C_6_D_6_, 79 MHz): δ 33.53 (d, ^1^*J*_YSi_ = 88.9 Hz, Y*Si*^*t*^Bu_3_), −8.13 (N*Si*Me_3_). ^31^P{^1^H} NMR (C_6_D_6_, 162 MHz): δ 9.41 (d, ^2^*J*_YP_ = 13.8 Hz, YC*P*_2_). ATR-IR (microcrystalline, cm^–1^) ν̃: 3051 (w), 2947 (m), 2889 (m), 2860 (m), 2842 (m), 1479 (w), 1434 (m), 1261 (s), 1241 (s), 1171 (w), 1101 (m), 1017 (s), 826 (s), 801 (s), 739 (s), 692 (s), 649 (s), 604 (s), 550 (s), 502 (s), 462 (s).

### [Y(BIPM){Si(SiMe_3_)_3_}(THF)] (**3**)

A Schlenk flask was charged with [Y(BIPM)(I)(THF)_2_] (0.917 g, 1 mmol) and K{Si(SiMe_3_)_3_} (0.287 g, 1 mmol) and cooled to −78 °C. Toluene (25 mL) was added, and the resultant beige suspension was allowed to warm to room temperature and stirred for 24 h. Volatiles were removed in vacuo, and the product was extracted into pentane (15 mL). Filtration and storage at −25 °C overnight gave colorless crystals of **3·(pentane)**_**0.5**_, which were isolated and dried under vacuum (0.536 g, 0.56 mmol, 56%). Anal Calcd for C_44_H_73_N_2_OP_2_Si_6_Y: C, 54.74; H, 7.62; N, 2.90. Found: C, 55.01; H, 7.74; N, 2.76. ^1^H NMR (C_6_D_6_, 400 MHz): δ 8.23 (q, 4H, *J*_HH_ = 6.8 Hz, Ar-C*H*), 7.28 (t, 4H, *J*_HH_ = 7.4 Hz, Ar-C*H*), 7.18 (t, 2H, *J*_HH_ = 7.4 Hz, Ar-C*H*), 6.96 (q, 4H, *J*_HH_ = 5.9 Hz, Ar-C*H*), 6.81 (t, 2H, *J*_HH_ = 7.3 Hz, Ar-C*H*), 6.64 (t, 4H, *J*_HH_ = 7.3 Hz, Ar-C*H*), 4.17 (m, 4H, OC*H*_2_CH_2_), 1.41 (m, 4H, OCH_2_C*H*_2_), 0.58 (s, 27H, Si{Si(C*H*_3_)_3_}_3_), 0.13 (s, 18H, NSi(C*H*_3_)_3_). ^13^C{^1^H} NMR (C_6_D_6_, 101 MHz): δ 140.82 (t, *J*_PC_ = 57.8 Hz, *ipso*-Ar-*C*), 135.45 (t, *J*_PC_ = 43.0 Hz, *ipso*-Ar-*C*), 133.04 (t, *J*_PC_ = 6.1 Hz, Ar-*C*H), 131.29 (t, *J*_PC_ = 5.8 Hz, Ar-*C*H), 130.21 (s, Ar-*C*H), 128.85 (s, Ar-*C*H), 127.74 (t, *J*_PC_ = 6.1 Hz, Ar-*C*H), 127.35 (t, *J*_PC_ = 5.8 Hz, Ar-*C*H), 72.45 (td, ^1^*J*_PC_ = 120.4 Hz, ^1^*J*_YC_ = 7.5 Hz, Y*C*P_2_), 70.48 (O*C*H_2_CH_2_), 24.89 (OCH_2_*C*H_2_), 6.92 (Si{Si(*C*H_3_)_3_}_3_), 5.08 (NSi(*C*H_3_)_3_). ^29^Si{^1^H} NMR (C_6_D_6_, 79 MHz): δ −6.22 (d, ^2^*J*_YSi_ = 1.0 Hz, Si{*Si*(CH_3_)_3_}_3_), −8.10 (N*Si*(CH_3_)_3_), −148.54 (d, ^1^*J*_YSi_ = 67.9 Hz, *Si*{Si(CH_3_)_3_}_3_). ^31^P{^1^H} NMR (C_6_D_6_, 162 MHz): δ 9.25 (d, ^2^*J*_YP_ = 13.7 Hz, YC*P*_2_). ATR-IR (microcrystalline, cm^–1^) ν̃: 3051 (w), 2947 (w), 2889 (w), 1434 (w), 1266 (m), 1239 (m), 1175 (w), 1103 (w), 1068 (w), 1025 (w), 996 (w), 859 (m), 826 (m), 799 (m), 739 (m), 694 (m), 633 (m), 509 (m), 460 (m).

### Reaction of **1** with Benzophenone

*d*_6_-Benzene (0.6 mL) was added to a Youngs tap-appended NMR tube charged with **1** (0.050 g, 0.052 mmol) and Ph_2_CO (0.010 g, 0.052 mmol) at room temperature. The dark-red reaction mixture turned yellow within 1 h. After 8 h, ^1^H, ^13^C{^1^H}, ^29^Si{^1^H}, and ^31^P{^1^H} NMR spectra were collected. A further equivalent of Ph_2_CO (0.010 g, 0.052 mmol) was added at room temperature, and after 8 h, the NMR analysis was repeated.

### Reaction of **1** with Azobenzene

*d*_6_-Benzene (0.6 mL) was added to a Youngs tap-appended NMR tube charged with **1** (0.050 g, 0.052 mmol) and PhN=NPh (0.010 g, 0.052 mmol) at room temperature, giving a dark-green reaction mixture. After 8 h, ^1^H, ^13^C{^1^H}, ^29^Si{^1^H}, and ^31^P{^1^H} NMR spectra were collected. A further equivalent of PhN=NPh (0.010 g, 0.052 mmol) was added at room temperature, and after 8 h, the NMR analysis was repeated.

### Reaction of **1** with *N*,*N*′-Dicyclohexyl-carbodiimide

***Method 1.****d*_6_-Benzene (0.6 mL) was added to a Youngs tap-appended NMR tube charged with **1** (0.050 g, 0.052 mmol) and CyN=C=NCy (0.011 g, 0.052 mmol) at room temperature, giving a pale-yellow reaction mixture. After 8 h, ^1^H, ^13^C{^1^H}, ^29^Si{^1^H}, and ^31^P{^1^H} NMR spectra were collected. A further equivalent of CyN=C=NCy (0.011 g, 0.052 mmol) was added at room temperature, and crystals of **4·1.5*d***_**6**_**-benzene** rapidly formed; these were identified by single-crystal XRD. After 8 h, the NMR analysis was repeated. ***Method 2.*** Toluene (5 mL) was added to a Schlenk flask charged with **1** (0.250 g, 0.258 mmol) and CyN=C=NCy (0.107 g, 0.517 mmol). The resultant pale-yellow reaction mixture was stirred at room temperature for 18 h. Volatiles were removed in vacuo to obtain an off-white solid, which was redissolved in toluene (1 mL). Storage at −30 °C gave colorless crystals. The isolated product was identified as **4** by NMR spectroscopy (0.106 g, 0.09 mmol, 34%). Anal Calcd for C_73_H_112_N_6_P_2_Si_3_Y (**4·toluene**): C, 66.99; H, 8.63; N, 6.42. Found: C, 66.49; H, 8.61; N, 6.23. ^1^H NMR (C_6_D_6_, 400 MHz): δ 8.12 (br m, 4H, Ar-C*H*), 7.67 (br m, 4H, Ar-C*H*), 7.11 (m, 4H, Ar-C*H*), 7.02 (t, 4H, *J*_HH_ = 7.4 Hz, Ar-C*H*), 6.83 (m, 2H, Ar-C*H*), 6.78 (m, 2H, Ar-C*H*), 4.38 (br m, 1H, C*H*_2_-1 NCy), 3.59 (br m, 2H, C*H*_2_-1 NCy), 3.35 (br m, 1H, C*H*_2_-1 NCy), 2.45 (br m, 2H, C*H*_2_ NCy), 2.37 (br m, 2H, C*H*_2_ NCy), 2.28 (br m, 2H, C*H*_2_ NCy), 2.21 (br m, 2H, C*H*_2_ NCy), 1.90 (br m, 8H, C*H*_2_ NCy), 1.73 (br m, 8H, C*H*_2_ NCy), 1.51 (br m, 8H, C*H*_2_ NCy), 1.29 (s, 18H, SiC(C*H*_3_)_3_), 1.08 (br m, 2H, C*H*_2_ NCy), 0.72 (s, 3H, SiC*H*_3_), 0.68 (br m, 2H, C*H*_2_ NCy), 0.54 (br m, 4H, C*H*_2_ NCy), 0.07 (s, 18H, NSi(C*H*_3_)_3_). ^13^C{^1^H} NMR (C_6_D_6_, 101 MHz): δ 186.01 (Si*C*N_2_), 152.91 (C*C*N_2_), 137.89 (m, *ipso*-Ar-*C*) 134.59 (t, *J*_PC_ = 5.0 Hz, Ar-*C*H), 133.55 (br m, Ar-*C*H), 130.82 (Ar-*C*H), 129.33 (Ar-*C*H), 128.57 (Ar-*C*H), 127.54 (br m, Ar-*C*H) 125.70 (Ar-*C*H), 59.85 (*C*-1 NCy), 59.76 (*C*-1 NCy), 57.30 (*C*-1 NCy), 56.69 (*C*-1 NCy), 38.37 (*C*H_2_-2 NCy), 38.25 (*C*H_2_-2 NCy), 35.40 (*C*H_2_-2 NCy), 34.69 (*C*H_2_-2 NCy), 31.25 (SiC(*C*H_3_)_3_), 31.14 (*C*H_2_-3 NCy), 28.01 (*C*H_2_-3 NCy), 27.59 (*C*H_2_-3 NCy), 26.96 (*C*H_2_-3 NCy), 25.97 (*C*H_2_-4 NCy), 25.85 (*C*H_2_-4 NCy), 25.68 (*C*H_2_-4 NCy), 25.08 (*C*H_2_-4 NCy), 22.51 (Si*C*(CH_3_)_3_), 4.40 (NSi(*C*H_3_)_3_), 0.60 (Si*C*H_3_). Y*C*P_2_ not observed. ^29^Si{^1^H} NMR (C_6_D_6_, 79 MHz): δ 7.07 (*Si*^*t*^Bu_2_Me), −5.95 (N*Si*Me_3_). ^31^P{^1^H} NMR (C_6_D_6_, 162 MHz): δ 18.36 (d, ^2^*J*_YP_ = 4.9 Hz, YC*P*_2_). ATR-IR (microcrystalline, cm^–1^) ν̃: 3054 (w), 2920 (m), 2850 (m), 1531 (m), 1467 (w), 1436 (m), 1356 (w), 1329 (m), 1249 (m), 1107 (m), 1062 (m), 1023 (m), 832 (m), 692 (m), 608 (m), 522 (m), 482 (m).

## Supporting Information Available

Additional experimental details for physical and computational data associated with this article (PDF)

## Notes

Additional research data supporting this publication are available from Figshare at https://figshare.com/doi/10.48420/21539316.

## Dedication

Dedicated to Professor William J. Evans on the occasion of his 75^th^ birthday.
