# Supplementary material for: Synthesis and Characterization of Yttrium Methanediide Silanide Complexes
Source: Inorg Chem. 2022 Dec 20;62(1):137–46. doi: 10.1021/acs.inorgchem.2c03053 (PMC9832533; doi:10.1021/acs.inorgchem.2c03053)
Supplement: Supplementary file 1 — ic2c03053_si_001.pdf [file ic2c03053_si_001.pdf]

- Supporting Information -

Synthesis and Characterization of Yttrium Methanediide Silanide Complexes

Benjamin L. L. Réant, Ashley J. Wooles, Stephen T. Liddle, and David P. Mills**

Department of Chemistry, The University of Manchester, Oxford Road,

Manchester, M13 9PL, U.K.

*Email: david.mills@manchester.ac.uk; steve.liddle@manchester.ac.uk

Contents

1. NMR spectroscopy S2
1.1. ^{1}H NMR spectra of 1-3 .... S2
1.2. ^{29}Si{^{1}H} NMR spectra of 1-3 .... S3
1.3. ^{31}P{^{1}H} NMR spectra of 1-3 .... S5
1.4. ^{13}C{^{1}H} NMR spectra of 1-3 .... S6
1.5. 2D NMR spectra of 1-3 .... S9
2. ATR-IR spectroscopy S14
3. Crystallographic data S16
4. NBO representations of selected frontier orbitals of 1-3 .... S18
5. Optimized geometry coordinates for 1-3 .... S19
6. NMR spectra of reactivity studies of 1 .... S28
6.1. Benzophenone (1 equivalent) S28
6.2. Benzophenone (2 equivalents) S29
6.3. Azobenzene (1 equivalent) S30
6.4. Azobenzene (2 equivalents) S32
6.5. *N,N′*-dicyclohexyl-carbodiimide (1 equivalent) S33
6.6. *N,N′*-dicyclohexyl-carbodiimide (2 equivalents) S34
7. Characterization data for 4 .... S36
7.1. NMR spectra of 4 .... S36
7.2. ATR-IR spectrum of 4 .... S39

1. NMR spectroscopy

1.1. ¹H NMR spectra of 1-3

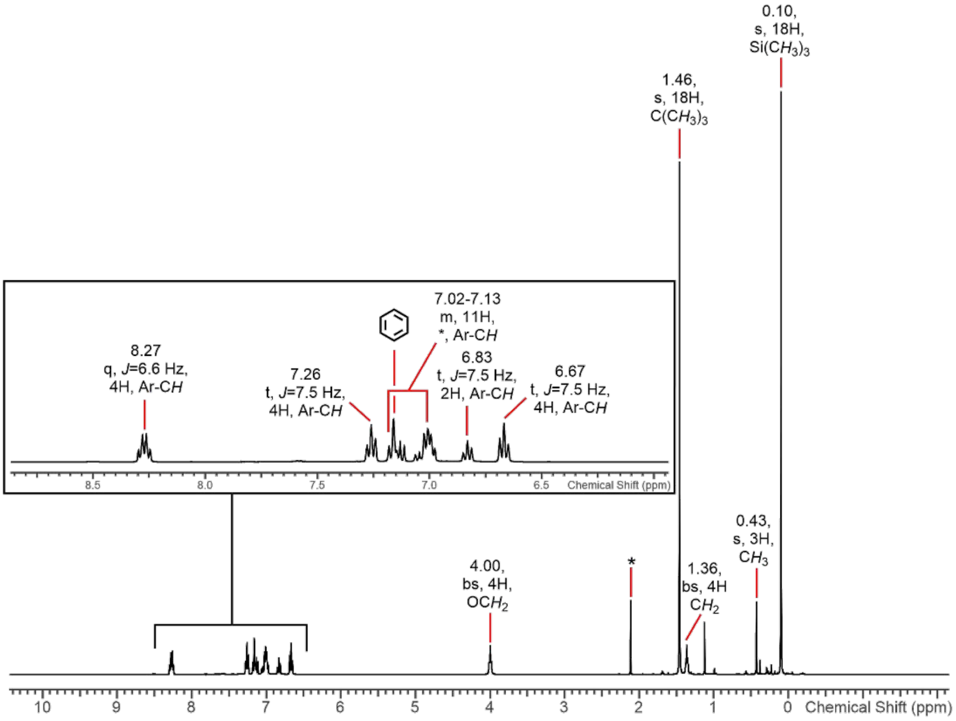

Figure S1. ¹H NMR spectrum of **1** in d_6-benzene. *denotes residual toluene.

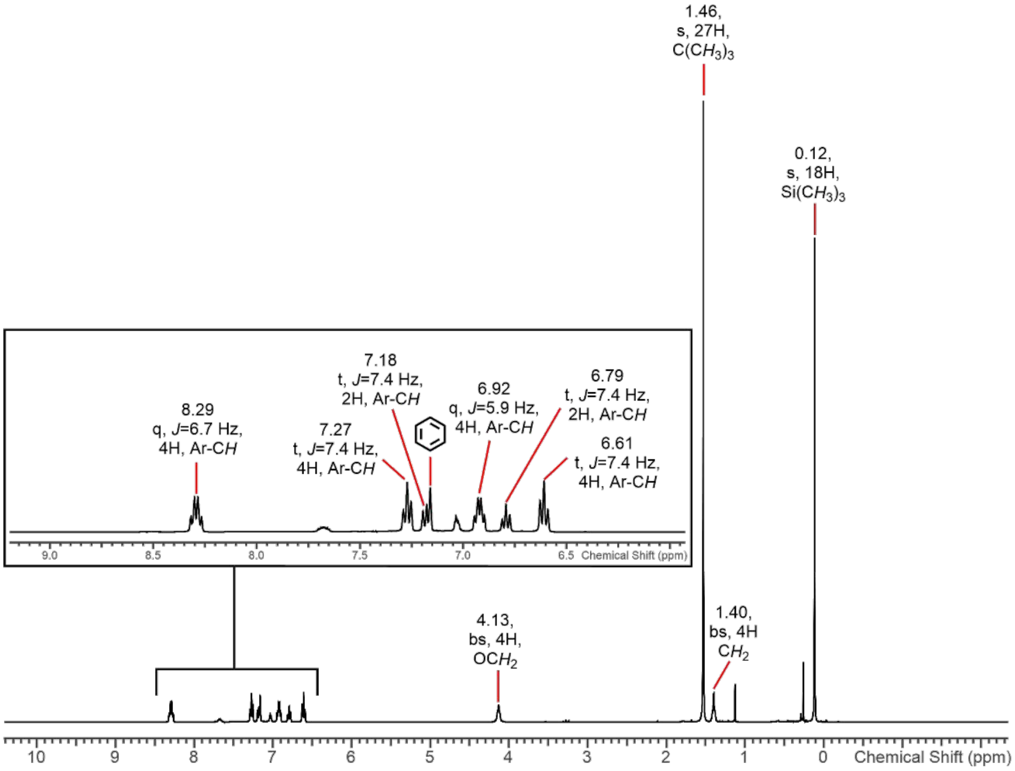

Figure S2. ¹H NMR spectrum of **2** in d_6-benzene.

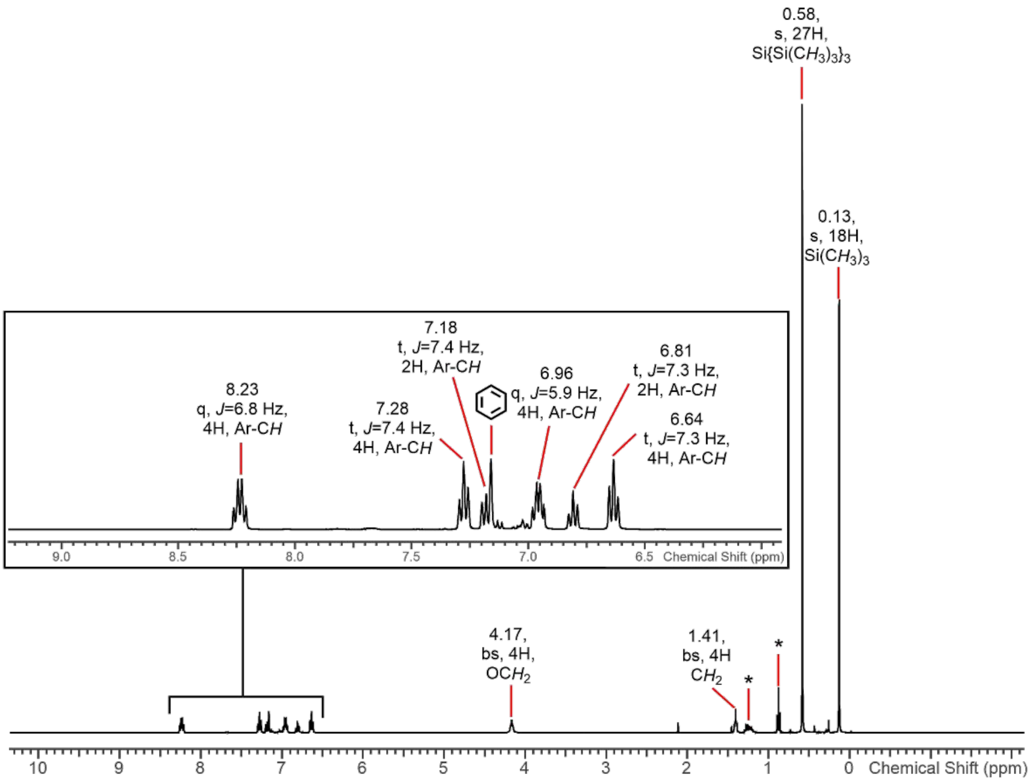

Figure S3. ^{1}H NMR spectrum of **3** in d_6-benzene. *denotes residual pentane.

1.2. $^{29}Si\{^{1}H\}$ NMR spectra of 1-3

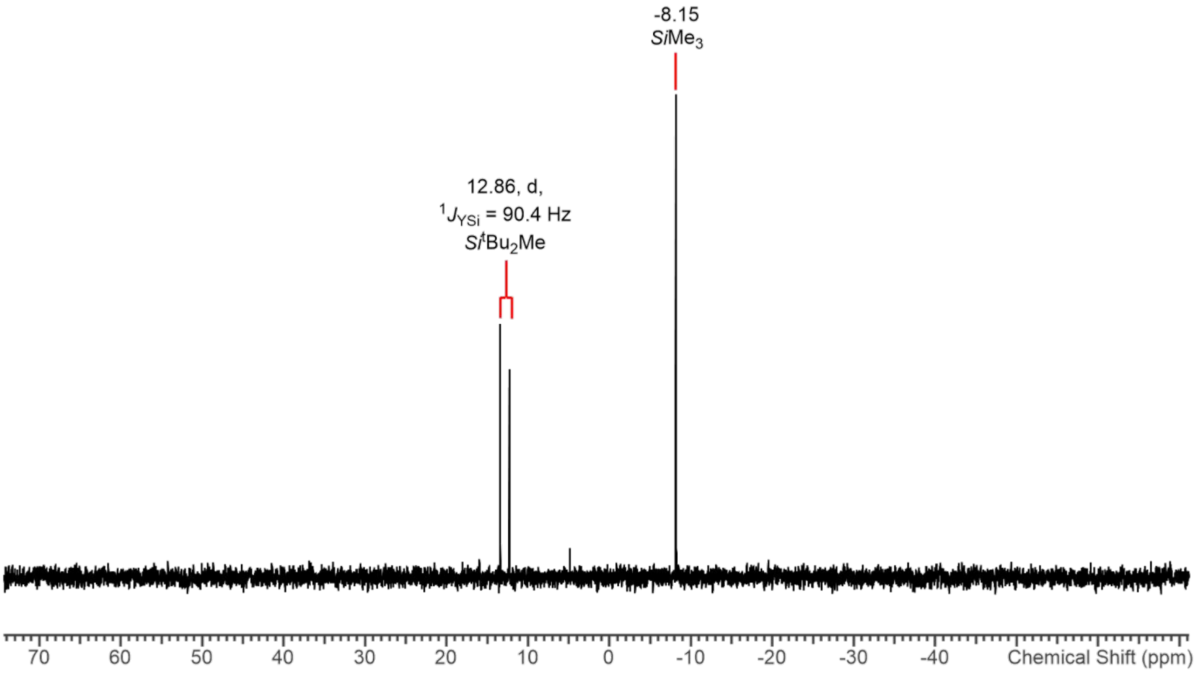

Figure S4. $^{29}Si\{^{1}H\}$ NMR spectrum of **1** in d_6-benzene.

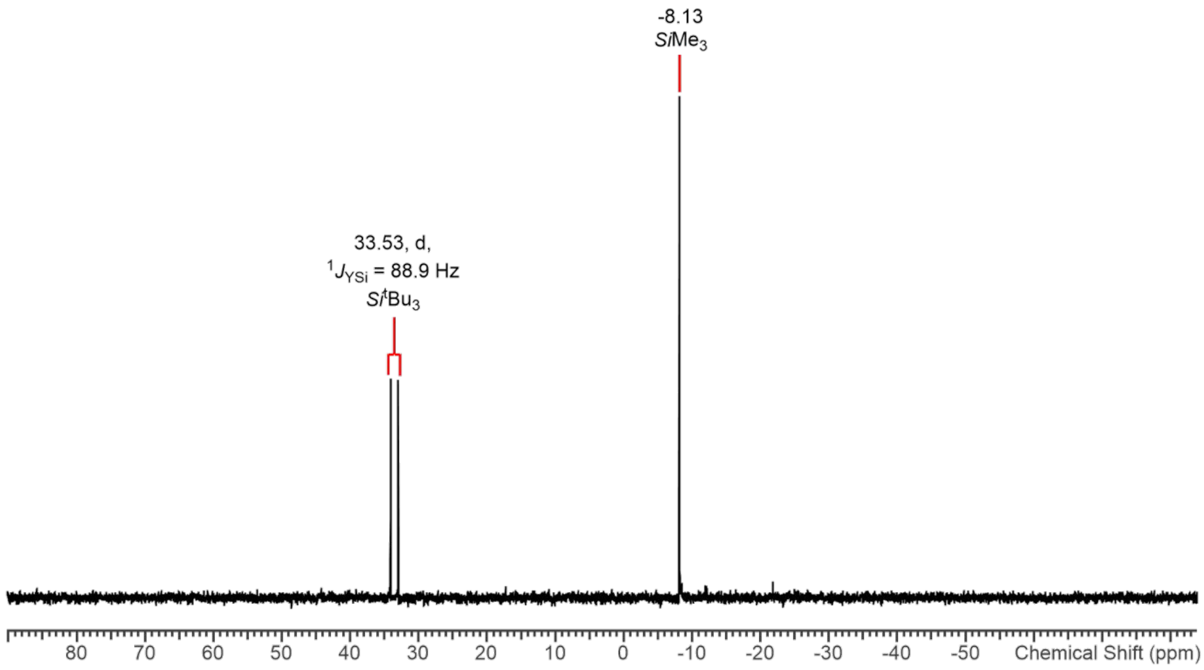

Figure S5. $^{29}Si\{^1H\}$ NMR spectrum of **2** in d_6-benzene.

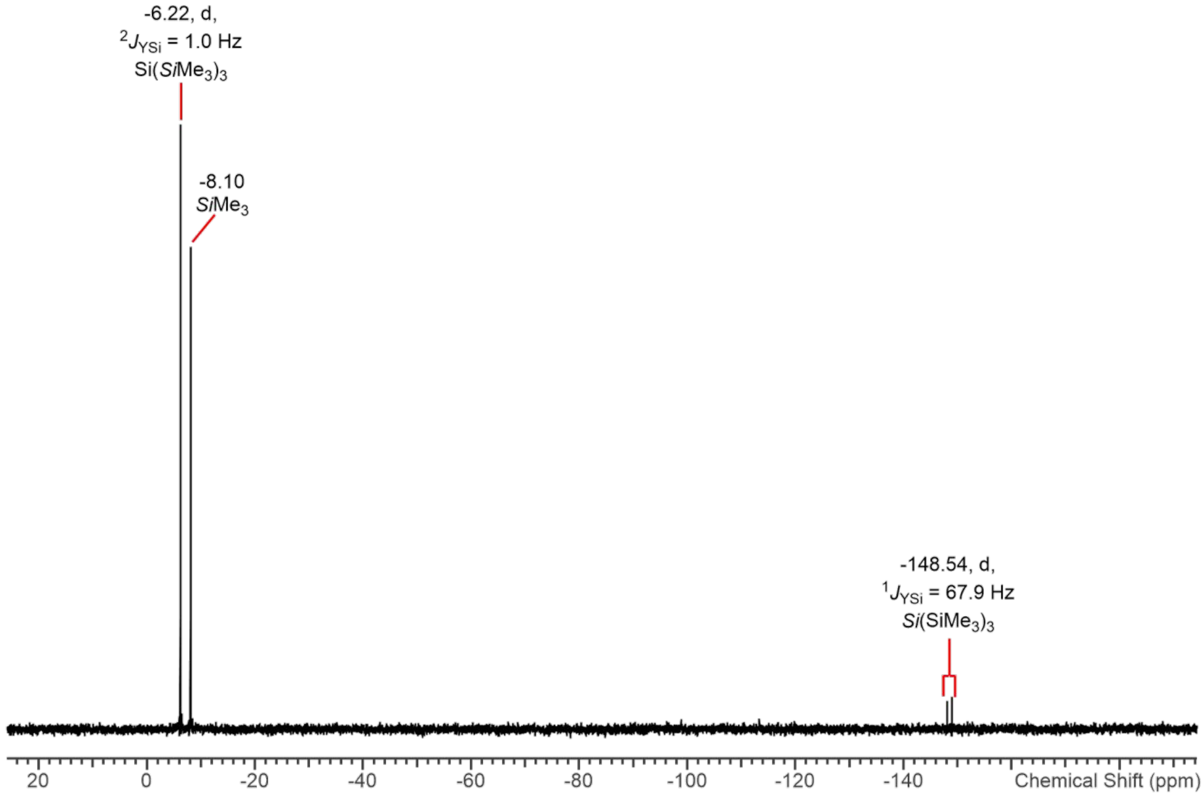

Figure S6. $^{29}Si\{^1H\}$ NMR spectrum of **3** in d_6-benzene.

1.3. $^{31}P\{^1H\}$ NMR spectra of 1-3

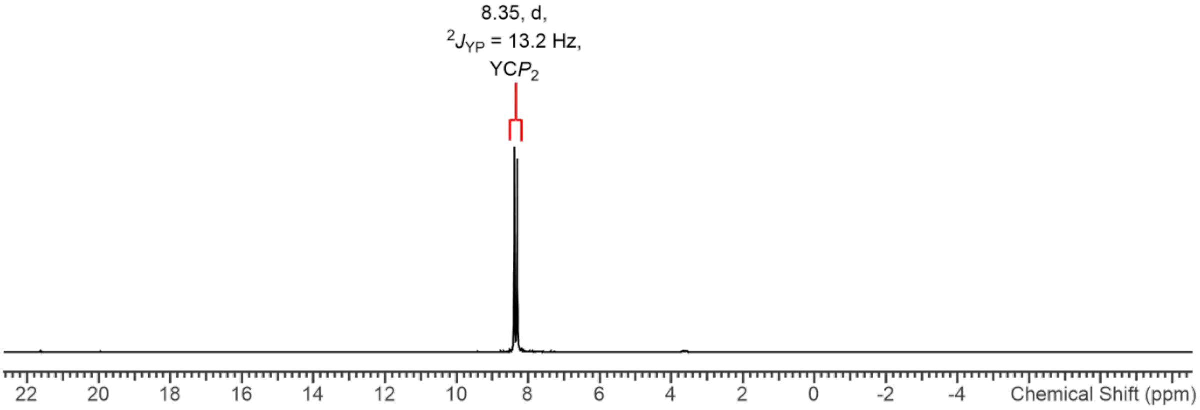

Figure S7. $^{31}P\{^1H\}$ NMR spectrum of **1** in d_6-benzene.

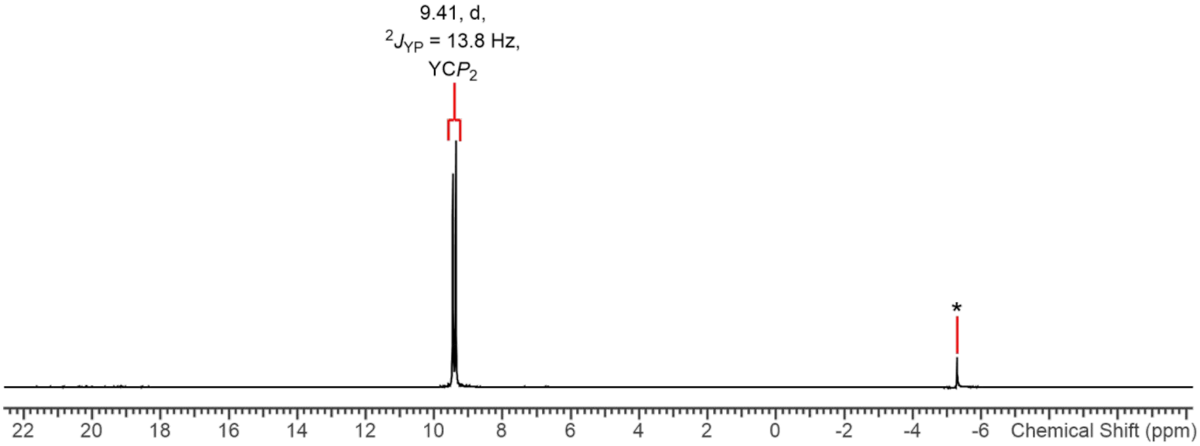

Figure S8. $^{31}P\{^1H\}$ NMR spectrum of **2** in d_6-benzene. *denotes minor BIPMH$_2$ impurity.

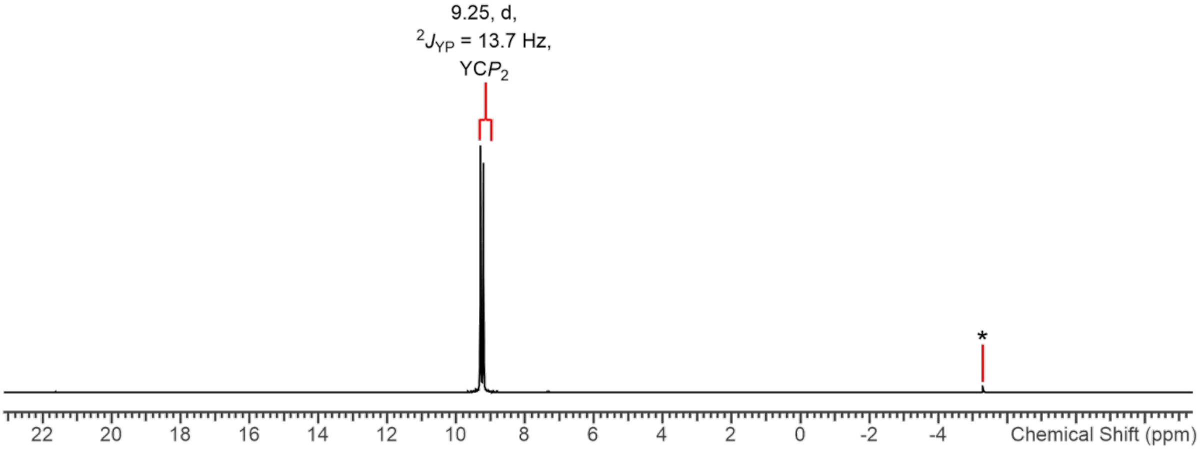

Figure S9. $^{31}P\{^1H\}$ NMR spectrum of **3** in d_6-benzene. *denotes minor BIPMH$_2$ impurity.

1.4. $^{13}C\{^1H\}$ NMR spectra of 1-3

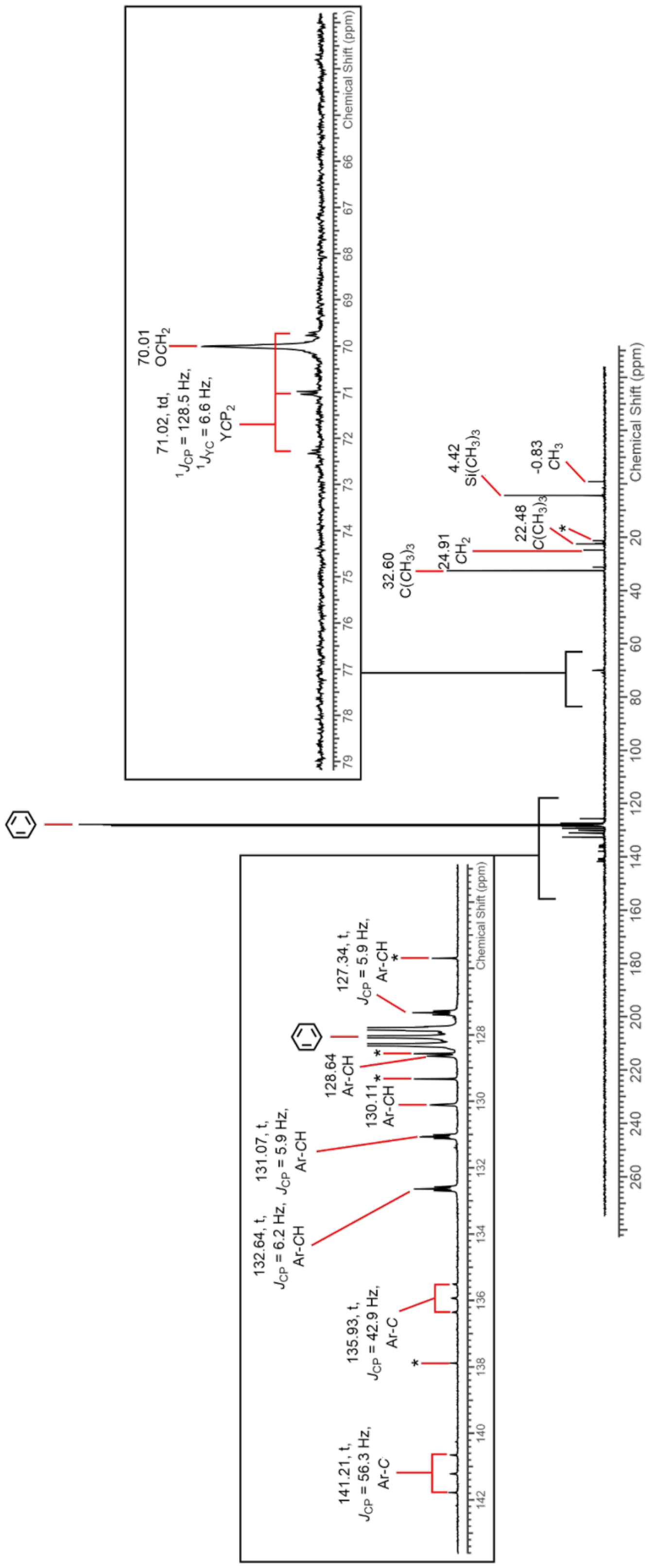

Figure S10. $^{13}C\{^1H\}$ NMR spectrum of **1** in d_6-benzene. * denotes residual toluene.

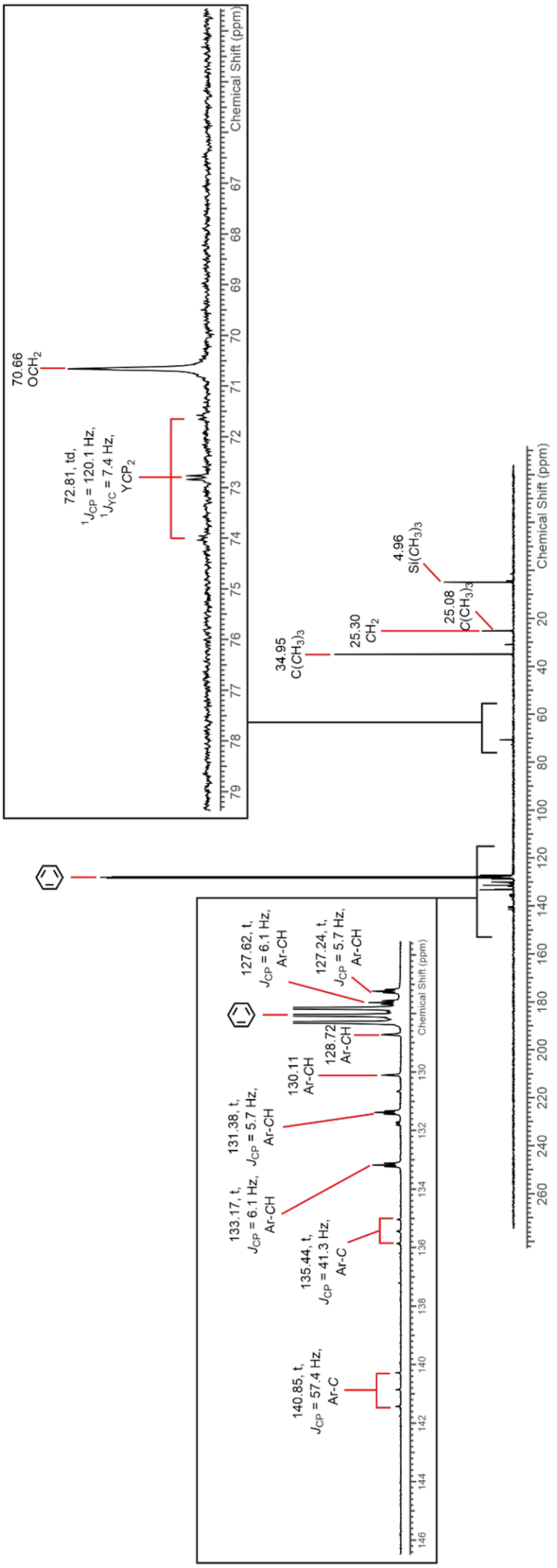

Figure S11. $^{13}C\{^{1}H\}$ NMR spectrum of **2** in d_6-benzene.

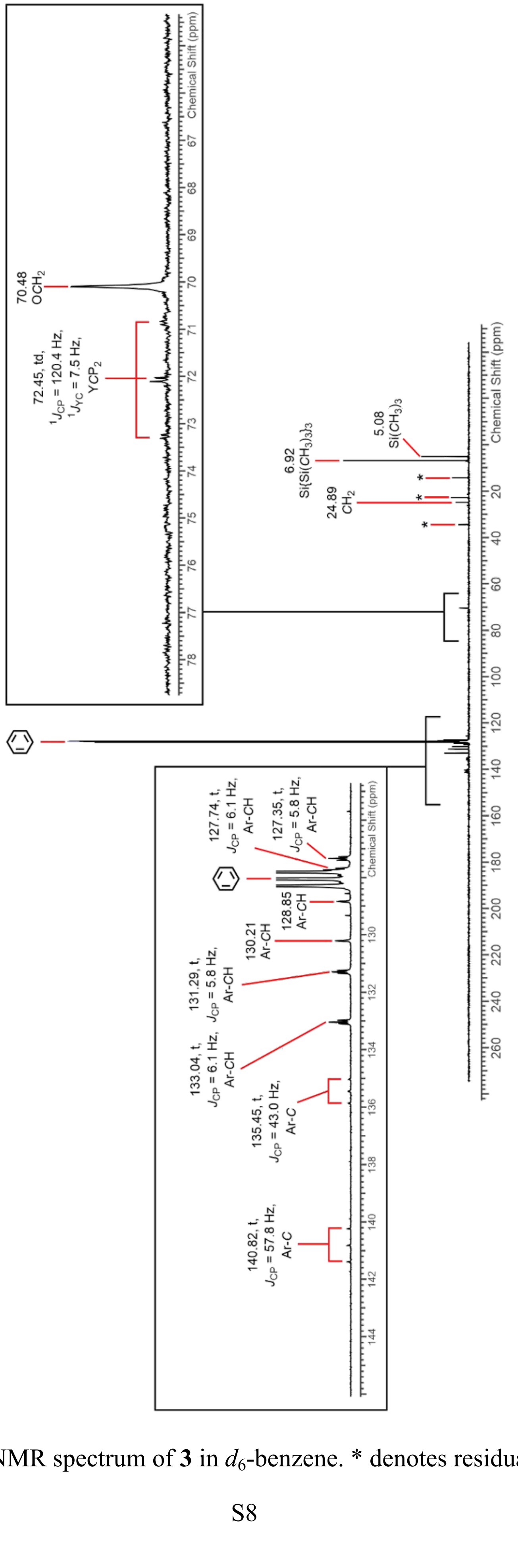

Figure S12. $^{13}C\{^1H\}$ NMR spectrum of **3** in d_6-benzene. * denotes residual pentane.

1.5. 2D NMR spectra of 1-3

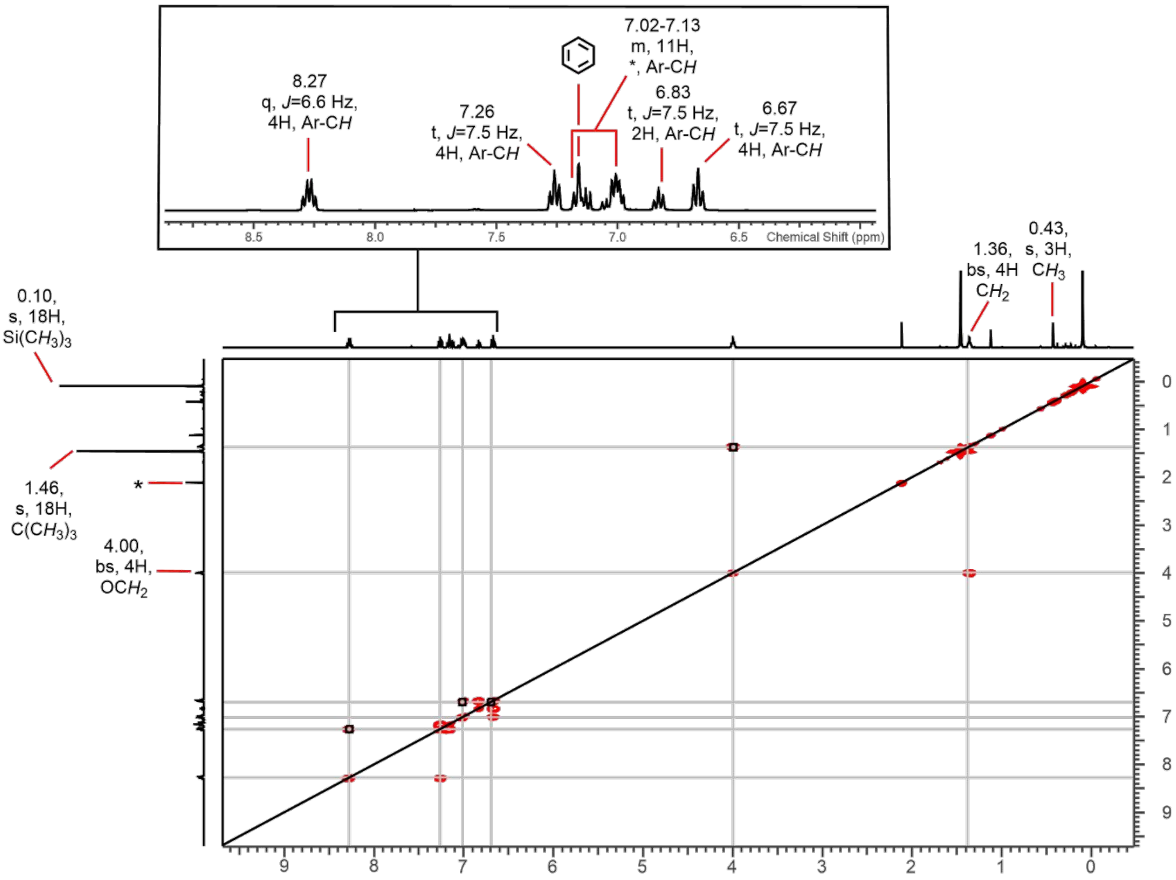

Figure S13. COSY NMR spectrum of **1** in d_6-benzene. * denotes residual toluene.

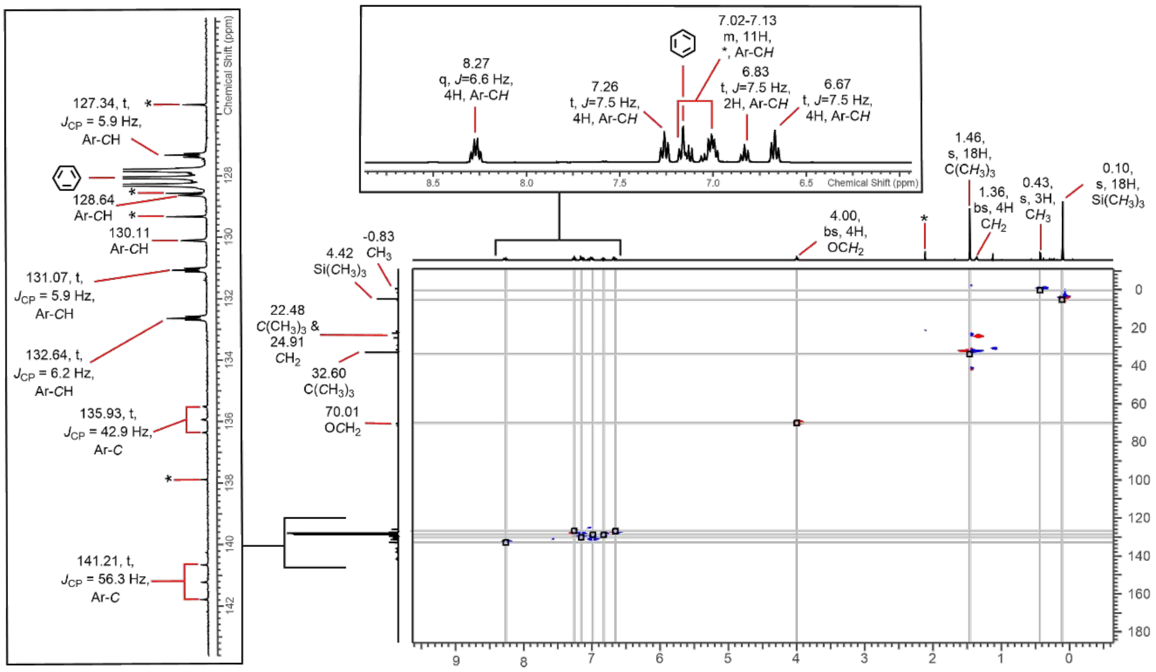

Figure S14. ^{1}H–^{13}C HSQC NMR spectrum of **1** in d_6-benzene. * denotes residual toluene.

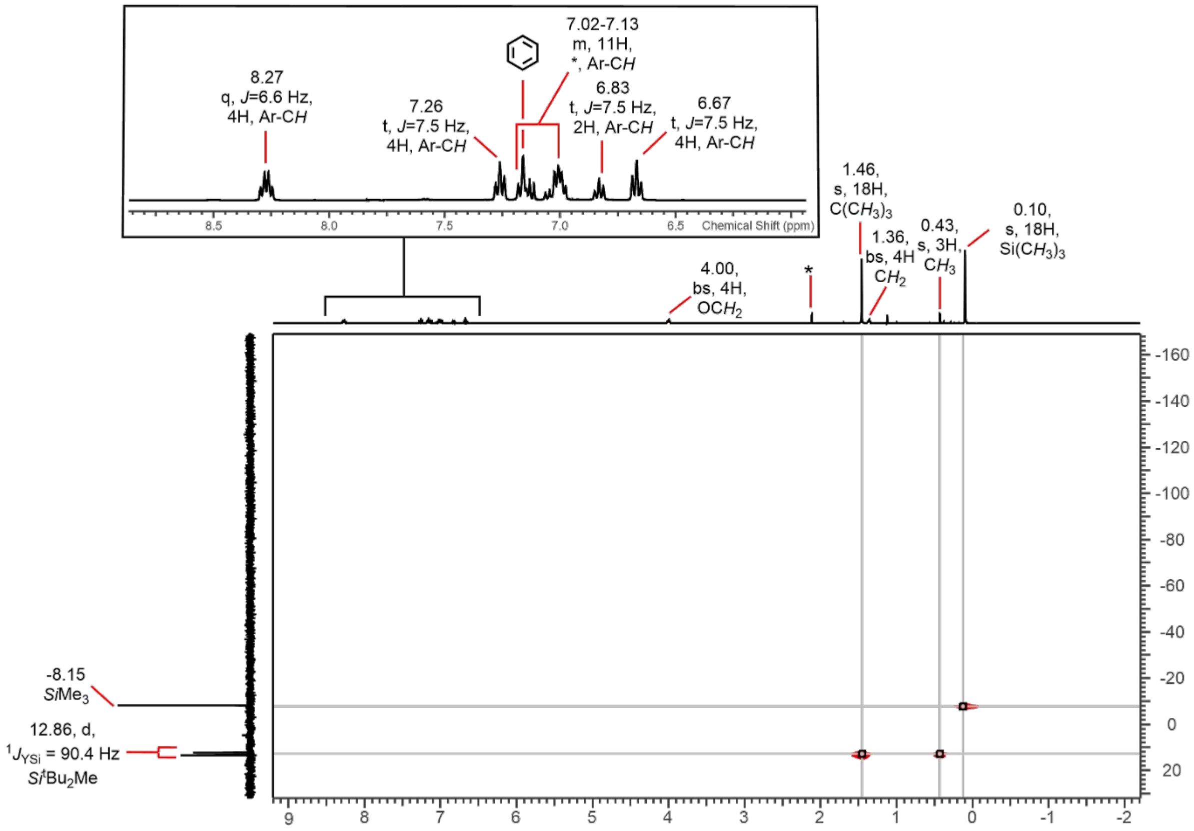

Figure S15. ^{1}H–^{29}Si HMBC NMR spectrum of **1** in d_6-benzene. * denotes residual toluene.

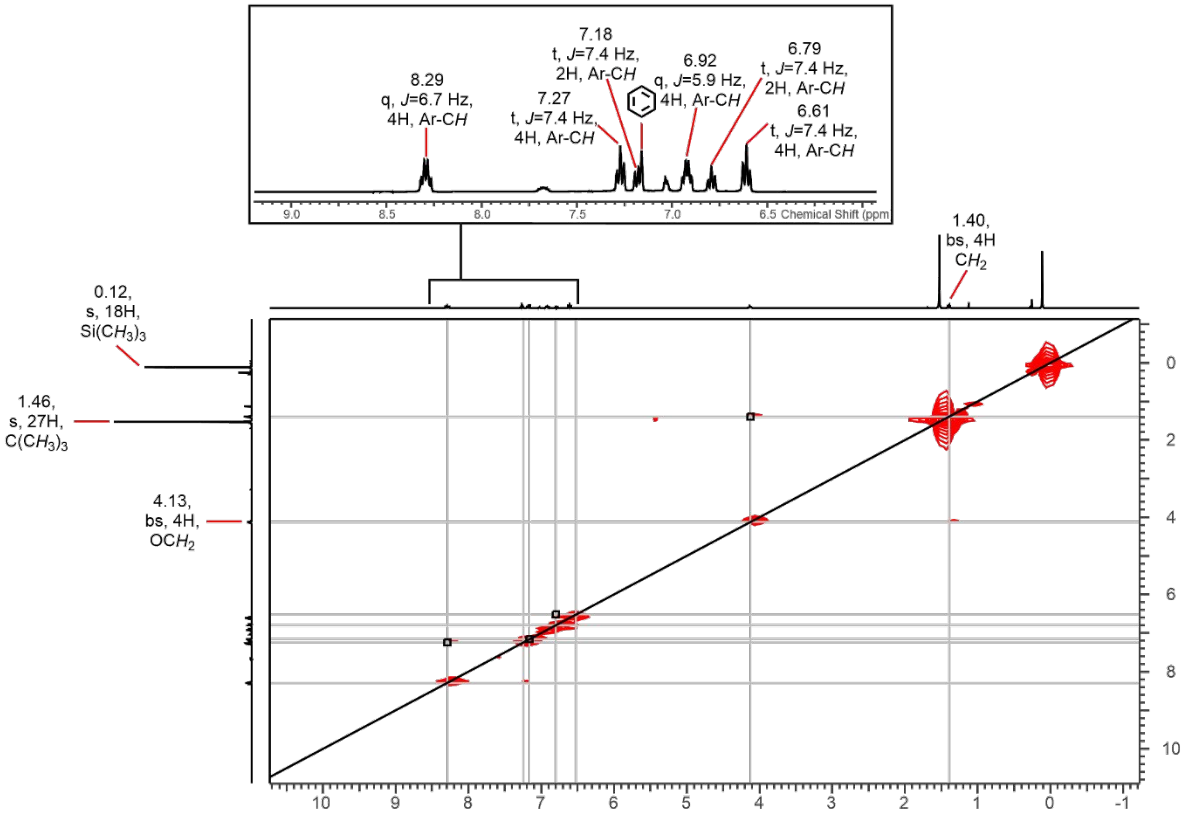

Figure S16. COSY NMR spectrum of **2** in d_6-benzene.

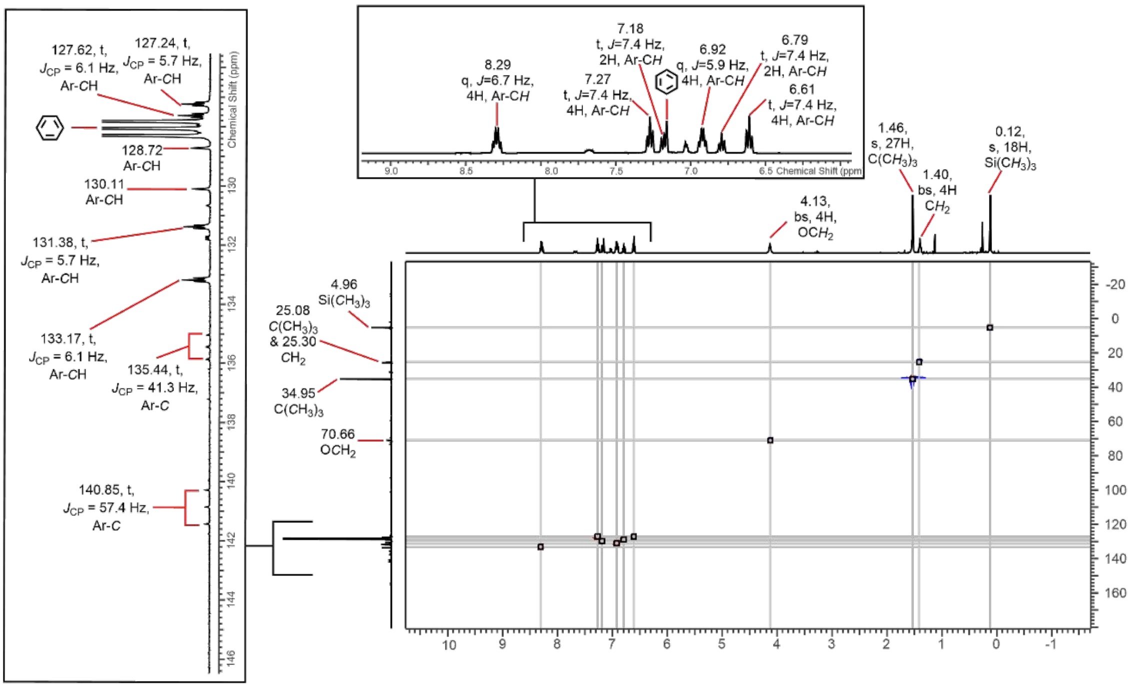

Figure S17. ^{1}H–^{13}C HSQC NMR spectrum of **2** in d_6-benzene.

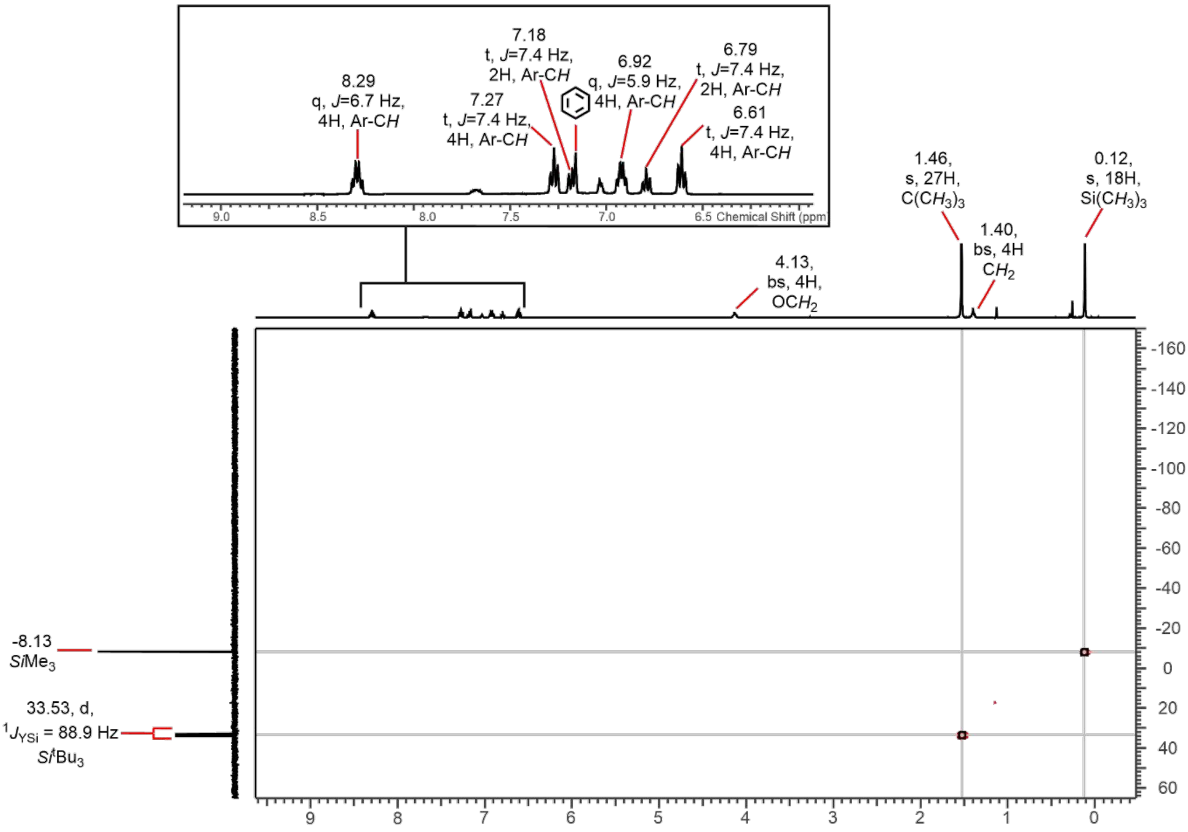

Figure S18. ^{1}H–^{29}Si HMBC NMR spectrum of **2** in d_6-benzene.

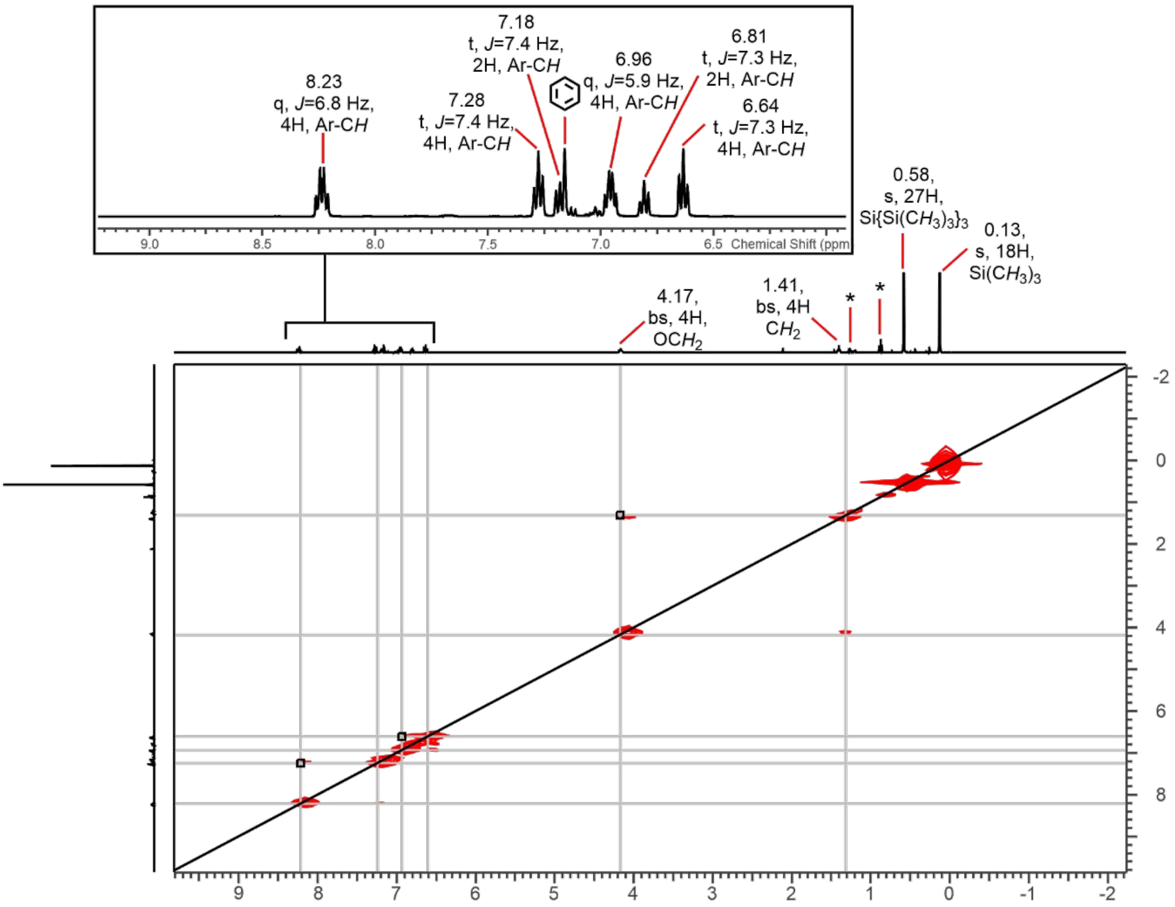

Figure S19. COSY NMR spectrum of **3** in d_6-benzene. * denotes residual pentane.

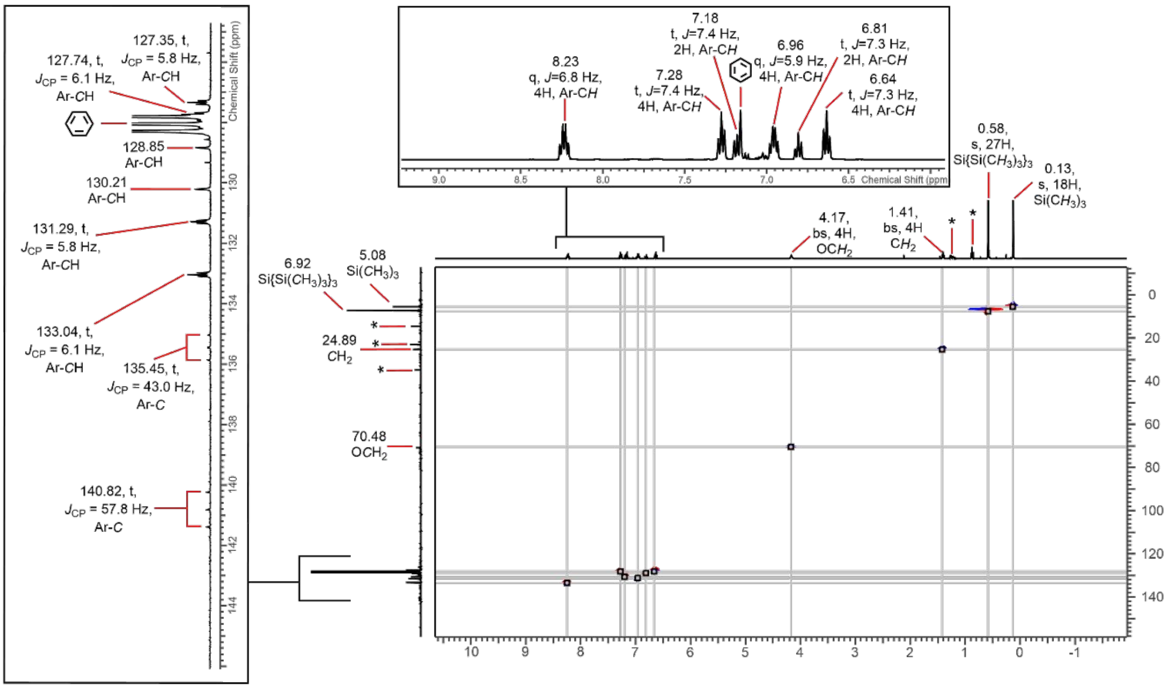

Figure S20. ^{1}H–^{13}C HSQC NMR spectrum of **3** in d_6-benzene. * denotes residual pentane.

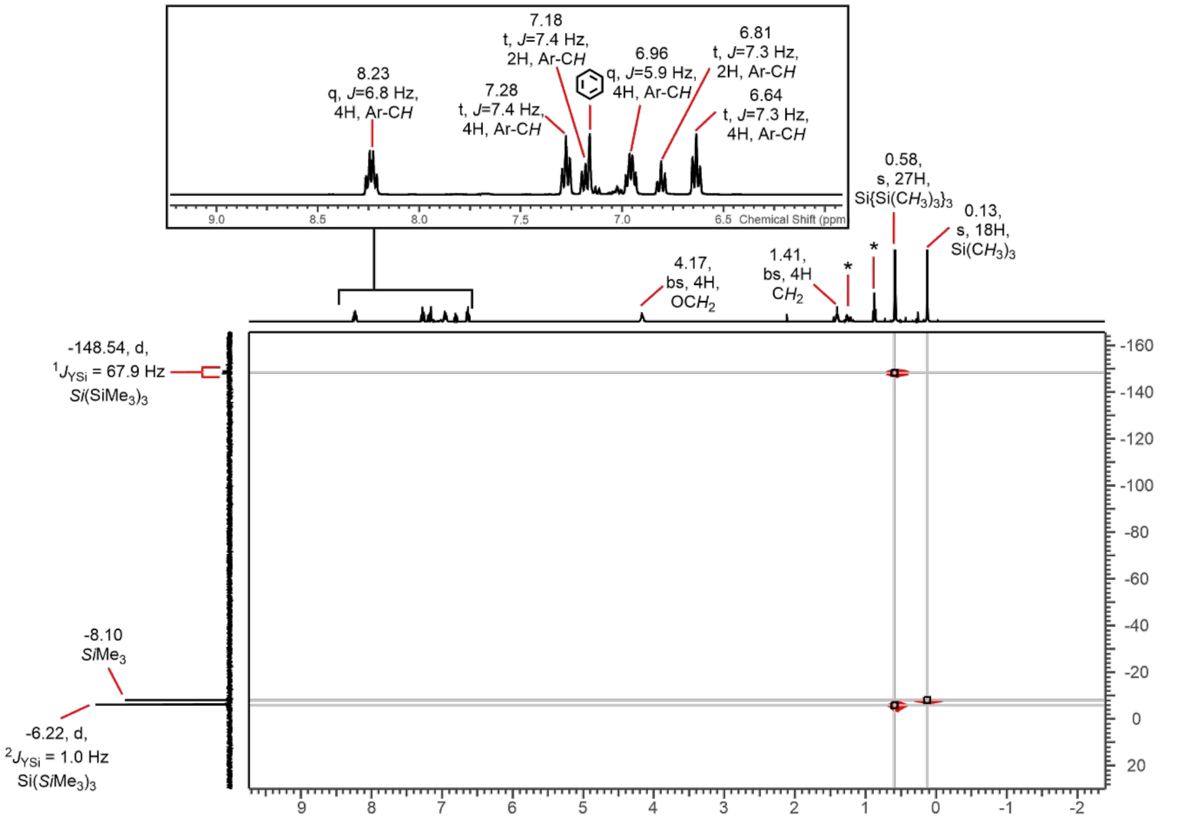

Figure S21. ^{1}H–^{29}Si HMBC NMR spectrum of **3** in d_6-benzene. * denotes residual pentane.

2. ATR-IR spectroscopy

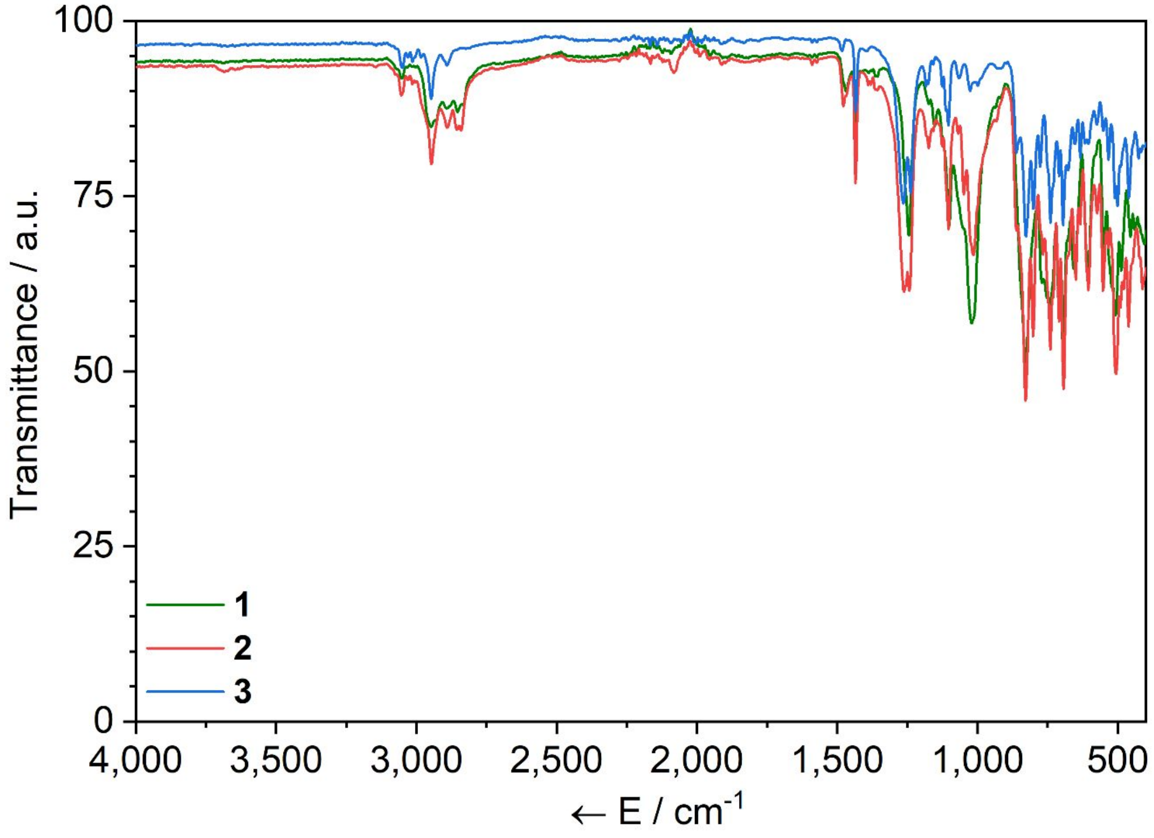

Figure S22. ATR-IR spectra of **1-3** between 398-4000 cm^{-1}.

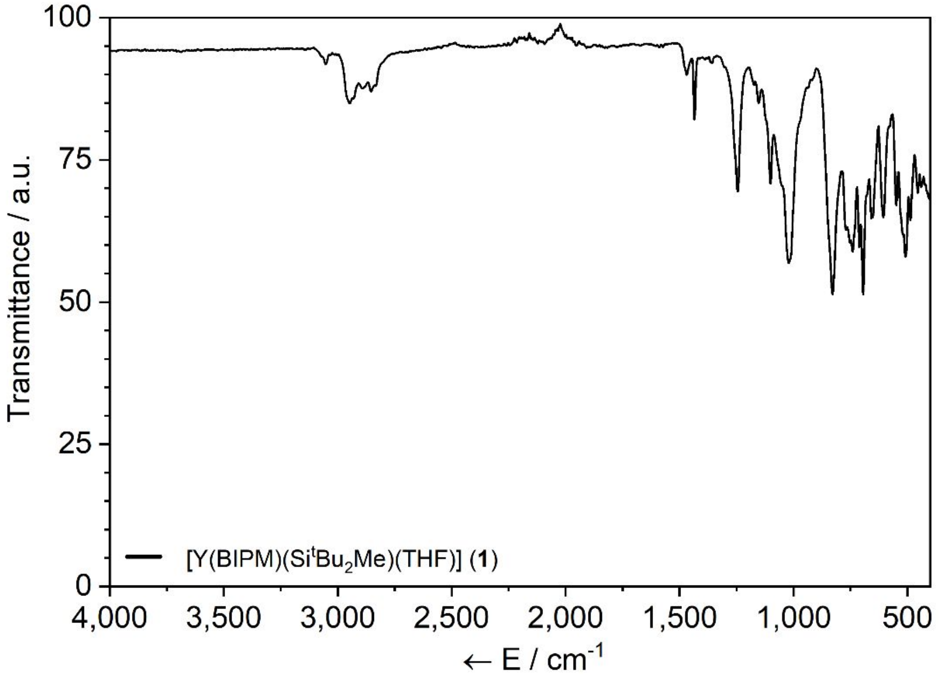

Figure S23. ATR-IR spectrum of **1** between 398-4000 cm^{-1}.

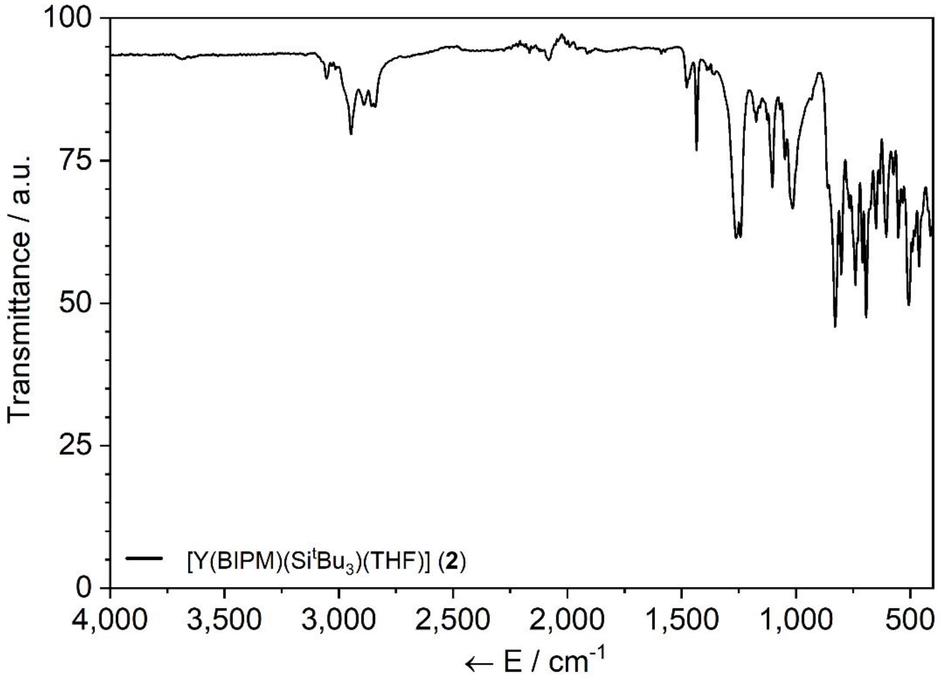

Figure S24. ATR-IR spectrum of **2** between 398-4000 cm^{-1}.

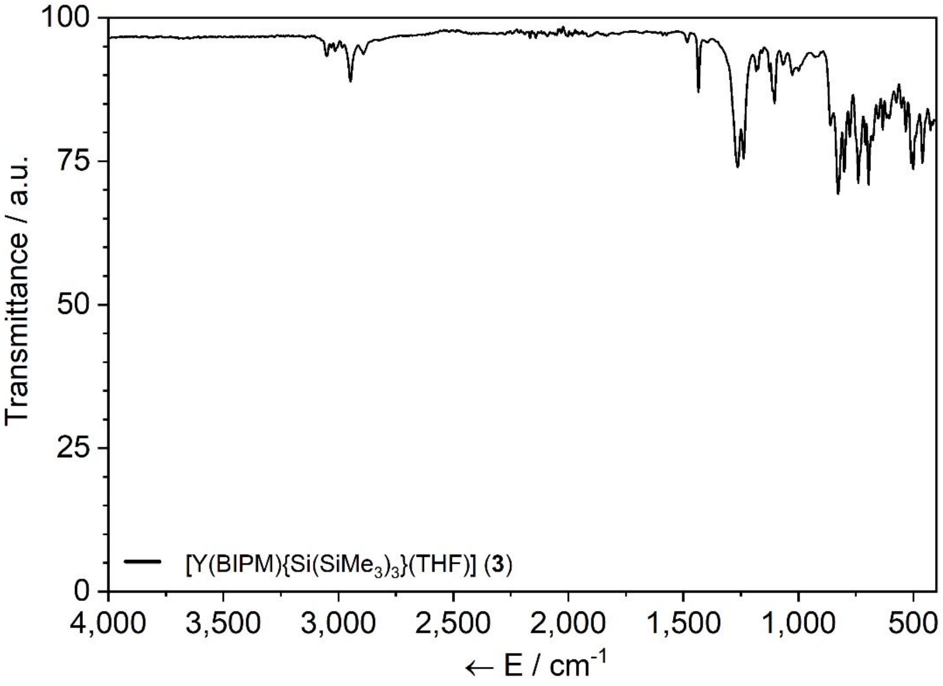

Figure S25. ATR-IR spectrum of **3** between 398-4000 cm^{-1}.

3. Crystallographic data

Table S1. Crystallographic Data for **1-3**.

	**1·toluene**	**2·toluene**	**3·0.5pentane**
Formula	$C_{44}H_{67}N_2OP_2Si_3Y \cdot C_7H_8$	$C_{47}H_{73}N_2OP_2Si_3Y \cdot C_7H_8$	$C_{46.50}H_{79}N_2OP_2Si_6Y$
Fw	967.25	1009.32	1001.50
crystal size, mm	1.057 × 0.759 × 0.654	0.388 × 0.359 × 0.223	0.182 × 0.156 × 0.101
crystal system	Monoclinic	Monoclinic	Monoclinic
space group	C*2/c*	P2_1/*c*	P2_1/*n*
a, Å	47.474(4)	23.536(3)	11.9273(4)
b, Å	11.7491(14)	12.9309(14)	18.8139(6)
c, Å	19.2997(16)	19.1953(18)	25.0673(8)
α, °	90	90	90
β, °	91.090(9)	105.158(11)	99.888(3)
γ, °	90	90	90
V, $Å^3$	10763.1(19)	5638.7(10)	5541.5(3)
Z	8	4	4
$\rho_{calcd,\ g\ cm^{-3}}$	1.194	1.189	1.200
μ, mm^{-1}	1.245	1.191	1.273
no. of reflections made	37021	35616	45186
no. of unique reflns R_{int}	9469, 0.1210	9918, 0.0897	13813, 0.0486
no. of reflns with $F^2 > 2\sigma(F^2)$	6730	6395	9934
transmn coeff range	0.444-0.557	0.658-0.878	0.790-1.000
R, R_w[a] ($F^2 > 2\sigma(F^2)$)	0.1486, 0.3068	0.0790, 0.1774	0.0430, 0.0833
R, R_w[a] (all data)	0.1792, 0.3207	0.1307, 0.2042	0.0769, 0.0912
S[a]	1.201	1.056	1.040
Parameters	669	727	567
max., min. diff map, e $Å^{-3}$	0.989, -1.284	1.933, -0.718	0.986, -0.685

[a] Conventional $R = \Sigma||F_o| - |F_c||/\Sigma|F_o|$; $R_w = [\Sigma w(F_o^2 - F_c^2)^2/\Sigma w(F_o^2)^2]^{1/2}$; $S = [\Sigma w(F_o^2 - F_c^2)^2/\text{no. data} - \text{no. params})]^{1/2}$ for all data.

Table S2. Crystallographic Data for **4**.

	**4·1.5d_6-benzene**
Formula	$C_{66}H_{103}N_6P_2Si_3Y\cdot1.5C_6D_6$
Fw	1341.87
crystal size, mm	0.104 × 0.077 × 0.032
crystal system	Monoclinic
space group	$P2_1/n$
a, Å	22.9948(5)
b, Å	12.2100(2)
c, Å	27.6852(5)
α, °	90
β, °	112.691(2)
γ, °	90
V, $Å^3$	7171.4(2)
Z	4
$\rho_{calcd,\ g\ cm^{-3}}$	1.243
μ, mm^{-1}	2.389
no. of reflections made	79018
no. of unique reflns R_{int}	14753, 0.0797
no. of reflns with $F^2 > 2\sigma(F^2)$	11540
transmn coeff range	0.780-1.000
R, R_w[a] ($F^2 > 2\sigma(F^2)$)	0.0439, 0.1047
R, R_w[a] (all data)	0.0617, 0.1128
S[a]	1.062
Parameters	797
max., min. diff map, e $Å^{-3}$	0.460, -1.295

[a] Conventional $R = \Sigma||F_o| - |F_c||/\Sigma|F_o|$; $R_w = [\Sigma w(F_o^2 - F_c^2)^2/\Sigma w(F_o^2)^2]^{1/2}$; $S = [\Sigma w(F_o^2 - F_c^2)^2/\text{no. data} - \text{no. params})]^{1/2}$ for all data.

4. NBO representations of selected frontier orbitals of 1-3

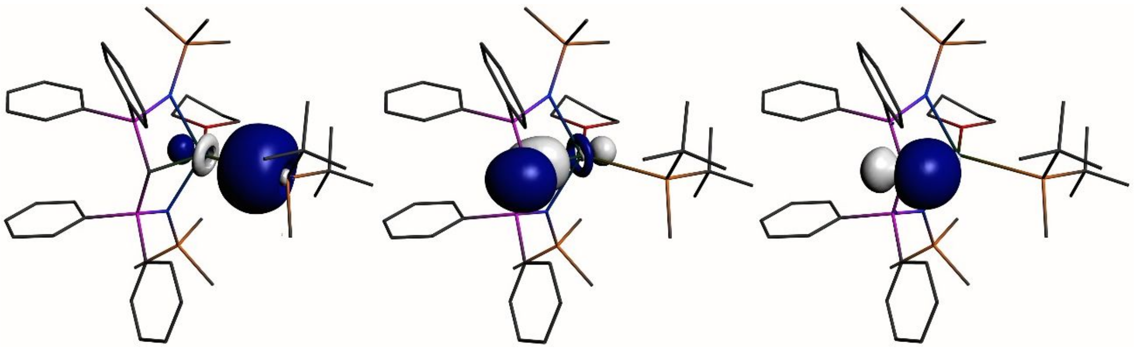

Figure S26. Selected NBOs for **1** (left-right): Y–Si σ-bond, Y=C σ-bond, Y=C π-bond. Hydrogen atoms omitted for clarity.

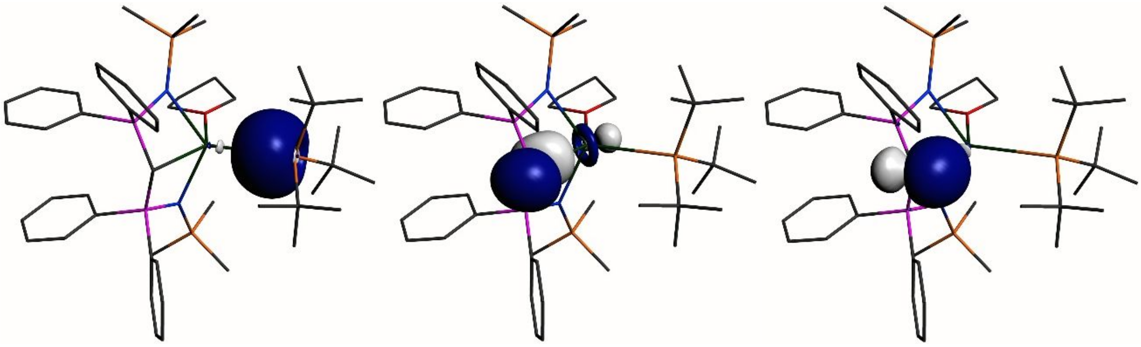

Figure S27. Selected NBOs for **2** (left-right): Y–Si σ-bond, Y=C σ-bond, Y=C π-bond. Hydrogen atoms omitted for clarity.

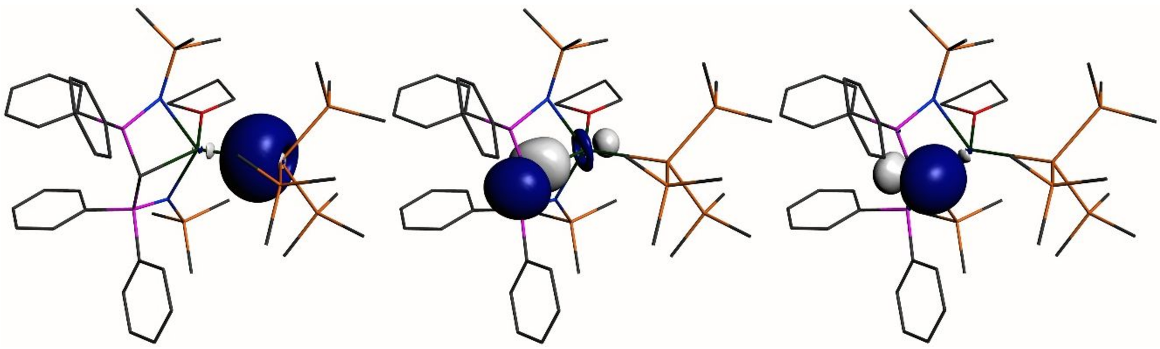

Figure S28. Selected NBOs for **1** (left-right): Y–Si σ-bond, Y=C σ-bond, Y=C π-bond. Hydrogen atoms omitted for clarity.

5. Optimized geometry coordinates for 1-3

Table S3. Final coordinates and energy from a single point energy calculation on geometry-optimized **1**.

1.C	-2.895849	0.107922	-5.067126
2.C	-1.749388	0.863756	-4.802564
3.C	-1.628814	-3.936981	-4.167141
4.C	-0.501384	-3.109143	-4.192254
5.C	-3.641548	-0.417364	-4.004781
6.C	-1.354790	1.093949	-3.481154
7.C	3.866157	2.320938	-3.259120
8.C	-2.293638	-4.168910	-2.957439
9.C	-0.035582	-2.525792	-3.011325
10.C	-3.246477	-0.184584	-2.685960
11.C	2.735688	1.479614	-2.670893
12.C	-1.063151	4.412322	-2.434363
13.C	-2.099160	0.580856	-2.410175
14.C	4.984953	2.156305	-2.216427
15.C	2.789878	-4.018935	-1.670743
16.C	-1.831340	-3.579623	-1.778065
17.C	-0.688153	-2.760877	-1.791420
18.C	4.413783	-1.584790	-0.934795
19.C	4.210118	2.167632	-0.905203
20.C	1.239158	4.473723	-0.439892
21.C	-4.034135	2.259125	-0.525483
22.C	-5.213309	2.664243	0.104214
23.C	-3.149640	1.375647	0.117356
24.C	-0.877499	-0.459904	0.137404
25.C	-1.616654	4.648703	0.562643
26.C	-0.141786	-4.527959	0.876197
27.C	3.361844	-3.360707	1.281113
28.C	-0.433120	-3.164485	1.053453
29.C	-5.519774	2.194696	1.387926
30.C	-3.474955	0.894488	1.393101
31.C	-0.325184	-5.435967	1.922597
32.C	-4.649318	1.307862	2.030226
33.C	-0.917720	-2.731410	2.296896
34.C	4.743292	0.789821	2.844880
35.C	3.920026	3.151273	2.883632
36.C	-0.798228	-4.991568	3.163076
37.C	-1.095253	-3.636803	3.347285
38.C	3.773139	1.766461	3.542462
39.C	0.474831	3.495624	3.785229
40.C	-0.780376	1.340829	3.923650
41.C	2.010612	-0.663987	4.186391
42.C	0.556373	2.033261	4.260978

43.C	4.200039	1.875989	5.018836
44.C	0.718937	2.020702	5.793087
45.H	-3.211421	-0.068995	-6.096958
46.H	-1.168397	1.283726	-5.625937
47.H	-1.989313	-4.399256	-5.088051
48.H	0.017050	-2.919754	-5.133957
49.H	-4.536727	-1.008998	-4.203684
50.H	4.156896	1.973736	-4.259240
51.H	-0.475554	1.700249	-3.266677
52.H	-0.355537	4.123799	-3.226903
53.H	-3.174416	-4.812423	-2.930418
54.H	3.563782	3.375417	-3.335006
55.H	0.849573	-1.890171	-3.024671
56.H	2.797290	0.425933	-2.978846
57.H	1.731696	1.862872	-2.889904
58.H	2.687791	-3.630475	-2.695086
59.H	-2.050678	4.019373	-2.720211
60.H	-1.131502	5.512386	-2.436813
61.H	5.501135	1.194468	-2.346761
62.H	5.730813	2.960964	-2.256732
63.H	3.709373	-4.625980	-1.632907
64.H	4.276991	-1.139357	-1.932377
65.H	-3.839638	-0.587839	-1.863400
66.H	1.940922	-4.695020	-1.495036
67.H	-3.809416	2.622986	-1.529314
68.H	1.936842	4.143391	-1.224937
69.H	5.301388	-2.236605	-0.983504
70.H	-2.354743	-3.766562	-0.838801
71.H	1.220020	5.575416	-0.456825
72.H	-5.895082	3.345102	-0.407779
73.H	4.007902	3.189522	-0.552346
74.H	4.646947	-0.779174	-0.221075
75.H	0.220292	-4.888240	-0.087341
76.H	4.681863	1.593837	-0.099787
77.H	1.651718	4.175587	0.537366
78.H	-2.682754	4.416277	0.426510
79.H	-1.494485	5.741545	0.480502
80.H	4.273376	-3.974945	1.194726
81.H	2.559340	-3.996681	1.683450
82.H	-1.342036	4.356222	1.586301
83.H	-6.439441	2.513854	1.881609
84.H	-0.097928	-6.492048	1.768858
85.H	3.556307	-2.569406	2.020389
86.H	3.594588	3.141290	1.830744
87.H	4.454667	0.600202	1.797191
88.H	-2.798549	0.184192	1.868411
89.H	5.773190	1.194439	2.839027
90.H	-1.154139	-1.673867	2.419698
91.H	0.450060	3.565877	2.686187
92.H	-4.886940	0.932593	3.027071

93.H	4.974050	3.489633	2.903384
94.H	3.322158	3.915850	3.398451
95.H	-0.981257	1.342179	2.842455
96.H	4.775186	-0.186220	3.348771
97.H	-0.441927	3.984086	4.167919
98.H	-0.938199	-5.700371	3.981403
99.H	1.072535	-1.225523	4.062837
100.H	2.810845	-1.310383	3.796448
101.H	-0.793553	0.294131	4.262867
102.H	1.330911	4.090578	4.137268
103.H	-1.467078	-3.284416	4.310957
104.H	-1.621966	1.860289	4.417699
105.H	5.267006	2.161786	5.095390
106.H	2.184876	-0.541128	5.268469
107.H	4.077441	0.922276	5.554330
108.H	3.621673	2.639815	5.557805
109.H	1.619909	2.557522	6.122069
110.H	-0.148516	2.514272	6.272785
111.H	0.773685	0.997471	6.194535
112.N	-0.432071	2.059355	-0.613701
113.N	1.504728	-1.574166	-0.355742
114.O	2.918659	1.529593	-1.213377
115.P	-1.568489	0.853963	-0.668811
116.P	-0.117005	-1.915459	-0.264174
117.Si	-0.517255	3.806861	-0.719791
118.Si	2.916022	-2.620298	-0.397292
119.Si	1.947047	1.033887	3.263212
120.Y	1.207611	0.655188	0.318258

Energy: –687.97246422 eV

Table S4. Final coordinates and energy from a single point energy calculation on geometry-optimized **2**.

1.C	0.927475	0.894643	-5.663419
2.C	-0.448392	0.248768	-5.425960
3.C	1.711260	0.488253	-4.417364
4.C	-0.638243	0.430614	-3.924784
5.C	0.773045	-3.125714	-3.535144
6.C	0.962459	3.839852	-2.787131
7.C	5.344414	0.030662	-2.426796
8.C	-1.460188	-4.545257	-2.120154
9.C	-4.654350	2.047058	-1.691927
10.C	4.370301	-2.138680	-1.772639
11.C	-4.702232	-1.997901	-1.662778
12.C	-1.567041	4.799493	-1.473680
13.C	-3.317946	1.944719	-1.298374
14.C	5.075352	-0.885054	-1.215021
15.C	-3.355886	-1.792371	-1.354580
16.C	-5.673416	1.909868	-0.744107
17.C	1.390018	-4.642749	-0.981640
18.C	4.081880	2.750094	-0.801055
19.C	6.442934	-1.323211	-0.649641
20.C	-5.601773	-2.368135	-0.656700
21.C	-2.978248	1.714754	0.042522
22.C	-2.885195	-1.956241	-0.042689
23.C	1.016009	4.839266	0.111303
24.C	-5.346759	1.662529	0.594540
25.C	4.523690	1.883209	0.396069
26.C	-5.146381	-2.525310	0.656510
27.C	6.050022	2.034301	0.562400
28.C	-4.009479	1.561673	0.985879
29.C	-0.687337	-0.056700	0.792707
30.C	-3.798797	-2.317349	0.963161
31.C	3.763863	-2.483215	1.598478
32.C	3.854226	2.492551	1.645475
33.C	-0.975787	-4.158367	1.668338
34.C	3.946094	-0.971378	1.846389
35.C	-0.835554	-2.765018	1.784598
36.C	-1.083109	2.520809	2.069733
37.C	-1.853079	3.673451	2.299610
38.C	5.245333	-0.774550	2.655919
39.C	-0.741221	-4.991568	2.764072
40.C	2.765737	-0.532702	2.740134
41.C	-0.132068	2.137054	3.029212
42.C	-0.482504	-2.221572	3.027243
43.C	-1.676089	4.424213	3.466274
44.C	-0.370813	-4.439956	3.997643
45.C	-0.248957	-3.053401	4.128218
46.C	0.054589	2.893507	4.188872

47.C	-0.719603	4.038722	4.412146
48.H	1.412351	0.540967	-6.582550
49.H	-1.251790	0.726443	-6.002121
50.H	-0.423719	-0.819493	-5.684431
51.H	0.836361	1.988888	-5.719122
52.H	2.204991	-0.486567	-4.537113
53.H	2.445473	1.237107	-4.095902
54.H	0.980269	-4.069216	-4.066328
55.H	0.042861	-2.562491	-4.136455
56.H	-1.056561	1.416136	-3.674169
57.H	0.363778	3.395502	-3.597972
58.H	-1.243676	-0.350208	-3.448318
59.H	1.715895	-2.558136	-3.510017
60.H	4.409186	0.384369	-2.891352
61.H	1.165891	4.886381	-3.067694
62.H	5.906760	-0.526037	-3.201493
63.H	-4.900748	2.241475	-2.737442
64.H	-1.171060	-5.472398	-2.642516
65.H	1.932835	3.323392	-2.746276
66.H	-2.171235	-4.007458	-2.764860
67.H	4.995995	-2.622792	-2.547145
68.H	-5.051530	-1.873870	-2.689207
69.H	-2.130628	4.416078	-2.336947
70.H	5.942812	0.913630	-2.165406
71.H	3.406831	-1.883739	-2.243330
72.H	-2.518020	2.063400	-2.027450
73.H	-2.651079	-1.522895	-2.141172
74.H	4.527075	2.428572	-1.751170
75.H	7.057100	-1.781959	-1.449221
76.H	-1.290210	5.843711	-1.695401
77.H	1.455704	-5.599765	-1.525477
78.H	-6.718644	1.994706	-1.046396
79.H	-2.011576	-4.835399	-1.214468
80.H	4.166350	-2.887709	-0.998239
81.H	2.386065	-4.177921	-1.002792
82.H	-6.653422	-2.535424	-0.896228
83.H	2.988732	2.728196	-0.917935
84.H	4.369170	3.806878	-0.642346
85.H	-2.253452	4.818381	-0.614989
86.H	7.015369	-0.478881	-0.241042
87.H	1.176055	5.853971	-0.289595
88.H	6.603602	1.726792	-0.336186
89.H	6.340502	-2.073461	0.146522
90.H	1.150217	-4.862931	0.068523
91.H	2.000666	4.402697	0.324246
92.H	6.306043	3.095073	0.752124
93.H	-1.277260	-4.601155	0.720127
94.H	-6.137206	1.547219	1.337610
95.H	4.619026	-2.929464	1.072261
96.H	0.479979	4.936539	1.068041

97.H	-5.840456	-2.815322	1.447057
98.H	2.853424	-2.700391	1.019753
99.H	6.436150	1.449214	1.408619
100.H	2.757811	2.396438	1.619937
101.H	4.087218	3.572115	1.710206
102.H	-2.605106	3.982110	1.572679
103.H	-3.764673	1.373715	2.032450
104.H	-3.452112	-2.451515	1.988939
105.H	6.142650	-1.075319	2.097805
106.H	1.799966	-0.668071	2.231294
107.H	3.667012	-3.012281	2.564463
108.H	4.212138	2.030909	2.575421
109.H	-0.847166	-6.072278	2.655938
110.H	5.383100	0.268733	2.974076
111.H	0.453573	1.234005	2.855473
112.H	2.838564	0.518045	3.046406
113.H	-0.395152	-1.138134	3.109675
114.H	5.209798	-1.389798	3.576053
115.H	-2.288844	5.310928	3.636538
116.H	2.743834	-1.146137	3.660577
117.H	-0.181239	-5.091430	4.852855
118.H	0.802007	2.585895	4.922268
119.H	0.032741	-2.616372	5.087584
120.H	-0.581564	4.625660	5.322034
121.N	-0.163844	-2.014472	-0.945397
122.N	-0.234929	2.145444	-0.658545
123.O	0.707820	0.352809	-3.347204
124.P	-1.116735	-1.632196	0.357019
125.P	-1.215149	1.524401	0.526087
126.Si	0.107021	-3.523053	-1.802439
127.Si	0.025764	3.822758	-1.133918
128.Si	3.849194	0.018699	0.104599
129.Y	0.923404	0.092654	-0.926488

Energy: −735.95662085 eV

Table S5. Final coordinates and energy from a single point energy calculation on geometry-optimized **3**.

1.C	-1.766607	-4.046952	-4.335570
2.C	-1.295163	-2.731942	-4.394971
3.C	-0.330919	4.657750	-3.738479
4.C	0.333238	3.428105	-3.667281
5.C	3.502259	0.977553	-3.569292
6.C	5.658382	-1.128584	-3.133618
7.C	-2.107879	-4.610831	-3.099506
8.C	-1.159184	-1.982458	-3.221211
9.C	2.735060	-1.926869	-3.056090
10.C	-1.332062	4.958988	-2.808619
11.C	-0.001719	2.509204	-2.669481
12.C	-1.973035	-3.861076	-1.929934
13.C	-1.488308	-2.543187	-1.980722
14.C	-1.654324	4.044434	-1.801958
15.C	-0.989485	2.809763	-1.719835
16.C	6.452794	2.224646	-1.080620
17.C	-3.997651	1.956603	-1.074361
18.C	-5.365843	2.080214	-0.823545
19.C	-0.830263	0.053193	-0.824758
20.C	3.748516	3.445682	-0.433956
21.C	1.246281	-4.617957	-0.396414
22.C	-4.155082	-1.629695	-0.445745
23.C	7.185280	-1.275243	0.345705
24.C	-3.083064	1.852176	-0.009319
25.C	-5.406926	-1.802246	0.149215
26.C	5.046386	-3.433453	0.262892
27.C	-5.841940	2.089757	0.493623
28.C	-2.979996	-1.767559	0.314177
29.C	0.271867	5.026371	0.812156
30.C	-3.572424	1.848434	1.304249
31.C	-5.503727	-2.119397	1.509470
32.C	-4.942693	1.967368	1.557051
33.C	5.505006	2.319142	1.818863
34.C	-3.089210	-2.082169	1.676253
35.C	-0.898051	-4.824358	1.788927
36.C	-4.340418	-2.259985	2.273197
37.C	1.723023	-3.413878	2.369973
38.C	5.323493	-1.608571	2.707771
39.C	1.546054	3.178400	2.873846
40.C	-1.396197	3.881219	3.075730
41.C	-0.561375	-0.287762	3.899471
42.C	1.807093	-0.235642	4.280731
43.C	-0.322591	0.186412	5.324270
44.C	1.078174	-0.381375	5.619286
45.H	-1.876040	-4.632279	-5.250986
46.H	-1.036722	-2.286175	-5.357053

47.H	3.524557	0.717711	-4.640586
48.H	-0.072349	5.376791	-4.518312
49.H	5.621333	-1.289348	-4.224529
50.H	1.114931	3.183867	-4.389572
51.H	2.749720	-2.043146	-4.152553
52.H	4.210220	1.803770	-3.410111
53.H	2.497923	1.354307	-3.334180
54.H	-2.487450	-5.632886	-3.048275
55.H	-0.810573	-0.948513	-3.246008
56.H	6.444631	-0.388100	-2.927713
57.H	-1.861260	5.912332	-2.861907
58.H	5.960291	-2.079686	-2.670635
59.H	3.008737	-2.899347	-2.620402
60.H	1.699664	-1.712439	-2.753735
61.H	0.491210	1.537549	-2.618022
62.H	6.197557	2.154302	-2.148184
63.H	-3.634919	1.952293	-2.103345
64.H	-6.061845	2.171397	-1.658445
65.H	3.407174	3.363641	-1.476489
66.H	-4.090450	-1.399345	-1.511054
67.H	-2.436530	4.292522	-1.082782
68.H	6.895727	3.220366	-0.909853
69.H	7.225000	1.470281	-0.872417
70.H	0.543995	-4.853948	-1.209555
71.H	7.354509	-1.306840	-0.740568
72.H	-2.262183	-4.300469	-0.974106
73.H	5.090106	-3.550225	-0.830382
74.H	2.061322	-4.021108	-0.827684
75.H	-6.310940	-1.694030	-0.452653
76.H	4.260335	4.415693	-0.322380
77.H	1.675369	-5.564766	-0.029928
78.H	0.986550	4.866166	-0.008466
79.H	7.521207	-0.293402	0.710943
80.H	2.851426	3.467769	0.199002
81.H	7.827491	-2.042367	0.810908
82.H	-0.654723	5.423745	0.373897
83.H	5.816983	-4.090903	0.699070
84.H	-6.911290	2.188229	0.689241
85.H	4.065796	-3.802364	0.596052
86.H	-1.762594	-4.994931	1.130195
87.H	0.686749	5.804424	1.474734
88.H	5.934441	3.330156	1.910672
89.H	-6.483436	-2.262117	1.969959
90.H	-0.449021	-5.808200	2.003488
91.H	6.282329	1.598705	2.114773
92.H	-2.870355	1.764278	2.131967
93.H	2.505490	-2.710795	2.044757
94.H	-2.176976	-2.214056	2.256781
95.H	-5.306478	1.967130	2.585500
96.H	4.683420	2.239184	2.547084

97.H	2.370398	2.724752	2.301802
98.H	-2.341207	4.137396	2.574586
99.H	2.227783	-4.360812	2.618055
100.H	-1.285674	-4.425157	2.738746
101.H	6.121629	-2.256843	3.105765
102.H	5.487122	-0.594790	3.104003
103.H	4.368737	-1.981613	3.108130
104.H	-4.407353	-2.522148	3.331084
105.H	1.918180	4.141597	3.257989
106.H	1.270493	-3.026396	3.296171
107.H	-1.094322	4.752318	3.680094
108.H	-1.249848	0.333702	3.314078
109.H	-1.600558	3.052686	3.771969
110.H	1.336643	2.539176	3.744438
111.H	-0.900912	-1.334231	3.868386
112.H	2.480462	-1.070675	4.054279
113.H	2.359722	0.709377	4.203532
114.H	-0.319450	1.285204	5.367605
115.H	-1.089816	-0.185683	6.017037
116.H	1.008111	-1.440534	5.907059
117.H	1.596520	0.157358	6.423238
118.N	-0.175941	-2.105362	0.584054
119.N	-0.339049	1.985856	0.946459
120.O	0.749233	-0.207398	3.250245
121.P	-1.323609	-1.524921	-0.458448
122.P	-1.294826	1.611592	-0.354116
123.Si	3.942346	-0.556725	-2.519851
124.Si	3.772280	-0.093166	-0.168099
125.Si	4.911596	2.007251	0.027104
126.Si	5.360589	-1.625233	0.792847
127.Si	0.417089	-3.693613	1.021962
128.Si	-0.018178	3.465511	1.835810
129.Y	0.882257	-0.051795	0.820370

Energy: −726.60939062 eV

6. NMR spectra of reactivity studies of 1

6.1. Benzophenone (1 equivalent)

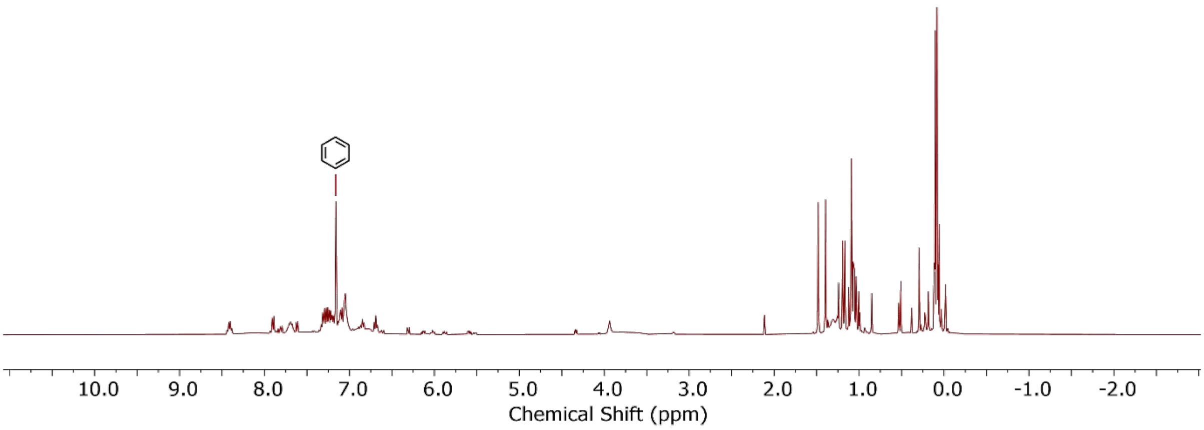

Figure S29. ^{1}H NMR spectrum of **1** + Ph_2CO in d_6-benzene.

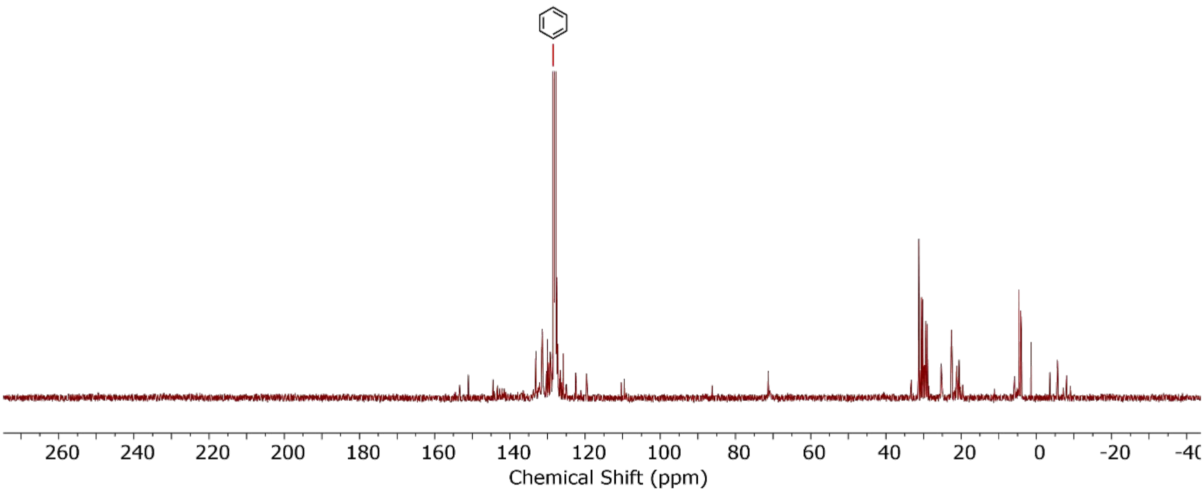

Figure S30. $^{13}C\{^{1}H\}$ NMR spectrum of **1** + Ph_2CO in d_6-benzene.

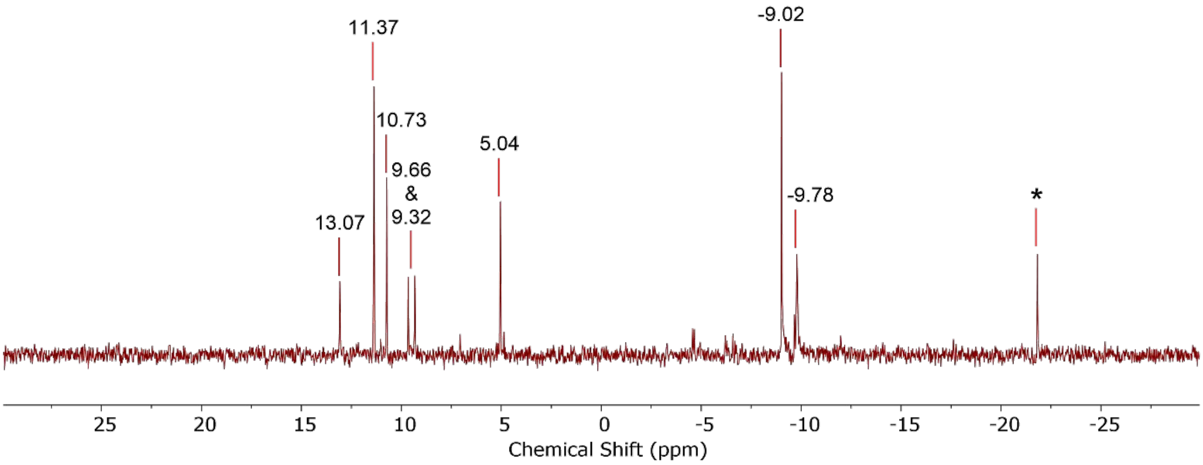

Figure S31. $^{29}Si\{^{1}H\}$ NMR spectrum of **1** + Ph_2CO in d_6-benzene. * denotes silicone grease.

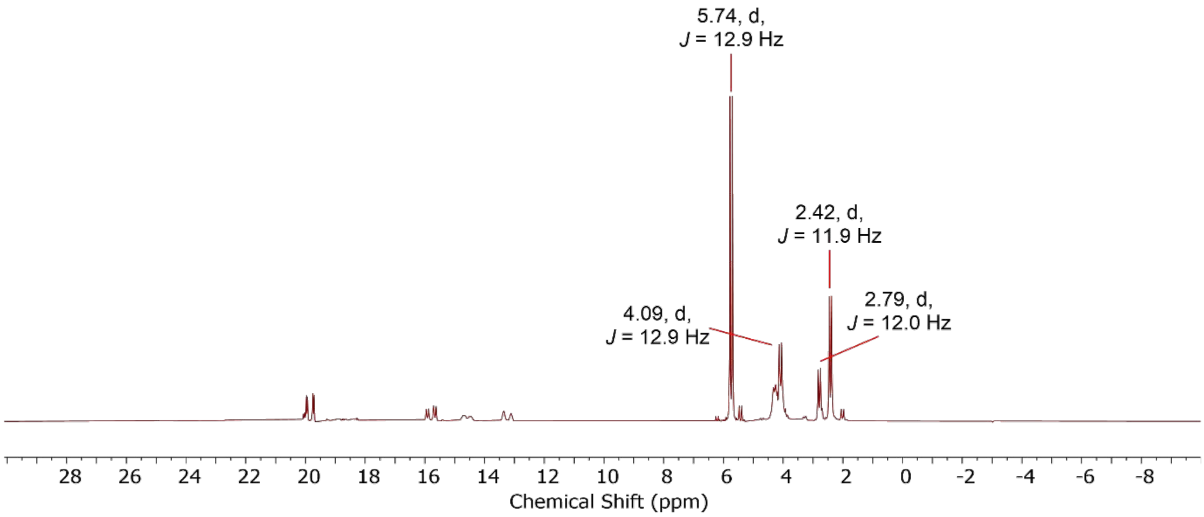

Figure S32. $^{31}P\{^1H\}$ NMR spectrum of **1** + Ph_2CO in d_6-benzene.

6.2. Benzophenone (2 equivalents)

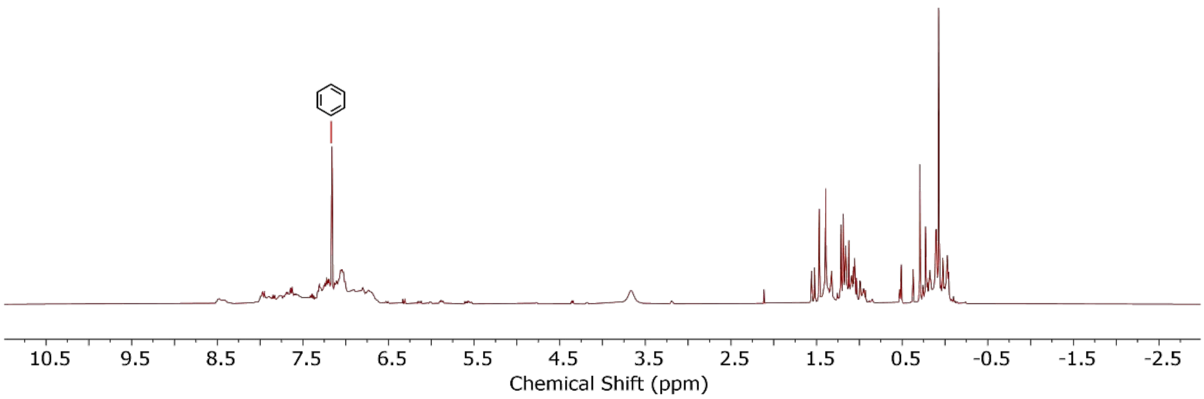

Figure S33. ^1H NMR spectrum of **1** + 2 Ph_2CO in d_6-benzene.

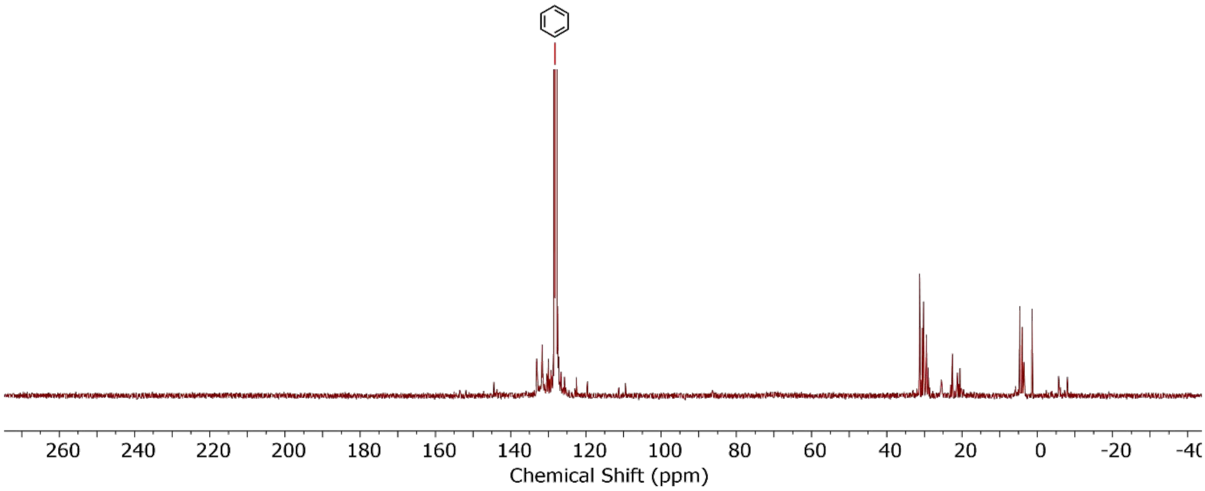

Figure S34. $^{13}C\{^1H\}$ NMR spectrum of **1** + 2 Ph_2CO in d_6-benzene.

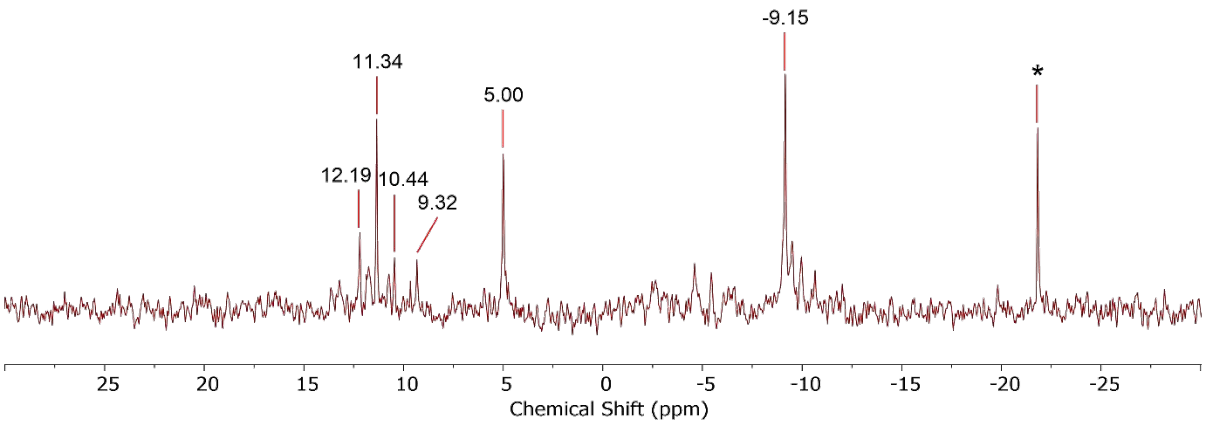

Figure S35. $^{29}Si\{^1H\}$ NMR spectrum of **1** + 2 Ph_2CO in d_6-benzene. * denotes silicone grease.

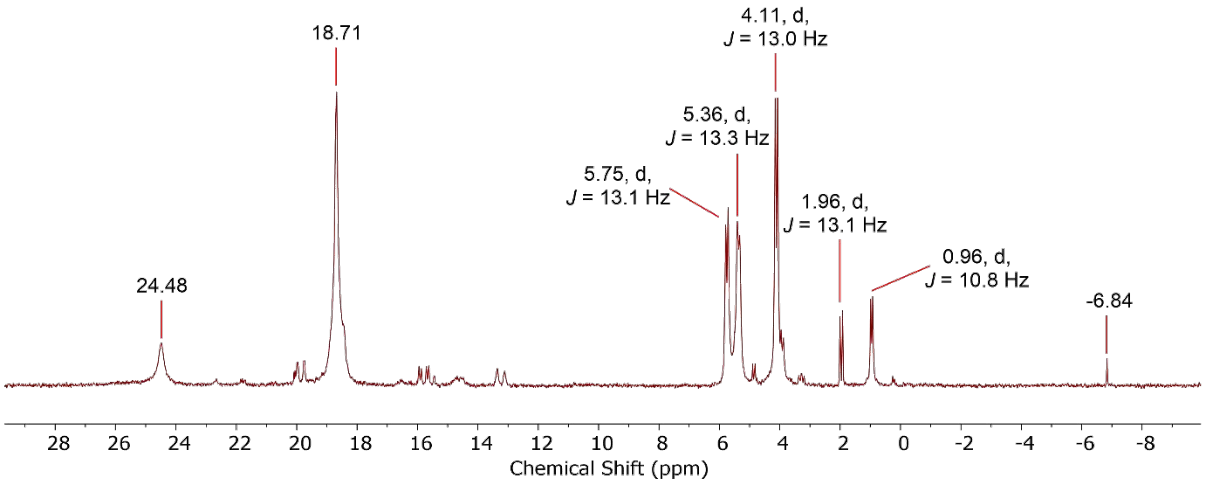

Figure S36. $^{31}P\{^1H\}$ NMR spectrum of **1** + 2 Ph_2CO in d_6-benzene.

6.3. Azobenzene (1 equivalent)

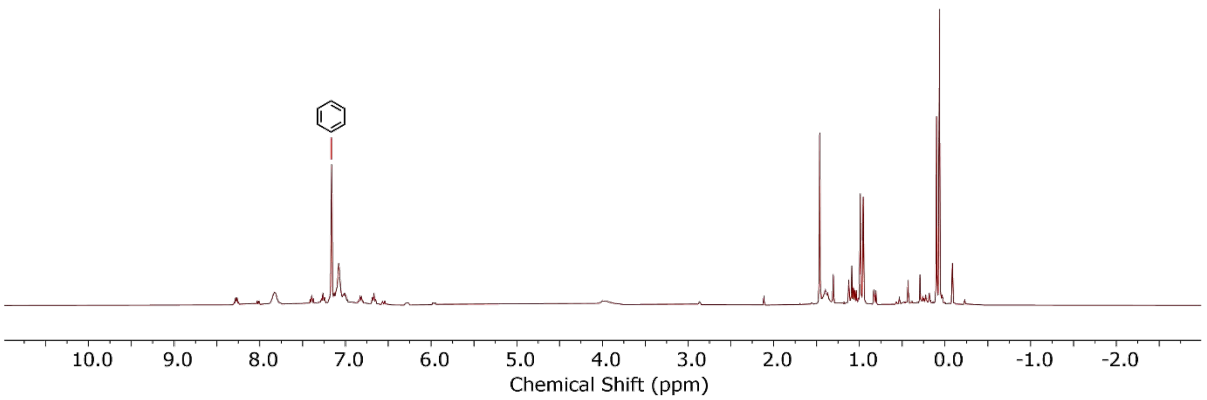

Figure S37. ^1H NMR spectrum of **1** + PhN=NPh in d_6-benzene.

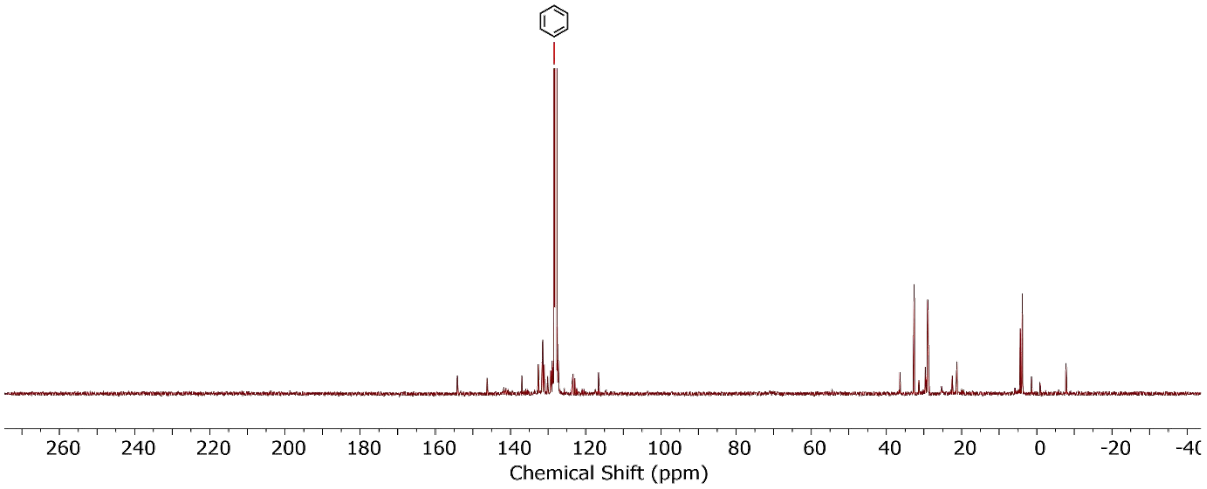

Figure S38. $^{13}C\{^1H\}$ NMR spectrum of **1** + PhN=NPh in d_6-benzene.

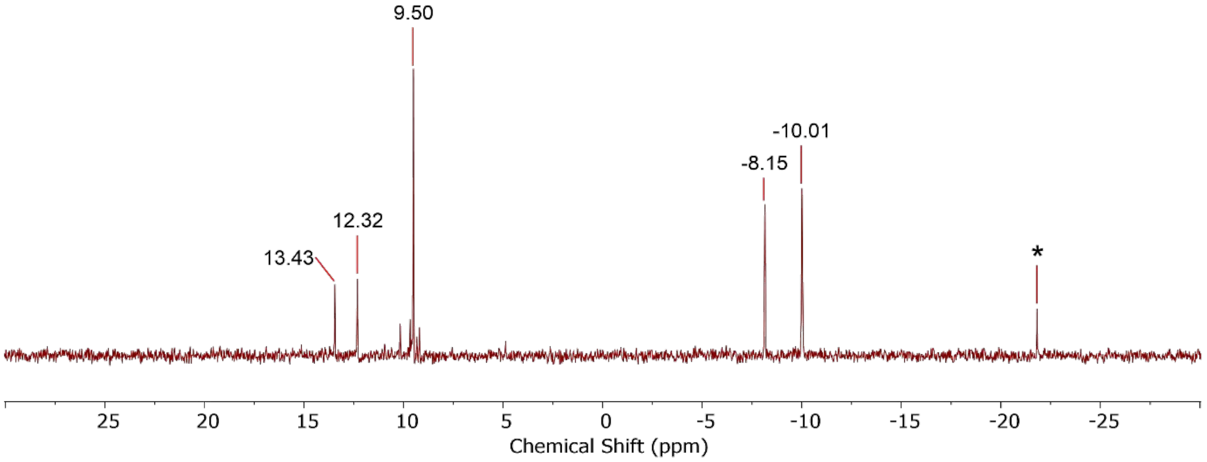

Figure S39. $^{29}Si\{^1H\}$ NMR spectrum of **1** + PhN=NPh in d_6-benzene. * denotes silicone grease.

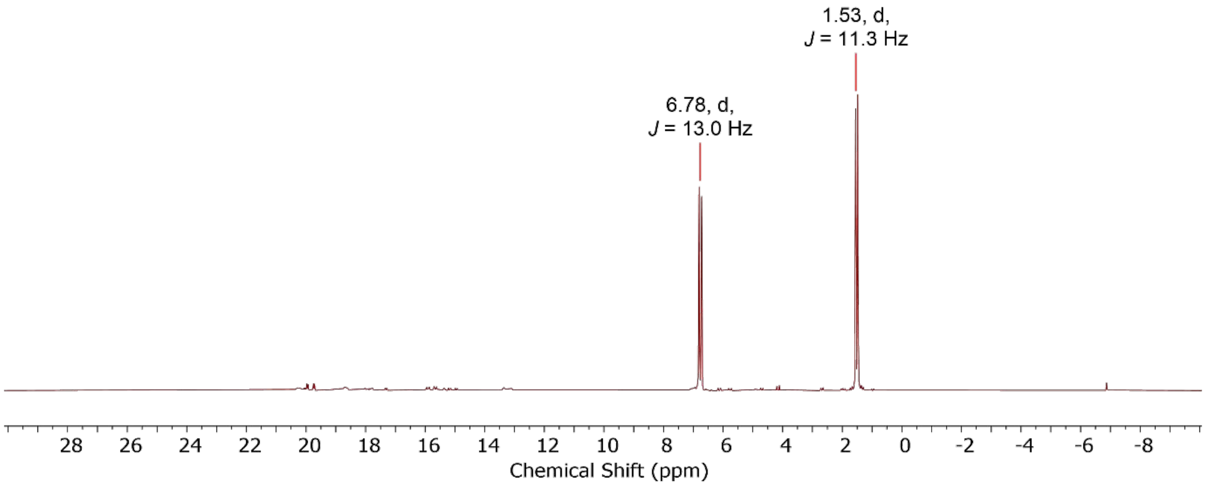

Figure S40. $^{31}P\{^1H\}$ NMR spectrum of **1** + PhN=NPh in d_6-benzene.

6.4. Azobenzene (2 equivalents)

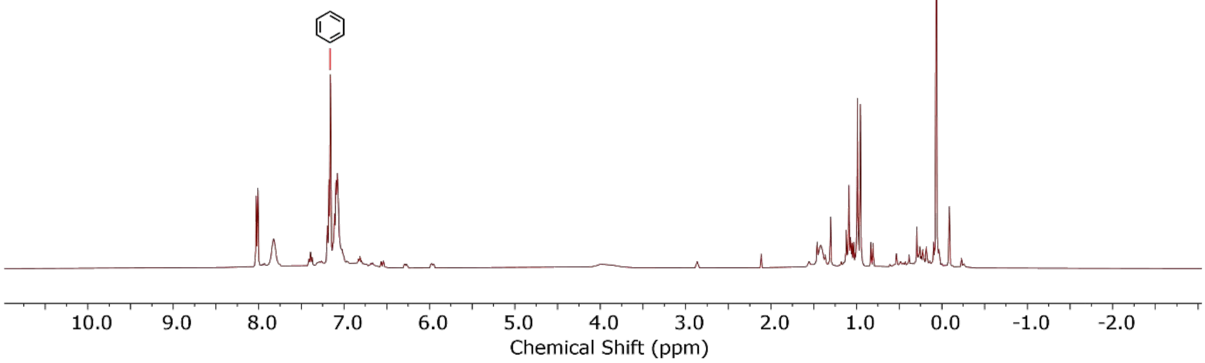

Figure S41. ^{1}H NMR spectrum of **1** + 2 PhN=NPh in d_6-benzene.

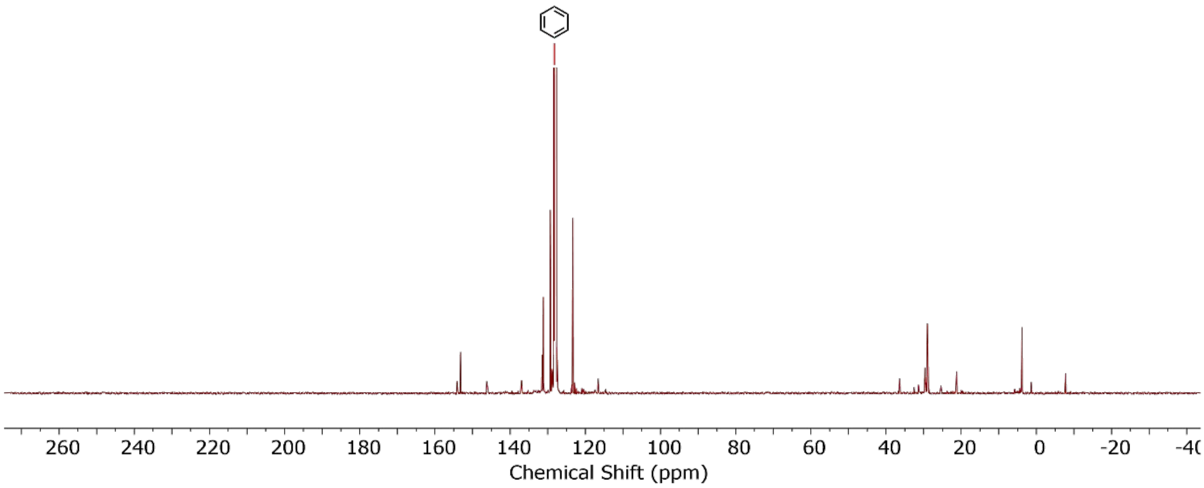

Figure S42. $^{13}C\{^{1}H\}$ NMR spectrum of **1** + 2 PhN=NPh in d_6-benzene.

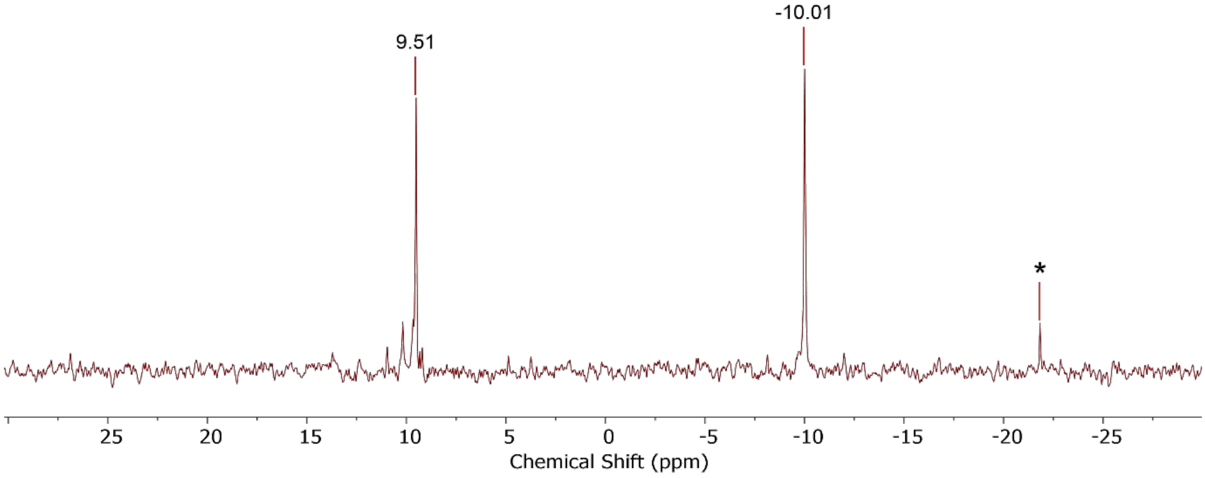

Figure S43. $^{29}Si\{^{1}H\}$ NMR spectrum of **1** + 2 PhN=NPh in d_6-benzene. * denotes silicone grease.

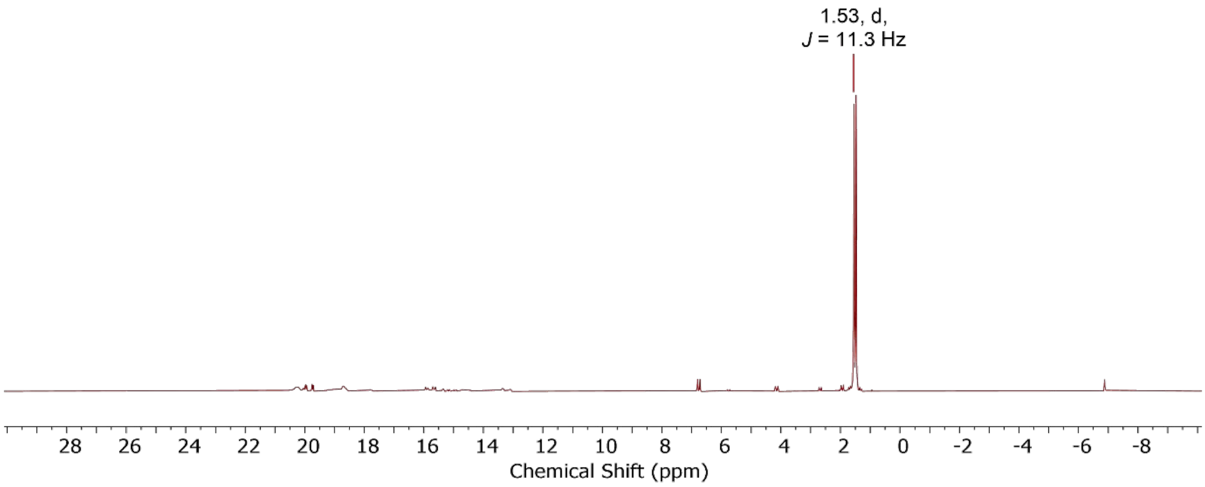

Figure S44. $^{31}P\{^1H\}$ NMR spectrum of **1** + 2 PhN=NPh in d_6-benzene.

6.5. *N*,*N'*-dicyclohexyl-carbodiimide (1 equivalent)

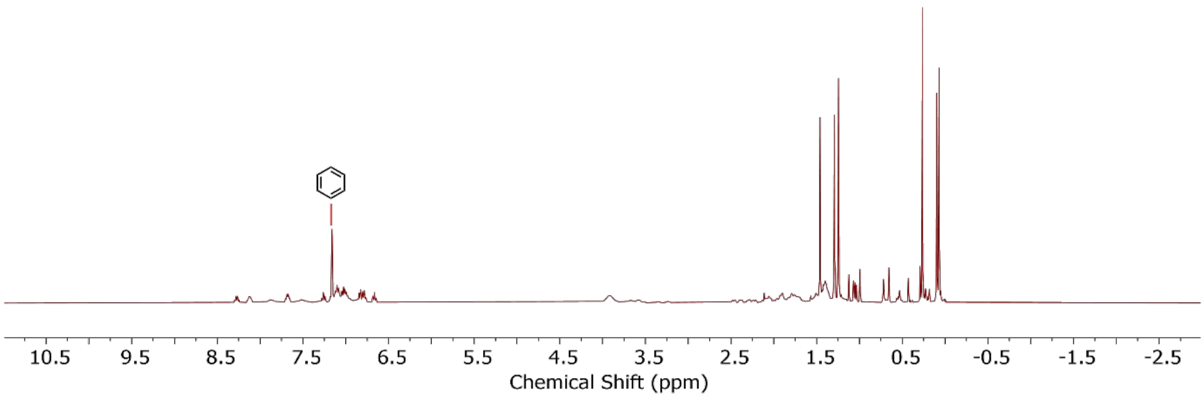

Figure S45. ^1H NMR spectrum of **1** + CyN=C=NCy in d_6-benzene.

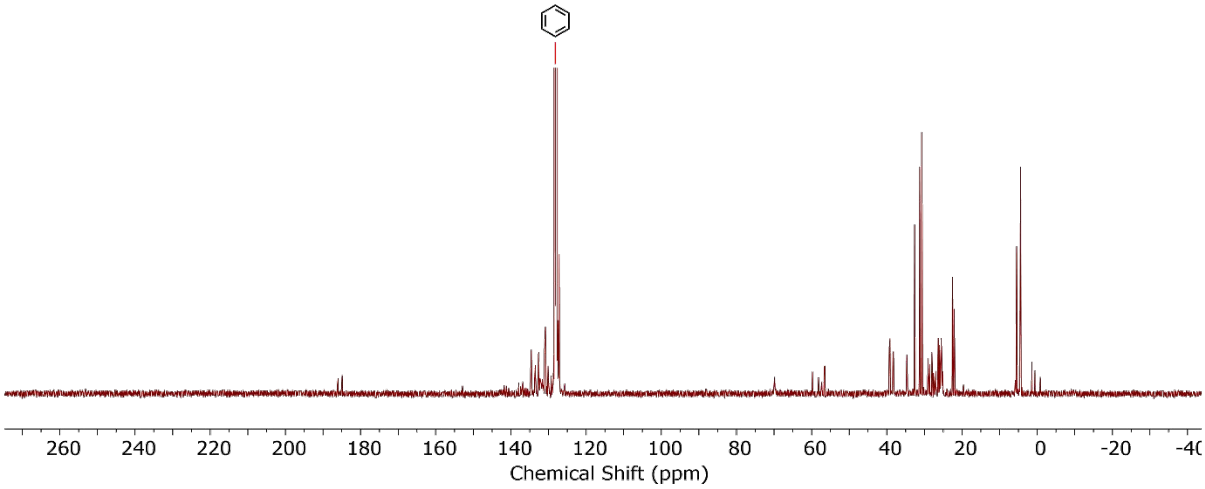

Figure S46. $^{13}C\{^1H\}$ NMR spectrum of **1** + CyN=C=NCy in d_6-benzene.

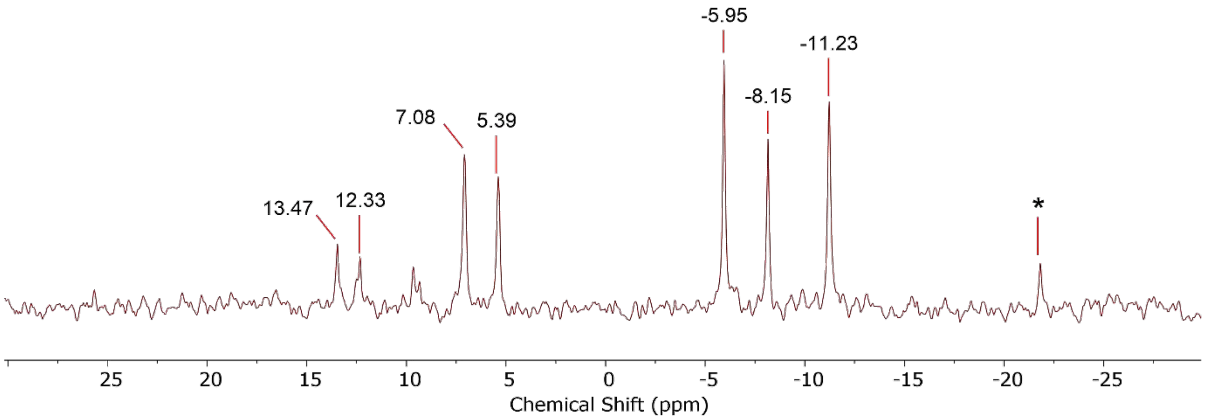

Figure S47. $^{29}Si\{^1H\}$ NMR spectrum of **1** + CyN=C=NCy in d_6-benzene. * denotes silicone grease.

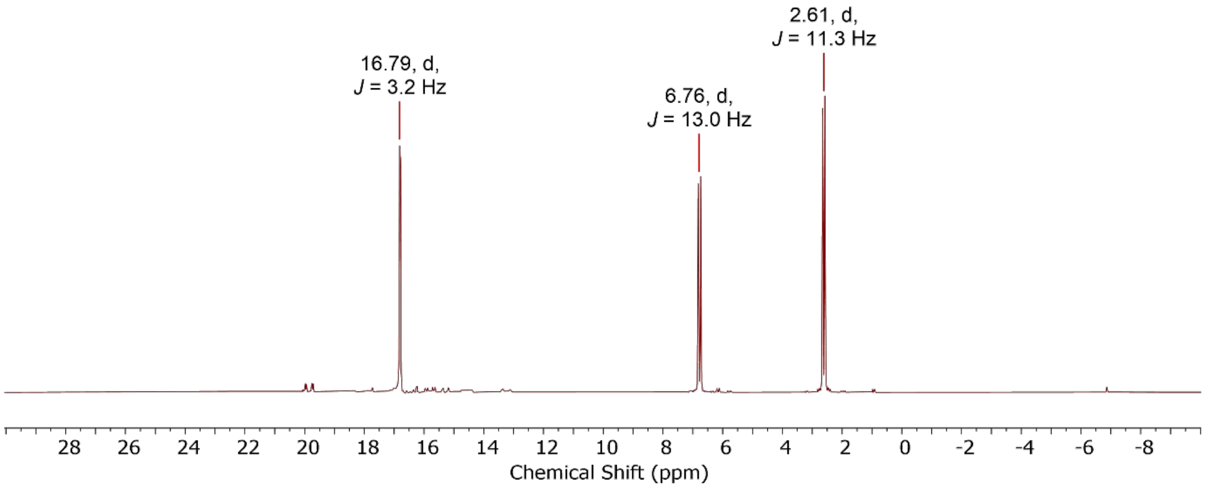

Figure S48. $^{31}P\{^1H\}$ NMR spectrum of **1** + CyN=C=NCy in d_6-benzene.

6.6. *N*,*N'*-dicyclohexyl-carbodiimide (2 equivalents)

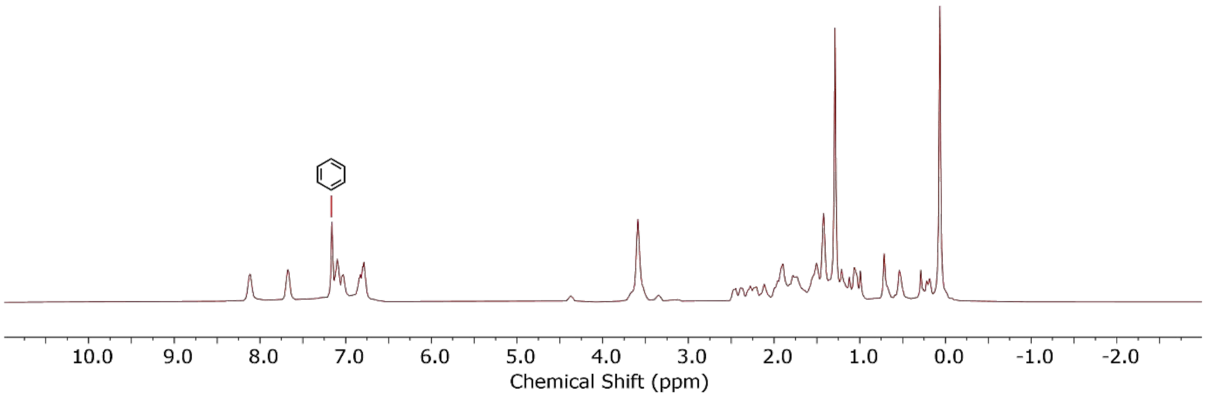

Figure S49. ^1H NMR spectrum of **1** + 2 CyN=C=NCy in d_6-benzene.

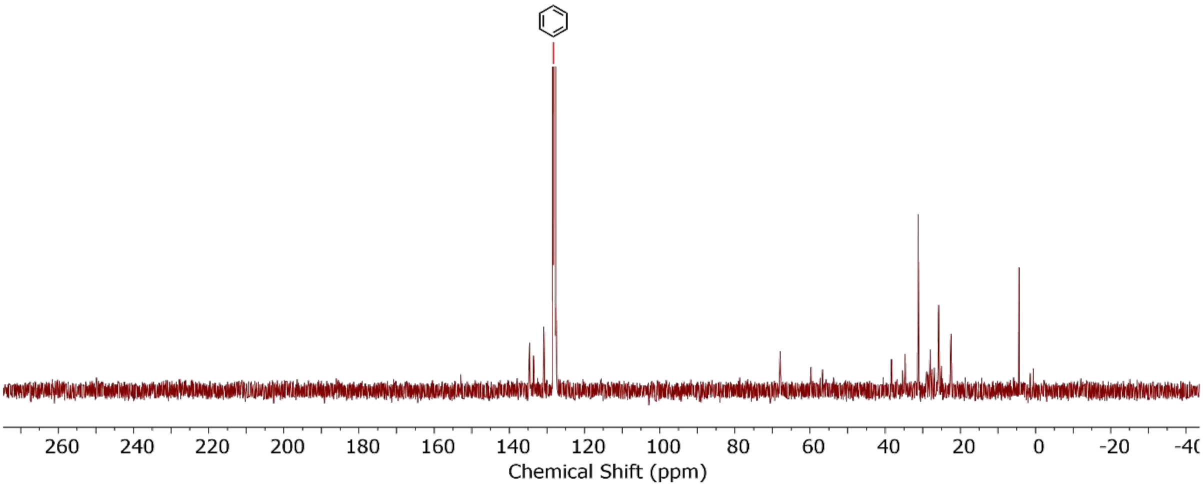

Figure S50. $^{13}C\{^{1}H\}$ NMR spectrum of **1** + 2 CyN=C=NCy in d_6-benzene.

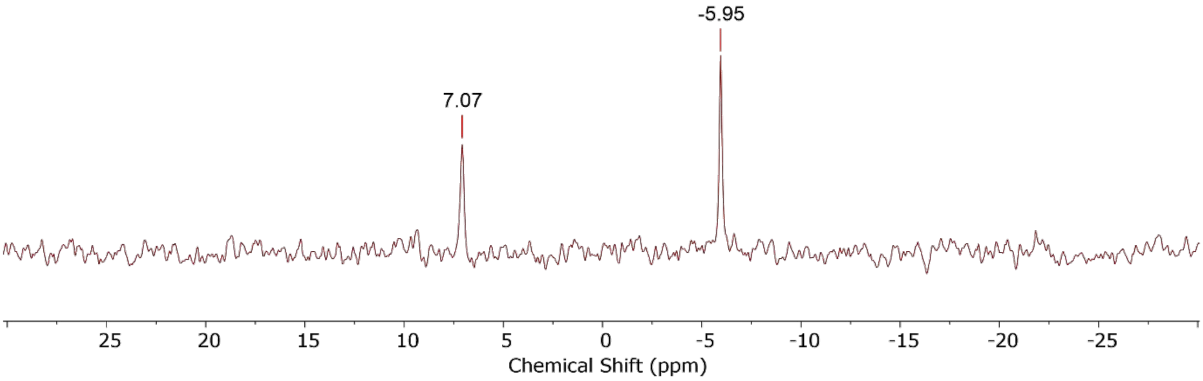

Figure S51. $^{29}Si\{^{1}H\}$ NMR spectrum of **1** + 2 CyN=C=NCy in d_6-benzene.

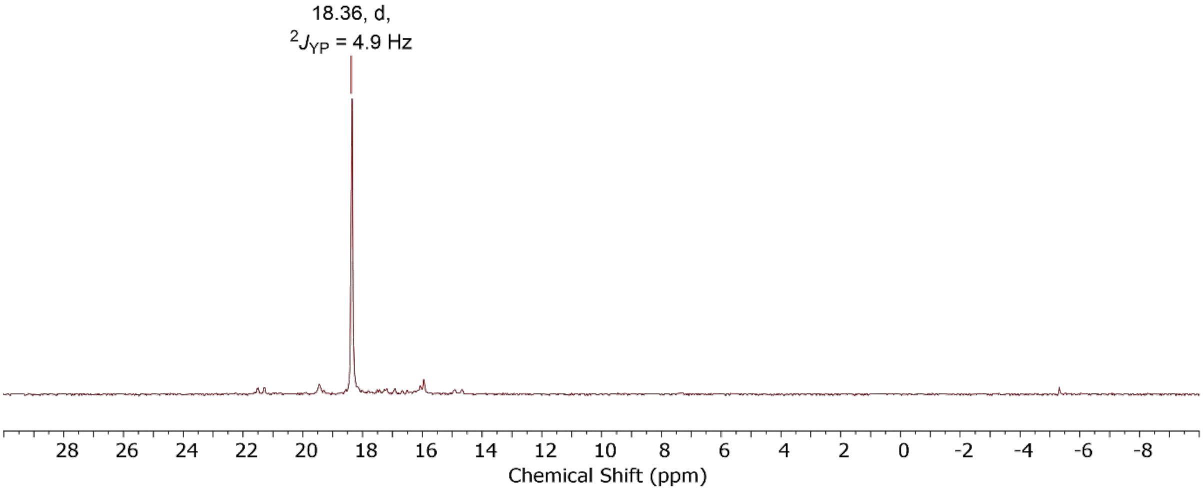

Figure S52. $^{31}P\{^{1}H\}$ NMR spectrum of **1** + 2 CyN=C=NCy in d_6-benzene.

7. Characterization data for 4

7.1. NMR spectra of 4

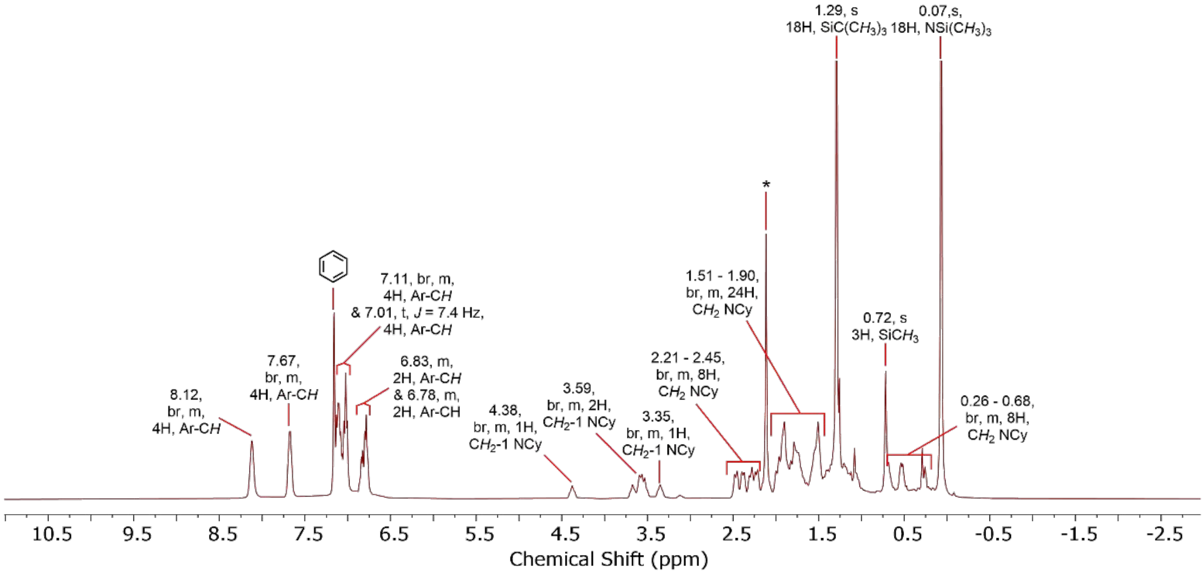

Figure S53. ^{1}H NMR spectrum of **4** in d_6-benzene. * denotes residual toluene.

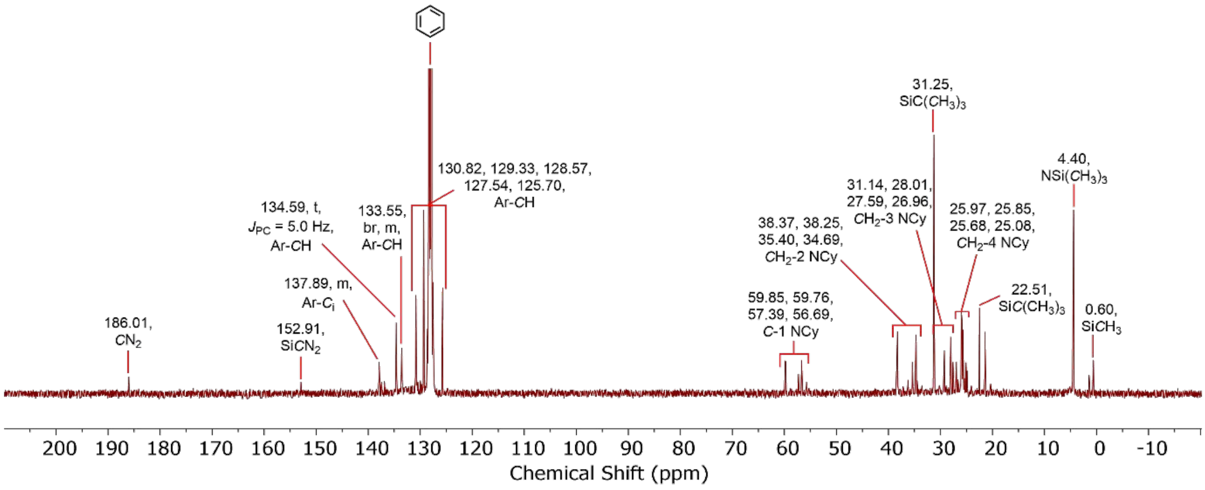

Figure S54. ^{13}C{^{1}H} NMR spectrum of **4** in d_6-benzene.

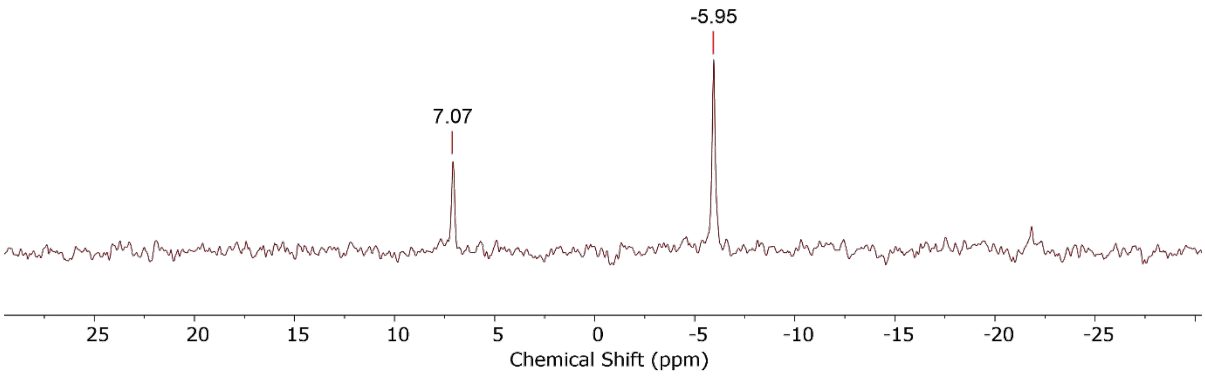

Figure S55. ^{29}Si{^{1}H} NMR spectrum of **4** in d_6-benzene.

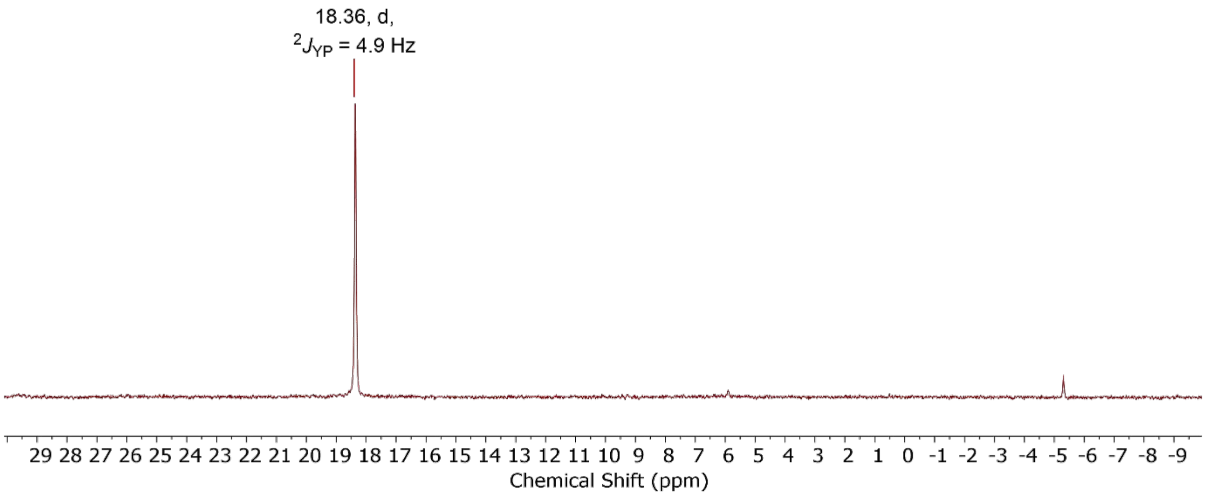

Figure S56. $^{31}P\{^{1}H\}$ NMR spectrum of **4** in d_6-benzene.

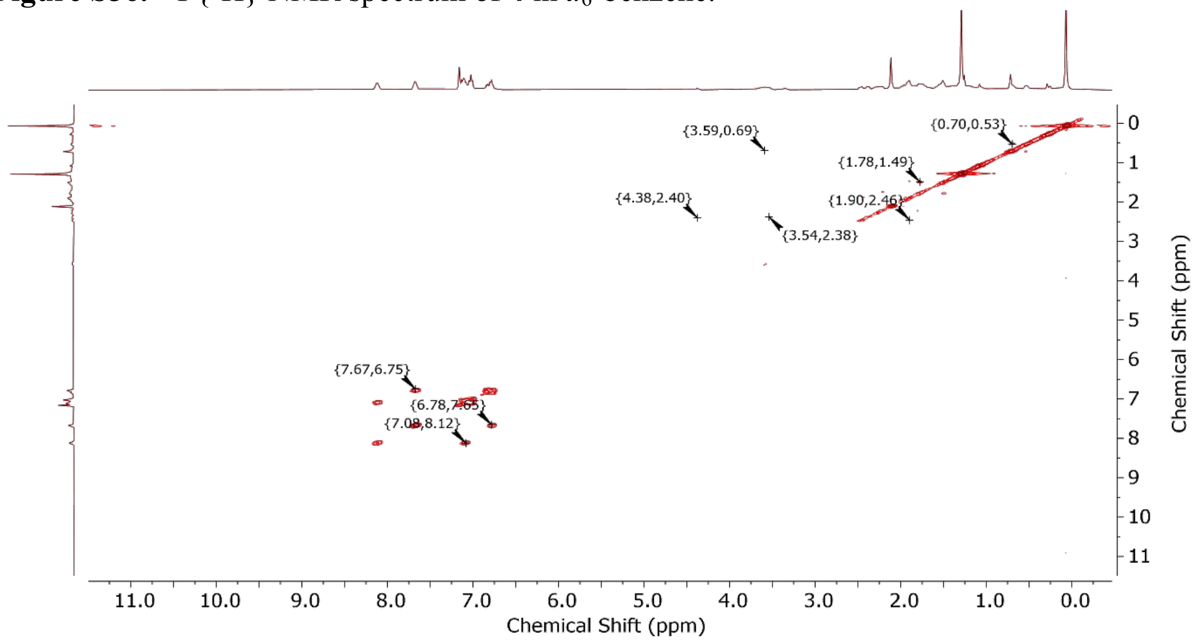

Figure S57. COSY NMR spectrum of **4** in d_6-benzene.

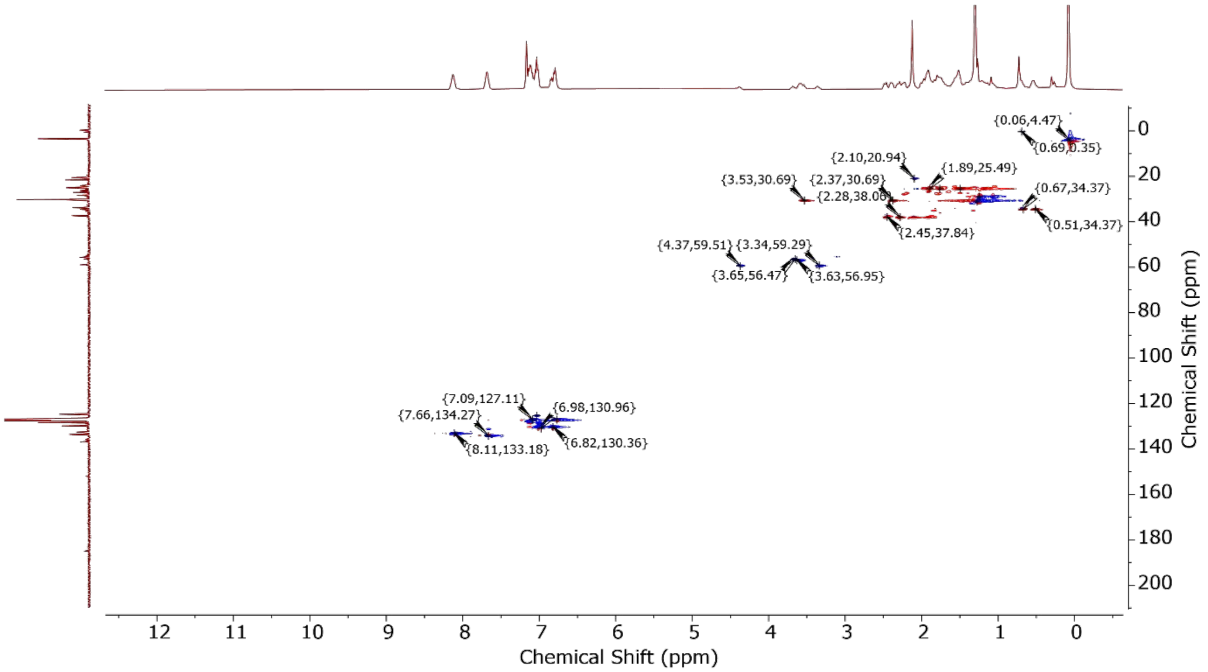

Figure S58. ^{1}H-$^{13}C\{^{1}H\}$ HSQC NMR spectrum of **4** in d_6-benzene.

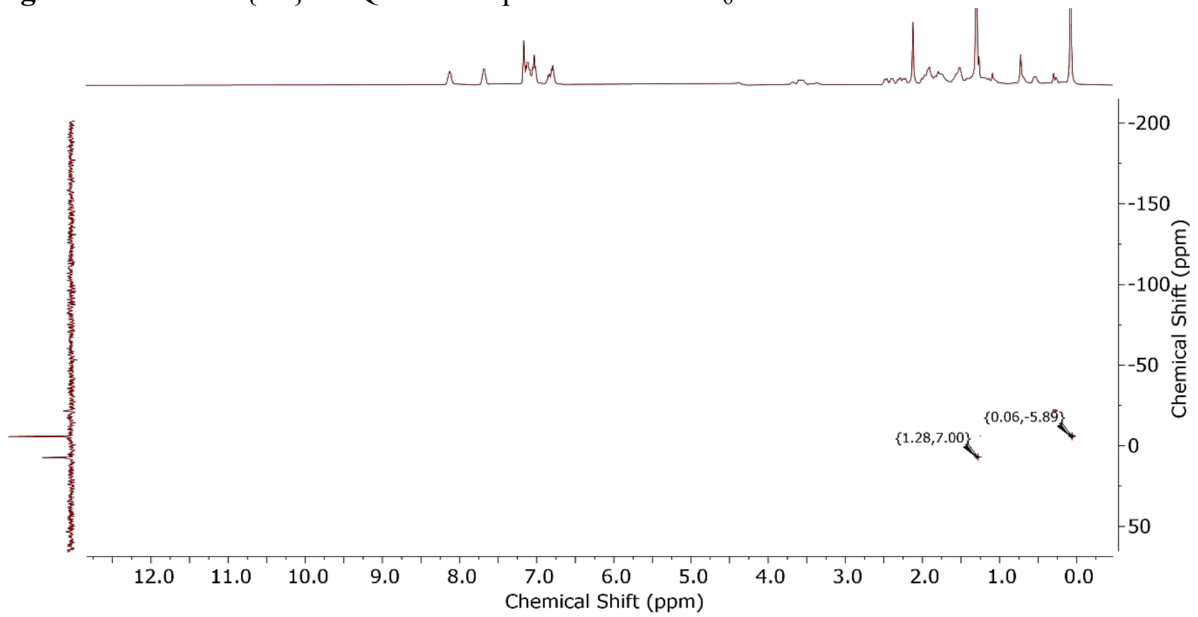

Figure S59. ^{1}H-$^{29}Si\{^{1}H\}$ HMBC NMR spectrum of **4** in d_6-benzene.

7.2. ATR-IR spectrum of 4

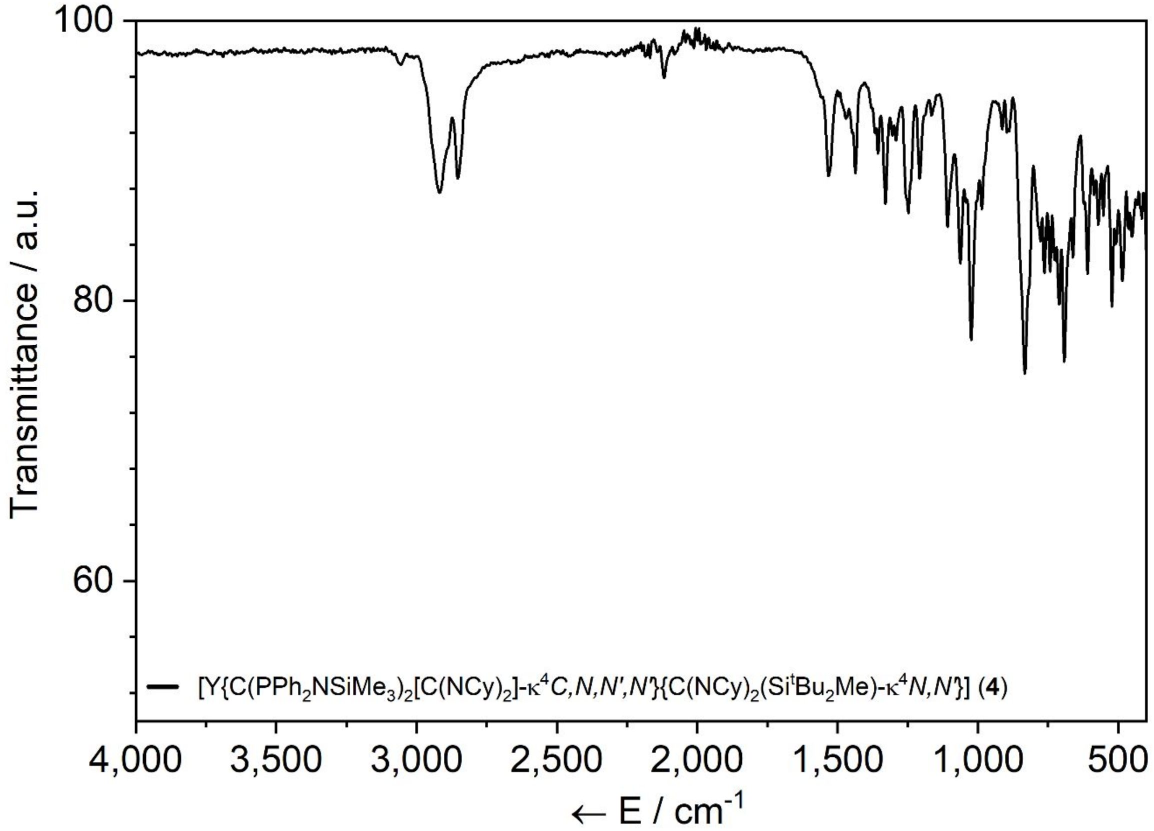

Figure S60. ATR-IR spectrum of **4** between 398-4000 cm^{-1}.
